# CREST functions as a molecular bridge between neuron-specific enhancers and epigenetic regulators in mature motor neurons

**DOI:** 10.1038/s41467-026-78396-x

**Published:** 2026-10-10

**Authors:** Kaitlin N. Montanera, Andrew J. Lee, Woobin Yun, Alyssa Ialongo Del Prete, Ssu-Yu Yeh, Inkyung Jung, Ho Sung Rhee

**Affiliations:** 1 Department of Cell & Systems Biology, University of Toronto, Toronto, ON Canada; 2 Department of Biology, University of Toronto, Mississauga, ON Canada; 3 Department of Biological Sciences, Korea Advanced Institute of Science and Technology (KAIST), Daejeon, Republic of Korea; 4 KAIST InnoCORE AI-CRED Institute, Korea Advanced Institute of Science and Technology (KAIST), Daejeon, Republic of Korea

**Keywords:** Gene regulation, Epigenetics in the nervous system, Chromatin

## Abstract

Motor neuron development requires precise transcriptional regulation mediated in part by BAF (BRG1/BRM-associated factor) chromatin remodeling complexes. The neuron-specific BAF subunit CREST is critical for neuronal function and is mutated in some cases of amyotrophic lateral sclerosis (ALS), yet its underlying genomic mechanisms remain unclear. Using mouse embryonic stem cell differentiation, we show that CREST is essential for motor neuron maturation by activating mature neuronal genes and repressing progenitor genes. Mechanistically, CREST interacts with the neuronal transcription factors ONECUT1 and NGN2, and the BRM ATPase to target pre-existing accessible enhancers of neuronal genes. CREST deletion selectively prevents CBP histone acetyltransferase recruitment, reducing histone H3K27 acetylation at these enhancers and downregulating target gene expression, without disrupting BRM binding, enhancer accessibility, or long-range chromatin architecture. Overall, our findings identify CREST as a crucial epigenetic mediator bridging lineage-specific transcription factors, BAF complexes, and CBP to activate enhancers and promote proper motor neuron development.

## Abstract (web summary)

CREST helps control gene activity for motor neuron development, yet its underlying mechanisms remain unclear. Here, the authors show that CREST coordinates proteins that switch key neuronal genes on and progenitor genes off for healthy motor neurons.

## Introduction

The mammalian central nervous system (CNS) consists of hundreds of thousands of neurons organized in a highly sophisticated manner. The development of this intricate network relies on precisely regulated, cell-type-specific gene expression programs that govern neuronal morphology, connectivity, and functionality^1^. The specification and maintenance of neuronal identity are orchestrated by transcriptional regulatory programs involving specialized transcription factors (TFs)^2,3^, as well as epigenetic mechanisms that modulate DNA methylation, histone modifications, and chromatin remodeling^4^. While significant progress has been made in understanding how TFs and chemical modifications control neuronal gene expression^5–7^, the precise roles of chromatin remodeling proteins during nervous system development remain less understood.

Chromatin remodeling complexes are multi-subunit structures that utilize the energy from ATP hydrolysis to slide, evict, and replace nucleosomes, thereby altering DNA accessibility^8–10^. The mammalian genome encodes four subfamilies of these complexes: ISWI, CHD, INO80, and BAF (mammalian SWI/SNF)^11,12^. BAF complexes consist of at least 15 combinatorial protein subunits, enabling the generation of distinct, cell-type-specific chromatin remodelers^13,14^. Notably, neuronal BAF complexes are the only known chromatin remodelers that incorporate strictly neuron-specific subunits^15,16^. Despite this, it remains largely unknown how neuronal BAF complexes regulate the genomic expression of cell-type-specific genes during neural development.

Motor neurons control essential functions across organs, glands, and muscles^17–19^. Malfunctions in these neurons cause severe defects associated with neurodegenerative diseases, such as ALS and spinal muscular atrophy^20,21^. Calcium-response trans-activator (CREST) is a nuclear transcriptional co-activator and a known neuron-specific subunit of BAF complexes^22,23^. Previous studies revealed that inhibiting CREST incorporation into BAF complexes impairs neuronal function, leading to defective dendritic outgrowth^24,25^. Moreover, genome-wide association studies in a few ALS patients have identified genetic mutations in the *CREST* gene^26,27^. However, the direct effect of CREST dysregulation on neuronal gene expression programs during motor neuron differentiation has not been examined.

Chromatin remodeling complexes regulate cell-type-specific gene expression by allowing developmental TFs to access *cis*-regulatory DNA regions, such as enhancers^28–30^. Developmentally regulated genes are largely controlled by these active enhancers^31,32^, which dynamically gain chromatin accessibility and become enriched with epigenetic marks, such as histone H3 lysine 27 acetylation (H3K27ac) and histone H3 lysine 4 mono-methylation (H3K4me1)^33,34^. Active enhancers often control their distant target genes via long-range chromatin interactions^35^. However, it remains unknown how CREST is recruited to cell-type-specific enhancers to modulate accessibility, histone modification, and long-range interactions.

To address these questions, we utilized a stepwise differentiation system to generate mature spinal motor neurons from mouse embryonic stem (ES) cells^36^. Using a CREST knockout (KO) model, we demonstrate that CREST critically activates neuronal and synaptic genes during the transition from neural progenitors to mature motor neurons. Mechanistically, CREST contributes to the activation of mature neuronal enhancers by recruiting CBP histone acetyltransferase to deposit the active H3K27ac mark at enhancers bound by the motor neuron TFs ONECUT1 and Neurogenin-2 (NGN2). Furthermore, CREST selectively co-binds with the BRM ATPase, not BRG1, at accessible neuronal enhancers. Together, our results establish that CREST is essential for mature neuronal gene expression by cooperating with neuron-specific regulatory proteins at specialized enhancers during motor neuron development.

## Results

### CREST is required for the differentiation of ES cells into motor neurons

**Fig. 1:**
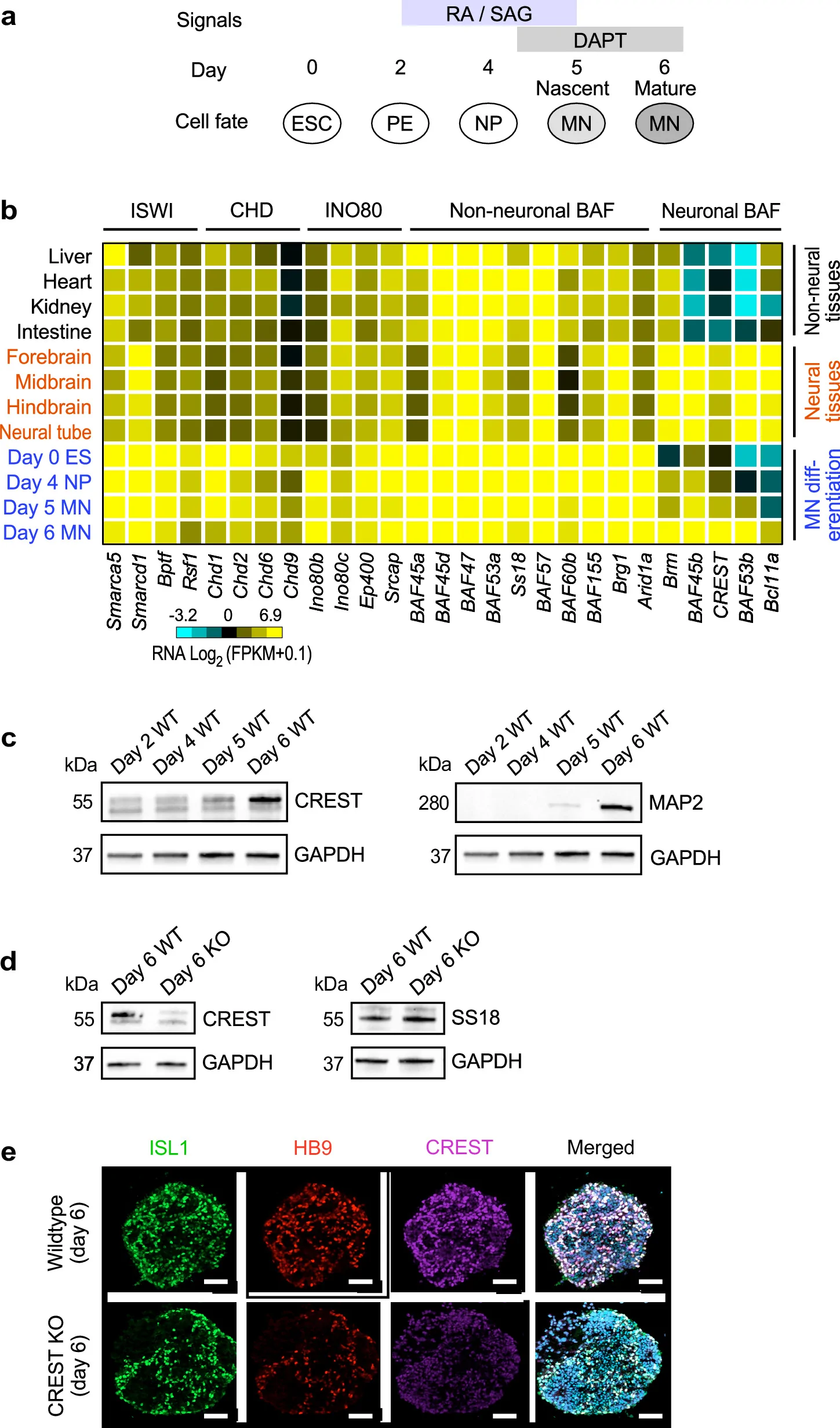
CREST is important for the differentiation of mature motor neurons from mouse ES cells. **a** Overview of mouse embryonic stem cell (ESC)-directed differentiation to postmitotic spinal motor neuron (MN). Differentiating cells become primitive ectoderm (PE) on day 2, neural progenitors (NP) on day 4, nascent MNs on day 5, and mature MNs on day 6. RA, retinoic acid; SAG, smoothened agonist; DAPT, ɣ-secretase inhibitor. **b** Heatmap of RNA expression [log_2_ (FPKM + 0.1)] of selected genes encoding subunit proteins of ATP-dependent chromatin remodeling complexes in postnatal day 0 (P0) mouse tissues (liver, heart, kidney, intestine, forebrain, midbrain, and hindbrain), mouse embryonic day 15.5 (E15.5) neural tube, and at four motor neuron differentiation stages: day 0 ESC, day 4 NP, day 5 nascent MN, and day 6 mature MN (Suppl. Data 1). FPKM (fragments per kilobase of exon per million fragments mapped) values had 0.1 added to avoid producing no value, were median-normalized, and then log_2_-transformed (Suppl. Data 2). FPKM values represent averages from three biological replicates. **c** Western blot analysis confirms that CREST (SS18L1) and the mature neuronal marker MAP2 are detected at ~55 kDa (kilodaltons) and ~280 kDa, respectively, in day 6 WT motor neurons, from two biological replicates. The housekeeping protein GAPDH ( ~ 37 kDa) serves as a loading control (Suppl. Data 3). **d** Western blot analysis confirms that the *CREST* knockout (KO), generated via CRISPR/Cas9, eliminates CREST protein expression in day 6 differentiated cells. CREST is detected at ~55 kDa (upper band) in day 6 WT cells but is absent in day 6 KO cells, from two biological replicates. The CREST paralog, SS18, is detected at ~55 kDa (lower band) in both day 6 WT and day 6 KO cells, from two biological replicates (Suppl. Fig. 2c). **e** Immunostaining of differentiated embryoid bodies, three-dimensional aggregated cells, showing CREST and the motor neuron-specific markers, ISL1 and HB9, in day 6 WT motor neurons and day 6 KO cells, from three biological replicates. Scale bars represent 50 µm.

To identify chromatin remodeling factors expressed during motor neuron development, we performed RNA-seq across multiple stages of in vitro differentiation^36,37^, from day 0 ES cells to day 6 mature motor neurons (Fig. 1a). Assessing subunits of the ISWI, CHD, INO80, and BAF complexes, we found five BAF subunit genes (*Brm, BAF45b, CREST, BAF53b*, and *Bcl11a*) specifically expressed in day 6 motor neurons and other neural tissues (Fig. 1b)^38–40^. Because only CREST is mutated in ALS patients^27,41^, we focused on its specific role in mature motor neuron gene regulation.

Before investigating the functional roles of CREST, we validated our ES cell differentiation system to confirm the successful generation of mature motor neurons. The mature neuronal marker MAP2, the cholinergic neuronal marker ChAT, and CREST were undetectable from the day 2 primitive ectoderm state through the day 5 nascent motor neuron stage, but were specifically expressed in day 6 mature motor neurons (Fig. 1c, Suppl. Fig. 1a). Furthermore, MAP2 was also appropriately localized to dendrites at day 6 (Suppl. Fig. 1b). Other cholinergic or mature motor neuron markers (*VAChT*, *ChT1*, and *Smin32*) were also exclusively expressed on day 6 (Suppl. Fig. 1c). Quantification revealed that 78.7% and 56.5% of day 6 cells were positive with the motor neuron markers ISL1 and HB9, respectively (Suppl. Fig. 1d). Together, these findings demonstrate that our differentiation protocol yields highly pure, cholinergic mature motor neurons, with CREST specifically expressed on day 6.

To examine CREST’s function, we utilized CRISPR/Cas9 to generate a homozygous *CREST* KO mouse ES cell line (*CREST*^–/–^; Suppl. Fig. 2a). Both day 0 wild-type (WT) and KO colonies exhibited similar stem cell pluripotency (Suppl. Fig. 2b). Following differentiation, we confirmed the absence of CREST in day 6 KO cells (Fig. 1d). Because CREST (SS18L1) shares high sequence homology with its paralog SS18 at the amino acid level, we validated our antibody specificity. Western blot analysis showed a residual signal in day 6 KO cells (Fig. 1d), but this matched the background signal of day 2 WT cells (which lack CREST) and coincided with the SS18 signal across replicates (Suppl. Fig. 2c). We concluded that this residual signal represents SS18 cross-reactivity, confirming the successful knockout of *CREST*.

We next investigated whether CREST regulates key cell identity genes. Immunostaining revealed that ISL1 and HB9 expression levels dropped by 56.2% and 89.8%, respectively, in day 6 KO cells compared to day 6 WT cells (Fig. 1e, Suppl. Fig. 2d, e). Using RNA-seq, we confirmed that motor neuron and immature neuronal genes typically are expressed on days 5 and 6, with mature genes reaching peak expression on day 6 (Suppl. Fig. 3a). However, in day 6 KO cells, key motor neuron markers^36^ were downregulated by 59.2–66.6% (Suppl. Fig. 3b). Established immature and mature neuronal markers^42–44^ were similarly downregulated, indicating CREST actively drives neuronal specification. Conversely, neural progenitor and proliferation genes^45,46^, normally highly expressed on day 4 and silenced by day 6, were significantly upregulated in day 6 KO cells (Suppl. Fig. 3c). Combined with our immunostaining data, these findings establish that CREST is strictly required for ES cell differentiation into mature motor neurons by simultaneously activating neuronal genes and repressing progenitor genes.

### Mature neural genes are repressed in day 6 KO cells, potentially relevant to ALS

**Fig. 2:**
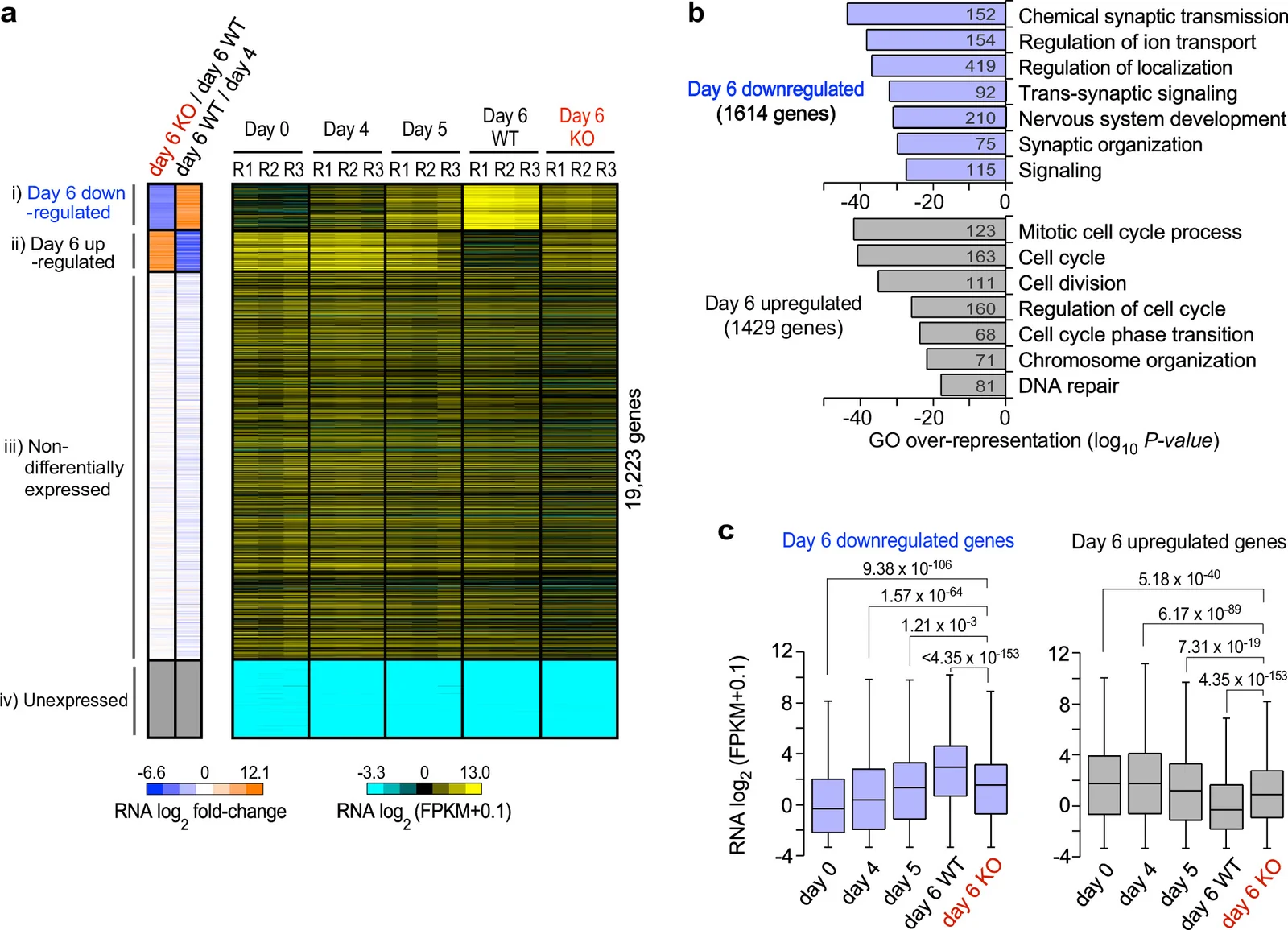
CREST enhances neuronal gene expression during the transition from nascent to mature motor neurons. **a** *Left*: Heatmap of RNA expression (log_2_ fold-changes) of all 19,223 mRNA genes in day 6 *CREST* KO cells compared with day 6 WT mature motor neurons ( > 2-fold-change and *P* < 0.05; determined by the Wald test). Four groups are shown: (i) 1614 downregulated, (ii) 1429 upregulated, (iii) 13,475 non-differentially expressed, and (iv) 2705 unexpressed genes. A heatmap of RNA expression for day 6 WT cells compared with day 4 WT neural progenitors is displayed in the same order as shown in day 6 KO versus day 6 WT comparison. *Right*: Heatmap of RNA expression [log_2_ (FPKM + 0.1)] of all mRNA genes in days 0, 4, 5, and 6 WT cells, and day 6 KO cells during motor neuron differentiation. FPKM values were adjusted by adding 0.1, median-normalized, log_2_-transformed, and ordered as displayed on the *left*. The heatmap includes data from three biological replicates (R1, R2, and R3) derived from independent cell differentiations (Suppl. Data 4). **b** Gene ontology (GO) analysis of 1614 downregulated genes and 1429 upregulated genes identified in panel (**a**) (Suppl. Data 5). The seven most over-represented and non-redundant GO terms are displayed. *P*-values were determined by Fisher’s exact test. Numbers within bars represent the count of genes associated with each GO term (Suppl. Data 6). **c** Box plots showing RNA expression [log_2_ (FPKM + 0.1)] for 1614 downregulated genes and 1429 upregulated genes identified in panel (**a**). FPKM values represent averages from three biological replicates, shown in panel (**a**) (Suppl. Data 7). The plots display the median (line within boxes), first-to-third quartiles (bottom and top of boxes, respectively), and 1.5x the interquartile range (whiskers). *P*-values were determined by a two-sided paired *t*-test.

Next, we assessed whether CREST affects genome-wide gene expression in day 6 motor neurons, coinciding with its peak expression and incorporation into the BAF complex^24^. RNA-seq revealed that 15.8% of all mRNA genes (3043 of 19,223) were significantly altered in day 6 KO cells compared to WT cells (left panel, Fig. 2a). We identified 1614 downregulated genes (Group *i*) and 1429 upregulated genes (Group *ii*). Gene ontology analysis showed that downregulated genes were significantly enriched for mature neuronal functions, including chemical synaptic transmission, signaling, and organization (Fig. 2b). This group included essential genes for post-synaptic plasticity (*Dlg4*)^47,48^, microtubules (*Map6*)^49^, and neuronal differentiation (*Cend1*)^50,51^. In contrast, upregulated genes were strongly enriched for progenitor functions, such as the mitotic cell cycle and cell division. Notably, this included the ALS risk genes *Notch1* and *Ccnd1*^52–54^.

While our primary analyses focused on homozygous mutant cells (*CREST*^–/–^), we also evaluated heterozygous mutants (*CREST*^+/–^), which more closely model ALS-associated mutations. Using CRISPR/Cas9, we generated heterozygous mutant cells and differentiated them into motor neurons (Suppl. Fig. 4a). Like homozygous mutant cells, day 6 heterozygous mutant cells exhibited decreased ISL1 and HB9 expression (Suppl. Fig. 4b). RNA-seq confirmed that global gene expression patterns between the two mutant lines were highly concordant (Suppl. Fig. 4c). Specifically, mature neuronal genes (Group *i*) were downregulated, and cell cycle-related genes (Group *ii*) were upregulated in both lines (Suppl. Fig. 4d), demonstrating that heterozygous *CREST* mutations drive similar, albeit milder, transcriptomic changes.

To validate the clinical relevance of our homozygous mutant model, we compared our RNA-seq data against datasets from human iPSC-derived motor neurons of ALS patients harboring either *C9orf72* or *FUS* mutations^55^. The differentially expressed genes in our day 6 KO cells significantly correlated with the transcriptional alterations induced by both major human ALS mutations (Suppl. Fig. 4e). These findings confirm that *CREST* knockout-induced gene expression changes are highly relevant to human ALS pathology, justifying our use of day 6 KO cells for all subsequent mechanistic analyses.

### Day 6 KO cells are more similar to nascent neurons than progenitors or mature neurons

Since we observed differential expression of progenitor genes and mature neuronal genes, we examined global gene expression changes during the transition from neural progenitors to mature motor neurons. We plotted the log_2_ fold-change in mRNA expression in day 6 WT motor neurons compared to day 4 WT neural progenitors. Interestingly, we found that the expression levels of day 6 differentially expressed genes (Groups *i*, *ii*) were inversely regulated in day 6 motor neurons compared to those in day 4 neural progenitors (left panel, Fig. 2a). The downregulated genes in day 6 KO cells (*CREST*^–/–^) compared to day 6 WT motor neurons (Group *i*) were significantly upregulated during the differentiation from day 4 progenitors to day 6 motor neurons. In contrast, the upregulated genes (Group *ii*) were markedly downregulated during the differentiation from day 4 progenitors to day 6 motor neurons. Overall, our results demonstrate that CREST is involved in the genome-wide repression of day 4 progenitor-specific genes, as well as the activation of day 6 mature neuron-specific genes.

Based on these results, we reasoned that day 6 KO cells might exhibit gene expression patterns similar to day 4 neural progenitors or day 5 nascent neurons. Therefore, we compared the absolute gene expression levels of differentially expressed genes (Groups *i, ii*) in day 6 KO cells with those in days 0, 4, 5, and 6 WT cells (right panel, Fig. 2a). We found that the expression levels of day 6 downregulated genes (Group *i*) in day 6 KO cells were more similar to those of day 5 WT cells than day 4 WT cells (*P* = 1.21 × 10^−^^3^; Fig. 2c). In contrast, the expression levels of these Group *i* genes were significantly different between day 6 KO and day 4 WT cells (*P* < 1.57 × 10^−^^64^). We also observed that the gene expression levels of the day 6 upregulated genes (Group *ii*) in day 6 KO cells were more similar to those of day 5 cells than day 4 cells. We further confirmed that the expression of key marker genes for immature and nascent neurons was similar between day 6 KO and day 5 WT cells (Suppl. Fig. 5). Overall, these results suggest that day 6 KO cells transcriptionally resemble day 5 nascent neurons. Taken together, these findings indicate that CREST mediates the global upregulation of mature neuronal genes and the downregulation of progenitor-specific genes during neuronal maturation. Consequently, in the absence of CREST, ES cells can differentiate into nascent neurons but fail to mature.

### CREST mainly binds to distal cis-regulatory regions of mature neuronal genes

**Fig. 3:**
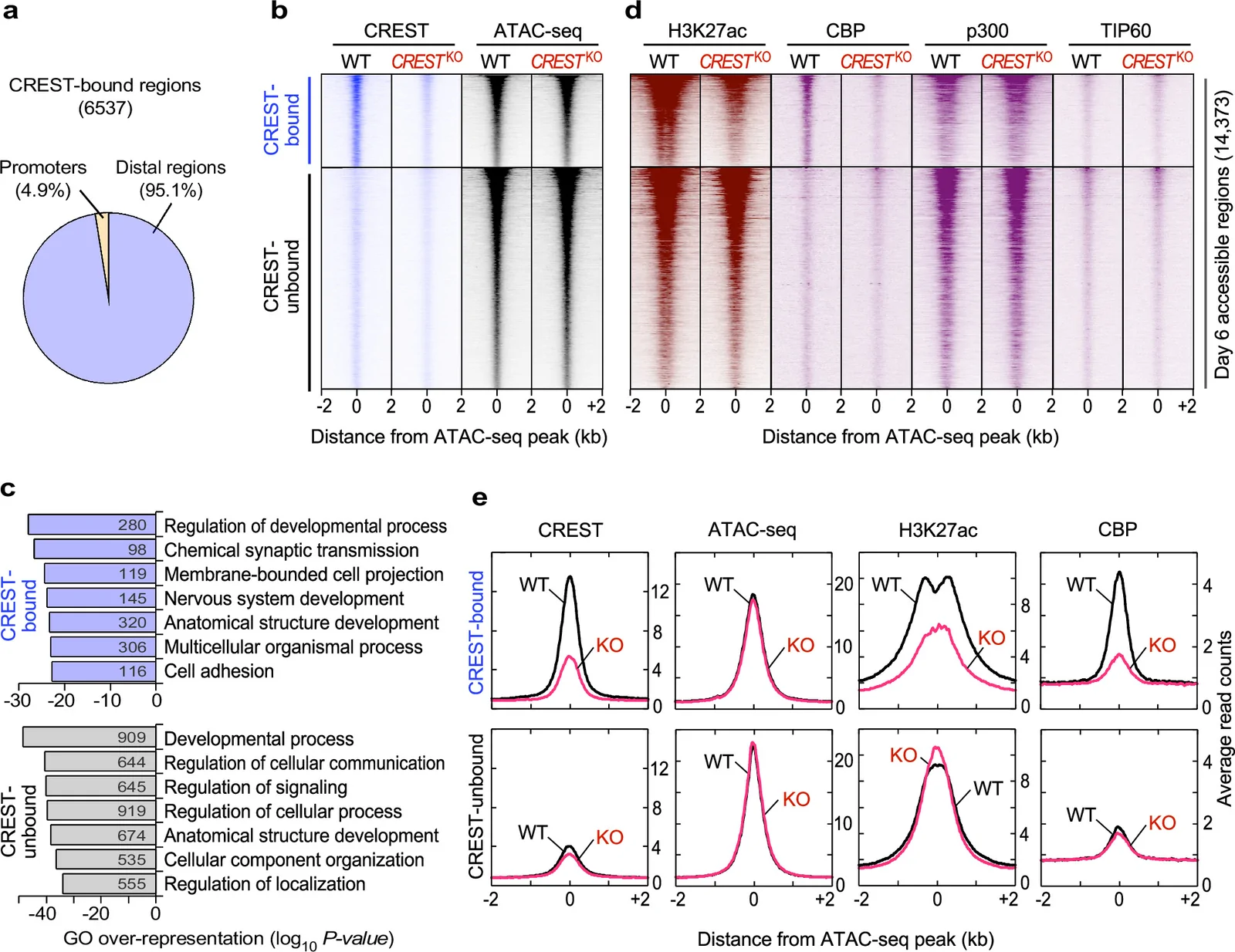
CREST activates neuron-specific distal *cis*-regulatory regions by recruiting CBP to acetylate histone H3K27. **a** Percentages (%) of CREST-bound regions located near gene promoters (322 regions) and at distal *cis*-regulatory regions (6215 regions), detected by ChIP-seq in day 6 WT motor neurons. Genomic regions farther than 3 kb from the nearest transcription start site (TSS) were classified as distal regions. **b** Genomic mapping of ChIP-seq CREST and ATAC-seq in day 6 WT motor neurons and day 6 KO cells. 14,373 highly accessible distal *cis*-regulatory regions were identified from ATAC-seq in day 6 WT motor neurons, grouped by CREST-bound (*n* = 4221 regions) and -unbound distal regions (*n* = 10,152 regions). All data were plotted relative to the ATAC-seq peak midpoints, sorted, and ordered by ATAC-seq intensity in day 6 WT motor neurons (Suppl. Data 8). **c** GO analysis of the 1082 genes associated with CREST-bound distal regions and the 2498 genes associated with CREST-unbound regions, shown in panel (**b**). The seven most over-represented and non-redundant GO terms are displayed. *P*-values were determined by Fisher’s exact test. Numbers within bars represent the count of genes associated with each GO term (Suppl. Data 5, 6). **d** ChIP-seq mapping of H3K27ac and histone acetyltransferases (CBP, p300, and TIP60) in day 6 WT motor neurons and day 6 KO cells at the accessible distal *cis-*regulatory regions identified in panel (**b**). All data were plotted relative to 14,373 ATAC-seq peak midpoints in the same order as shown in panel (**b**). **e** Composite plots (average read counts) of ChIP-seq CREST, H3K27ac, and CBP intensity and ATAC-seq intensity for 4221 CREST-bound and 10,152 CREST-unbound distal regions in day 6 WT motor neurons and day 6 KO cells, shown in panels (**b**, **d**).

During mammalian development, cell type-specific gene regulatory proteins often bind at distal *cis*-regulatory regions, such as genomic enhancers, to control gene expression^56,57^. To determine whether CREST binds at distal *cis*-regulatory regions in day 6 motor neurons, we performed chromatin immunoprecipitation with sequencing (ChIP-seq) for CREST in day 6 motor neurons. Interestingly, we found that CREST binds specifically at distal *cis*-regulatory regions (6215 regions; Fig. 3a), whereas other chromatin remodeling factors bind at promoters as well as distal *cis*-regulatory regions^58,59^. We also found that most CREST-bound *cis*-regulatory regions were markedly distant from the transcription start sites (TSSs) of their closest genes (median distance from the closest TSSs: 84.67 kb; Suppl. Fig. 6a).

One of the major roles of BAF chromatin remodeling complexes is to establish accessible DNA regions. Because CREST is a subunit of neuronal BAF complexes, we hypothesized that CREST would bind to a subset of accessible DNA regions in day 6 motor neurons. To test this, we performed an assay for transposase-accessible chromatin using sequencing (ATAC-seq) in day 6 motor neurons. We identified 14,373 highly accessible distal *cis*-regulatory regions in day 6 cells (Fig. 3b). Among the CREST-bound distal *cis*-regulatory regions, 67.9% (4221 out of 6215) overlapped with these highly accessible regions, whereas 32.1% (1994 out of 6215) were inaccessible. These 1994 inaccessible regions were only weakly bound by CREST (Suppl. Fig. 6b), and their target genes were not enriched for neuronal genes (Suppl. Fig. 6c) and were not specifically expressed in motor neurons compared to CREST-bound accessible regions (Suppl. Fig. 6d). Therefore, we focused our subsequent analyses exclusively on the CREST-bound accessible regions, excluding inaccessible regions.

Next, we examined the biological processes of genes associated with CREST-bound accessible, distal *cis*-regulatory regions (termed ‘CREST-bound distal regions’). For this, we assigned CREST-bound distal regions to the closest TSS of annotated known gene promoters (Suppl. Fig. 6e)^60^, and then performed GO analysis on these genes. GO analysis revealed that the genes linked to CREST-bound distal regions were enriched for neuronal processes, such as chemical synaptic transmission, membrane-bounded cell projection, and nervous system development (Fig. 3c). In contrast, CREST-unbound distal regions are associated with genes involved in non-neuronal, developmental, cellular, and signaling processes. Our finding that CREST mainly binds to distal *cis*-regulatory regions of mature neuronal genes is consistent with our RNA-seq results showing that these genes are significantly downregulated in day 6 KO cells.

### CREST recruits CBP to distal cis-regulatory regions for histone acetylation

Active distal *cis*-regulatory regions, such as enhancers, are marked by epigenetic histone modifications, such as acetylation of histone H3 on lysine 27 (H3K27ac), which can alter chromatin structure to activate gene expression^61,62^. To determine whether histones near CREST-bound distal regions are epigenetically activated, we performed ChIP-seq for the active enhancer-associated histone mark H3K27ac in day 6 motor neurons. We found that H3K27ac levels were highly enriched at both CREST-bound and -unbound distal regions in day 6 motor neurons (Fig. 3d). Next, we examined whether the knockout of *CREST* affects H3K27ac levels at day 6 CREST-bound distal regions by comparing the H3K27ac levels at CREST-bound distal regions between day 6 WT motor neurons and day 6 KO cells. Interestingly, we found that H3K27ac levels at CREST-bound distal regions were significantly decreased following *CREST* knockout (*P* = 3.08 × 10^−^^24^; Suppl. Fig. 7a), while H3K27ac levels at CREST-unbound distal regions were maintained. These results suggest that CREST may be important for the enrichment of the active enhancer mark, H3K27ac, at distal *cis*-regulatory regions.

We then investigated which histone lysine acetyltransferase (KAT) is involved in the specific activation of these CREST-bound distal regions. To determine this, we performed ChIP-seq for three KATs known to deposit H3K27ac: CREB-binding protein (CBP, also known as KAT3A), E1A-binding protein p300 (also known as KAT3B), and TIP60 (also known as KAT5)^63^. Our ChIP-seq analysis revealed that CBP specifically co-localized with CREST at CREST-bound distal regions, whereas p300 binding was enriched at both CREST-bound and CREST-unbound distal regions (Fig. 3d). Furthermore, we found that TIP60 binds at neither CREST-bound nor CREST-unbound distal regions; instead, it localizes to CREST-unbound promoter regions (Suppl. Fig. 7b). These findings indicate that CBP, rather than p300 or TIP60, exhibits specificity for CREST-bound distal regions in day 6 motor neurons.

Interestingly, CBP binding was notably decreased at CREST-bound distal regions in day 6 KO cells (Fig. 3d, e). In contrast, p300 enrichment at these distal regions and TIP60 enrichment at promoters remained unchanged (Suppl. Fig. 7b), indicating no functional association between CREST binding and p300/TIP60 recruitment. Because H3K27ac levels at CREST-bound regions were not completely abolished in KO cells, p300 likely maintains residual H3K27ac independently of CREST. Furthermore, *CREST* knockout reduced CBP mRNA levels by 31.5% (Suppl. Fig. 7c), suggesting that the absence of CREST may partially decrease CBP expression or its genomic binding. Therefore, CREST preferentially recruits CBP to acetylate histone H3 at lysine 27 within accessible distal *cis*-regulatory regions in day 6 motor neurons.

We next utilized ATAC-seq to examine whether *CREST* knockout alters chromatin accessibility. Accessibility at CREST-bound distal regions remained largely unchanged in day 6 KO cells compared to WT cells (Fig. 3b, e). This aligns with prior findings that CREST acts as a non-catalytic subunit within BAF chromatin remodeling complexes^23^. We confirmed the reproducibility of all ATAC-seq and ChIP-seq data across independent differentiations (Suppl. Fig. 8a, b). Together, these findings demonstrate that rather than establishing accessible distal *cis*-regulatory regions, CREST facilitates the activation of these neuron-specific enhancers by recruiting the histone acetyltransferase CBP.

### CREST activates neuronal gene expression through long-range chromatin interactions

**Fig. 4:**
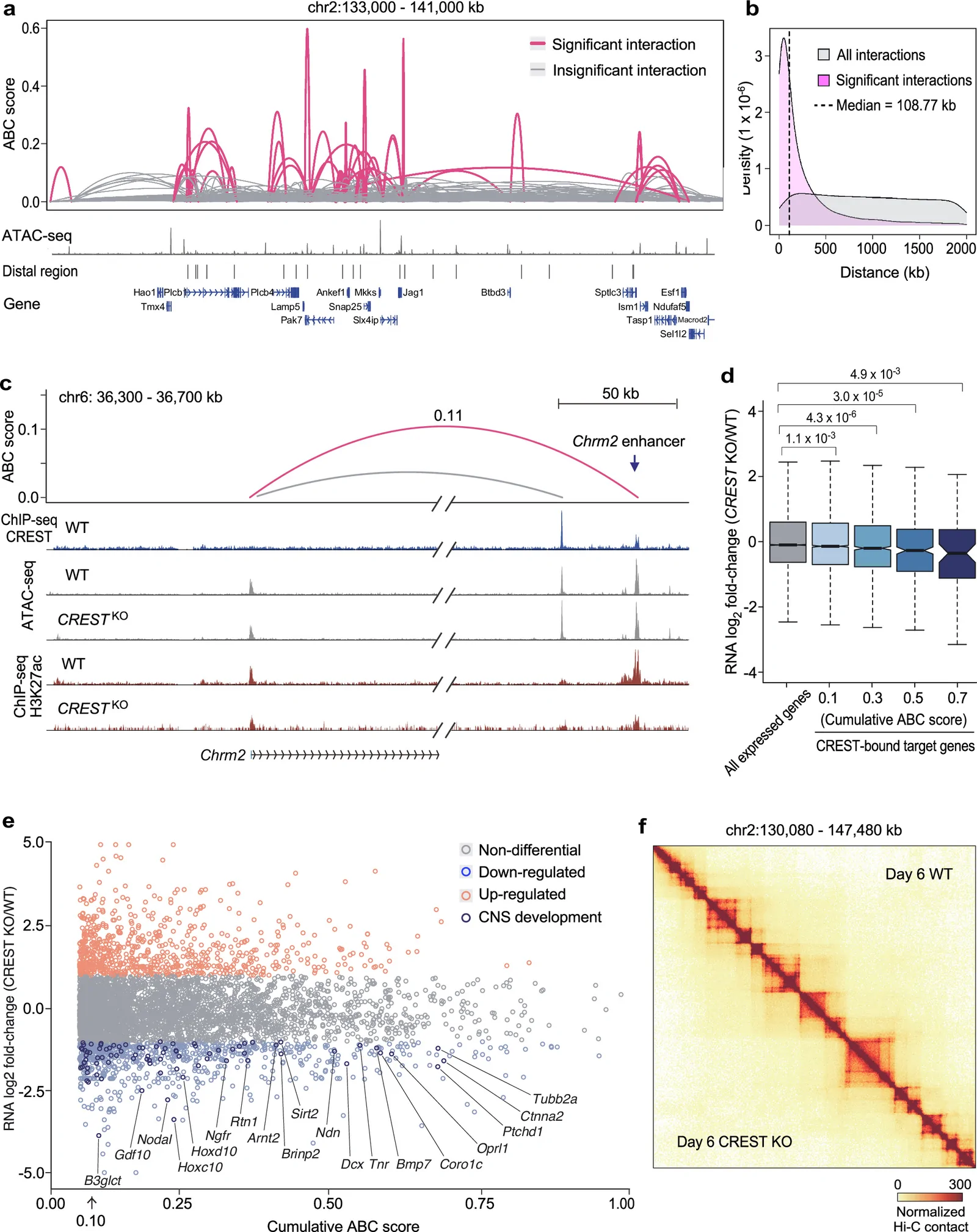
High-resolution, long-range *cis*-regulatory network deciphers CREST-dependent gene expression. **a** Representative chromatin interaction profile of day 6 WT motor neurons based on activity-by-contact (ABC) score at 10 kb resolution, with tracks of ATAC-seq signals, accessible regions, and genes. The chromatin interactions between genes and accessible regions with an ABC score > 0.10 are considered significant, as shown by  magenta arcs. **b** Density plot showing the distribution of genomic distances for significant (magenta) and all (grey) gene-to-accessible DNA region interactions. **c** The location of CREST-bound distal region, detected by ChIP-seq CREST, H3K27ac, and ATAC-seq, for *Chrm2* gene in day 6 WT and day 6 KO cells. The CREST-bound distal region is linked to its target gene by a significant long-range chromatin interaction (magenta arc). The number (0.11) above the arc indicates an ABC score. **d** Notched boxplots describing the RNA expression (log_2_ fold-changes) of CREST-bound target genes compared to all expressed genes (*n* = 19,223). Differences in gene expression between CREST-bound target genes were grouped by cumulative ABC score thresholds ( > 0.1, *n* = 5320; > 0.3, *n* = 1,578; >0.5, *n* = 447; and > 0.7, *n* = 102), from two biological replicates (Suppl. Data 7). The plots display the median (center line), first-to-third quartiles (bottom and top of boxes, respectively), and 1.5x the interquartile range (whiskers). *P*-values were determined by a two-sided Wilcoxon rank-sum test. **e** A scatter plot showing the RNA expression (log_2_ fold-changes) of CREST-bound target genes in relation to the cumulative ABC scores of significant interactions harbored by the CREST-bound distal regions. Genes related to central nervous system (CNS) development are annotated in black. Genes with a log_2_ fold-change > 1 and genes with a log_2_ fold-change < −1 for day 6 KO cells compared with day 6 WT motor neurons are considered upregulated contacts (769 genes; orange) and downregulated contacts (884 genes; blue), respectively. The preferential inclusion of differential contacts was assessed using the two-sided exact binomial test (*P* = 5.79 × 10^−^^6^). **f** Normalized Hi-C contact maps of day 6 KO cells and day 6 WT motor neurons at 40 kb resolution.

To investigate how cell-type-specific enhancers regulate gene promoters, we performed a high-throughput chromosome conformation capture (Hi-C) assay in day 6 motor neurons (Fig. 4a). Applying the activity-by-contact (ABC) model^64^ to 462 million Hi-C read-pairs revealed 39,808 significant chromatin interactions with a median distance of 108.77 kb (Fig. 4b, Suppl. Fig. 9a). Notably, CREST bound many of these interacting distal regions—including those regulating mature neuronal genes, such as *Chrm2, Ntf3*, and *Oprl1*, which exhibited decreased H3K27ac levels upon *CREST* knockout (Fig. 4c, Suppl. Fig. 9b). Using the ABC model, we linked CREST-bound distal regions to 5320 significantly interacting target genes, which proved to be far more comprehensive than simply assuming these regions target the genes closest to them (Suppl. Fig. 10a). Specifically, this approach retained 76.3% of the targets identified by genomic proximity–based assignment while substantially expanding the explanatory power, identifying 36.1% (1098 of 3043) of all differentially expressed genes as CREST-bound targets compared with only 11.9% captured by genomic proximity alone (Suppl. Fig. 10b).

We assessed the expression of CREST-bound target genes in day 6 KO cells compared to WT cells, confirming global downregulation in the KO cells. Notably, target genes with stronger chromatin interactions with CREST-bound regions exhibited significantly greater downregulation upon *CREST* knockout (Fig. 4d), indicating that CREST-bound chromatin contacts critically influence target gene expression. Furthermore, these target genes were highly enriched for CNS developmental genes, including *Tubb2a, Ctnna2, Ptchd1*, and *Oprl1* (Fig. 4e). Mutations in *Ptchd1* and *Oprl1* are linked to neurodevelopmental disorders^65,66^. Overall, 33.2% of annotated CNS developmental genes (363 of 1093) significantly interact with CREST-bound distal regions in day 6 motor neurons (Suppl. Fig. 10c).

Finally, we utilized Hi-C data to determine if CREST structurally alters chromatin interactions between distal *cis*-regulatory regions and target promoters. Comparing genomic contacts in day 6 KO and WT cells revealed no significant changes in the global chromatin contact landscape (Fig. 4f, Suppl. Fig. 11). For differentially expressed genes, only a negligible fraction of chromatin contacts—0.16% for downregulated and 0.12% for upregulated genes—showed significant structural changes following knockout (*P* < 0.01; Suppl. Fig. 12a). Additionally, these structural changes were not significantly more frequent at CREST-bound regions versus -unbound regions (Suppl. Fig. 12b). Therefore, we conclude that CREST-bound enhancers modulate neuronal gene expression by utilizing pre-existing long-range chromatin interactions rather than actively reorganizing the genomic architecture.

### Neuron stage-specific accessibility correlates with, but is independent of, CREST binding

**Fig. 5:**
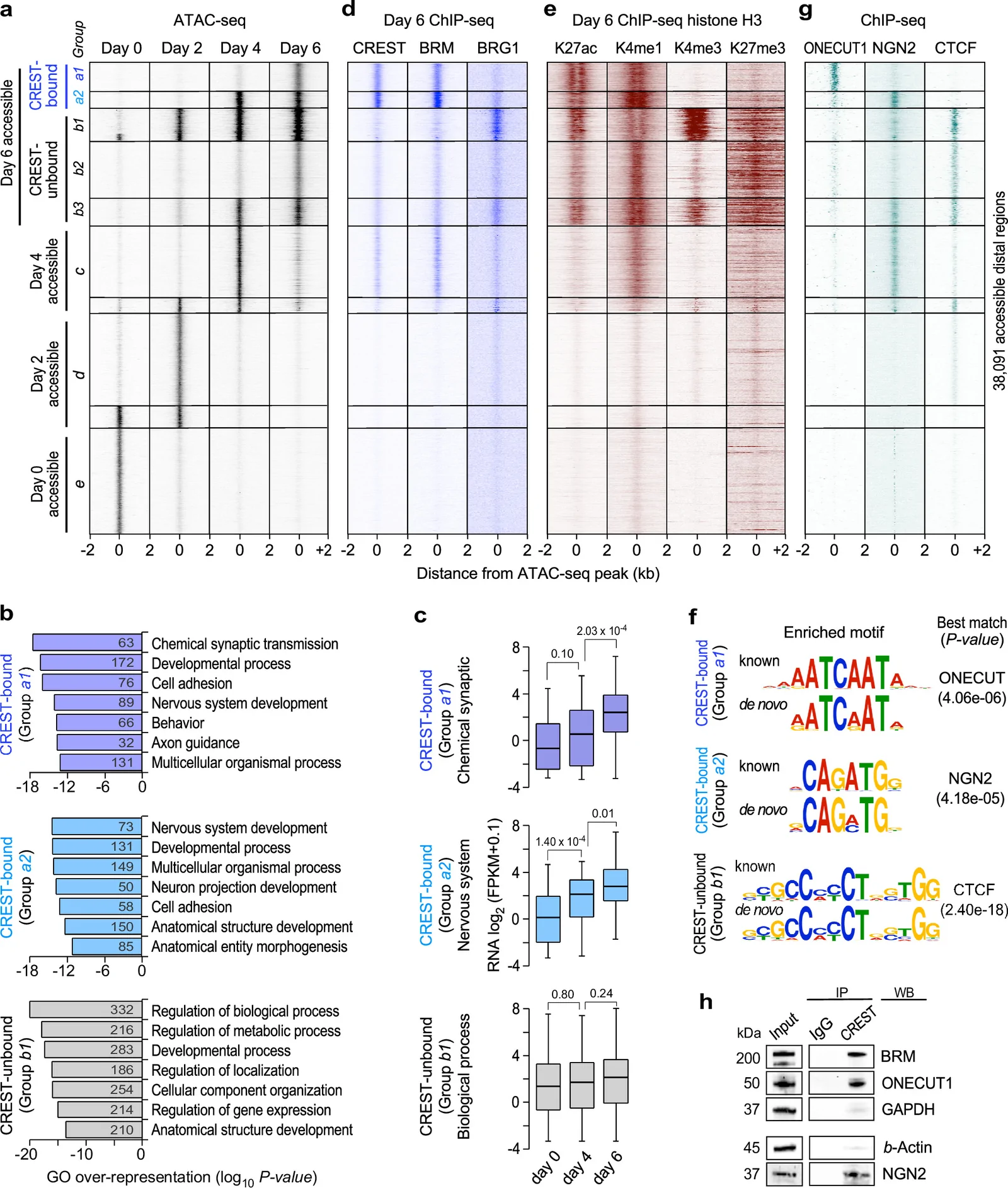
CREST recognizes enhancers bound by BRM ATPase and motor neuron TFs. **a** ATAC-seq mapping of accessible distal regions during motor neuron differentiation, grouped by CREST-bound day 6 accessible (*a1*), day 4 and day 6 accessible (*a2*), CREST-unbound accessible from day 2 to day 6 (*b1*), accessible regions in day 6, day 4, day 2, and day 0 cells (groups *b2, c, d*, and *e*) (Suppl. Data 9). **b** GO analysis of the genes associated with Groups *a1, a2*, and *b1* (673, 487, and 755 genes, respectively), shown in panel (**a**). *P*-values were determined by Fisher’s exact test. Numbers within bars represent the count of genes in each term (Suppl. Data 5, 6). **c** Box plots showing gene expression in the top GO terms in Groups *a1, a2*, and *b1* (63, 73, and 332 genes, respectively), described in panel (**b**). One-way analysis of variance (ANOVA) followed by Tukey’s post hoc tests: Group *a1, F*(2,6) = 19.5, *P* = 2.05 × 10^−8^; Group *a2*, *F* (2,6) = 25.6, *P* = 1.06 × 10^−10^; and Group *b1*, *F* (2,6) = 2.71, *P* = 0.067 (Suppl. Data 7, 10). **d**, **e** ChIP-seq mapping of CREST, BRM, BRG1, and histone modifications at the accessible distal regions. All data were plotted in the same order as shown in panel (**a**). **f** De novo DNA motif analysis within 100 bp of ATAC-seq peak midpoints in Groups *a1, a2*, and *b1*. *P*-values were determined by Fisher’s exact test. **g** Mapping of ChIP-exo ONECUT1 and ChIP-seq CTCF in day 6 motor neurons, and ChIP-seq NGN2 in day 4 neural progenitors at the accessible distal regions. All data were plotted in the same order as shown in panel (**a**). **h** Immunoprecipitation (IP) of day 6 cells with anti-CREST and IgG (control) antibodies, followed by Western blot (WB) for BRM, ONECUT1, NGN2, and controls (GAPDH and β-Actin). Bands were detected for BRM ( ~ 200 kilodaltons or kDa), ONECUT1 ( ~ 50 kDa), NGN2 ( ~ 37 kDa), GAPDH ( ~ 37 kDa), and β-Actin ( ~ 45 kDa), from two biological replicates. The input consists of 25 µg of nuclear extract (Suppl. Data 3).

Since the accessibility of day 6 CREST-bound distal regions is independent of CREST binding (Fig. 3), we examined whether CREST binding correlates with chromatin accessibility during motor neuron differentiation. To test this, we examined chromatin accessibility at four developmental stages of the differentiation: ES cells on day 0, primitive ectoderm-like cells on day 2, neural progenitors on day 4, and mature motor neurons on day 6 using ATAC-seq. Because CREST primarily binds to distal *cis*-regulatory regions, rather than gene promoters, we examined DNA accessibility only at these regions. We divided the accessible distal *cis*-regulatory regions into five groups: *a*) day 6 accessible CREST-bound, *b*) day 6 accessible CREST-unbound, *c*) day 4 accessible, *d*) day 2 accessible, and *e*) day 0 accessible (Fig. 5a). Interestingly, we found that 60.8% of day 6 CREST-bound distal regions (2236 out of 3678; Group *a1*) were accessible only in day 6 motor neurons, while 39.2% of day 6 CREST-bound distal regions (1442 out of 3678; Group *a2*) were already accessible in day 4 neural progenitors. Gene ontology analysis revealed that the CREST-bound regions only accessible on day 6 (Group *a1*) were significantly associated with mature neuronal genes, such as chemical synaptic transmission genes (Fig. 5b). On the other hand, the CREST-bound regions accessible on both day 4 and day 6 (Group *a2*) were linked to early neuronal genes, such as nervous system developmental genes. We also found that the expression of mature neuronal genes bound by CREST (Group *a1*) was significantly upregulated from day 4 neural progenitors to day 6 mature motor neurons (*P* = 2.03 × 10^−4^; Fig. 5c) whereas expression of the early neuronal genes bound by CREST (Group *a2*) was not significantly upregulated from day 4 to day 6 motor neurons, instead, they were significantly upregulated from day 0 ES cells to day 4 neural progenitors (*P* = 1.40 × 10^−4^; Fig. 5c).

These results suggest that the expression of the mature neuronal genes (Group *a1*) was activated by CREST binding during neuronal maturation from day 4 to day 6 of the differentiation, whereas the expression of early neuronal genes (Group *a2*) was activated during neural development before CREST binding. In addition, day 6 CREST-unbound regions, mainly accessible from day 2 cells to day 6 mature neurons (Group *b1*), were associated with non-neuronal housekeeping genes involved in biological and metabolic processes, which were similarly expressed from day 0 to day 6 (Fig. 5b, c). Together, our results demonstrate that CREST binding correlates with chromatin accessibility, although chromatin accessibility is not dependent on CREST binding, and its target neuronal genes are expressed in a neurodevelopmental stage-specific manner during motor neuron differentiation.

### BRM ATPase is involved in the chromatin accessibility of CREST-bound enhancers

BAF complexes utilize either BRG1 or BRM ATPases to establish chromatin accessibility^67,68^. Because both are highly expressed in day 6 motor neurons (Fig. 1b), we used ChIP-seq to determine which ATPase co-localizes with CREST. We found that CREST globally co-occupies with BRM at accessible distal *cis*-regulatory regions (Fig. 5d). Specifically, BRM occupancy is significantly enriched at CREST-bound regions (Groups *a1*, *a2*) compared to CREST-unbound regions (*P* = 1.44 × 10^−80^; Suppl. Fig. 13a). Conversely, BRG1 is significantly enriched at a subset of CREST-unbound regions (Group *b1*; *P* = 2.30 × 10^−43^). Remaining CREST-unbound regions lacked both ATPases, suggesting the involvement of other remodelers.

Integrated with GO data (Fig. 5b), our results reveal that BRM primarily drives the accessibility of CREST-bound distal regions associated with neuronal genes, whereas BRG1 regulates CREST-unbound regions linked to housekeeping genes. This indicates that BRM and CREST assemble within the same neuronal BAF complexes in motor neurons, while BRG1 may function in other developmental contexts or neuronal subtypes^23^. Importantly, BRM binding at CREST-bound distal regions remained unchanged in day 6 KO cells compared to WT cells (Suppl. Fig. 13b). This demonstrates that CREST does not recruit BRM to these distal regions, explaining why chromatin accessibility at these sites remains unaltered following CREST knockout. Combined with our Hi-C results (Fig. 4f, Suppl. Fig. 11), these findings show that *CREST* knockout does not disrupt BRM binding, enhancer accessibility, and the underlying long-range chromatin architecture at neuronal enhancers. Instead, the primary role of CREST is to recruit CBP for enhancer activation.

We next examined whether specific histone modifications enrich CREST-bound and -unbound distal regions to control enhancer activity. Day 6 CREST-bound regions (Groups *a1, a2*) were notably enriched with active and primed enhancer marks (H3K27ac, H3K4me1) but completely lacked the repressive H3K27me3 mark (Fig. 5e). This supports our finding that CREST recruits CBP to establish H3K27ac, thereby activating these regions. In contrast, CREST-unbound regions (Groups *b1–b3*) were enriched with H3K27me3. A subset of these unbound regions (Groups *b1, b3*) contained both activating (H3K4me3) and repressing (H3K27me3) marks, forming bivalent chromatin domains linked to poised gene expression^69^. Additionally, day 2-accessible CREST-unbound regions (Group *b1*) uniquely exhibited RNA polymerase II phosphorylated at serine 5, marking paused polymerase or enhancer RNA^70^, which was absent in CREST-bound regions (Suppl. Fig. 13b). All ATAC-seq and ChIP-seq results were reproducible across independent differentiations (Suppl. Fig. 14). Because the day 6 CREST-bound distal regions are accessible, enriched with H3K27ac, and activate their interacting target neuronal genes in a neurodevelopmental stage-specific manner, we termed these regions ‘CREST-bound enhancers’.

### CREST is recruited to ONECUT1- or NGN2-bound neuronal enhancers

Next, we investigated how CREST-containing BAF complexes are recruited to neuronal enhancers in day 6 motor neurons. Because cell type-specific TFs often interact with BAF subunits at accessible distal *cis*-regulatory regions^13,71,72^, we hypothesized that CREST-bound accessible enhancers contain distinct TF-binding motifs that drive this recruitment. Using MEME de novo DNA motif discovery^73^, we analyzed day 6 CREST-bound and -unbound distal regions (Fig. 5f). Interestingly, day 6-specific CREST-bound enhancers (Group *a1*) were significantly enriched with the maturing motor neuron-specific ONECUT1 TF motif^36^. Conversely, CREST-bound enhancers accessible on both days 4 and 6 (Group *a2*) were enriched with the neural progenitor-specific NGN2 TF motif^74^. Additionally, day 6 CREST-unbound distal regions (Group *b1*) were enriched for the ubiquitous CTCF TF motif^75,76^.

To validate these findings, we mapped the genomic binding of ONECUT1 and CTCF in day 6 motor neurons, and NGN2 in day 4 neural progenitors (Fig. 5g). As predicted, ONECUT1 bound only to day 6-specific CREST-bound enhancers (Group *a1*), while NGN2 bound to day 4 and day 6 accessible CREST-bound enhancers (Group *a2*). In contrast, CTCF exclusively occupied day 6 CREST-unbound distal regions (Groups *b1*–*b3*). These ChIP-seq results suggest that the motor neuron TF ONECUT1 and the progenitor TF NGN2 recruit CREST-containing BAF complexes to their respective stage-specific enhancers. Furthermore, CREST is excluded from CTCF-bound distal regions associated with housekeeping genes (Group *b1*; Fig. 5b, g). This aligns with our previous finding that neuron-specific enhancers lack CTCF binding, instead relying on neuron-specific TFs^77^. We further utilized co-immunoprecipitation (IP) to determine whether CREST interacts with ONECUT1 and NGN2, as well as BRM ATPase. We confirmed the physical interactions between CREST and these TFs in day 6 motor neurons (Fig. 5h). This co-IP result also confirmed that BRM and CREST assemble within the same neuronal BAF complexes. Overall, these results support the conclusion that CREST is recruited to developmentally regulated neuronal enhancers via interactions with stage-specific neuronal TFs.

### CREST-bound enhancers control the transcription of key mature neuronal genes

**Fig. 6:**
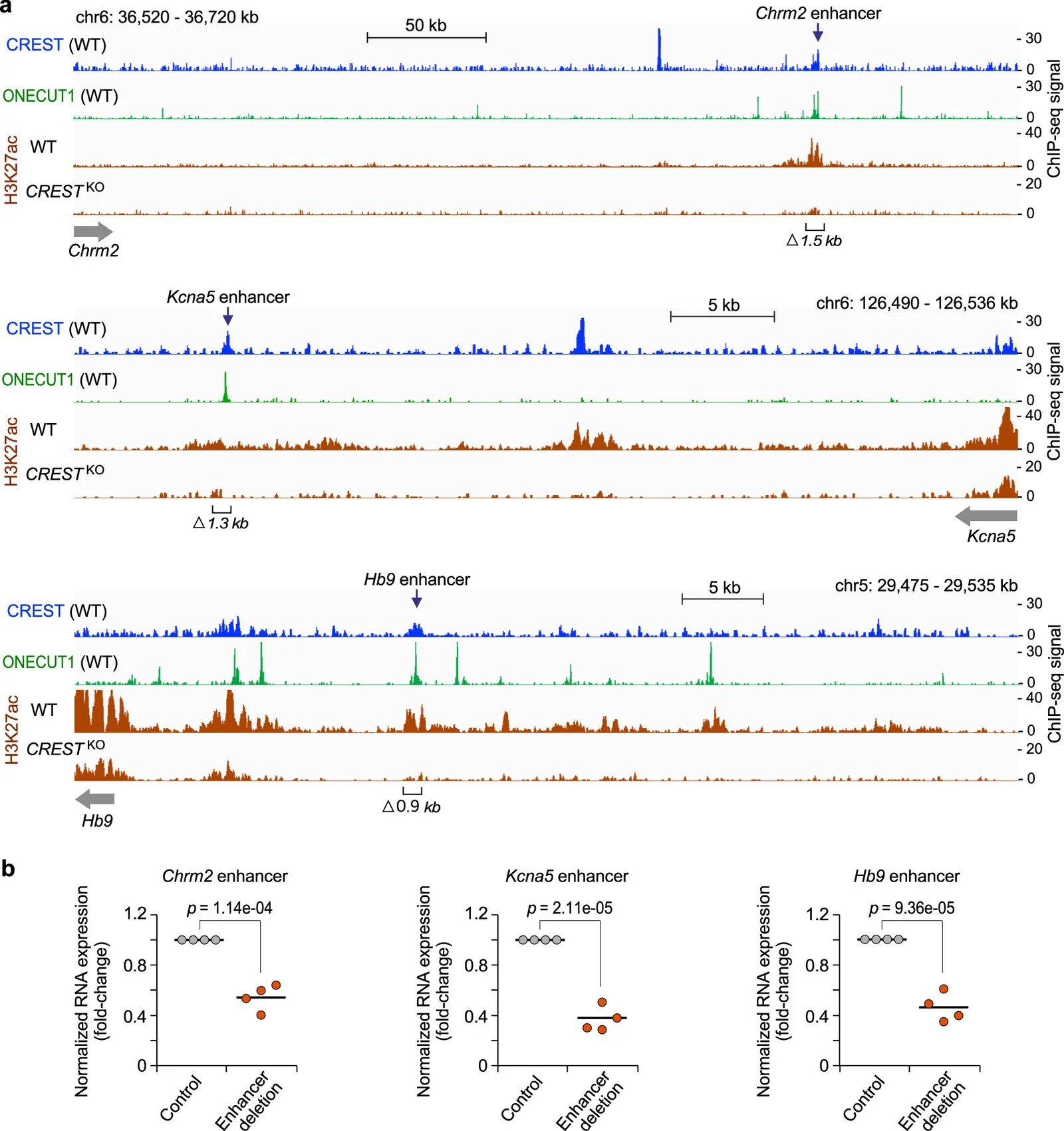
CREST-bound enhancers control the transcription of key mature neuronal genes. **a** The locations of CREST-bound enhancers of *Chrm2* (*upper*), *Kcna5* (*middle*), and *Hb9* (*lower*) genes, detected by ChIP-seq CREST and ChIP-exo ONECUT1 in day 6 WT motor neurons, and ChIP-seq H3K27ac in day 6 WT motor neurons and day 6 KO cells. These CREST/ONECUT1 co-bound enhancers (arrows) were deleted in ES cells using CRISPR/Cas9 genome editing before a six-day motor neuron differentiation. **b** Fold-change in the expression of neuronal genes, *Chrm2, Kcna5*, and *Hb9*, measured by RT-qPCR in day 6 mutant cells harboring deletions of their respective CREST-bound enhancers (indicated by arrows in panel (**a**); 1.5 kb for *Chrm2*, 1.3 kb for *Kcna5*, and 0.9 kb for *Hb9*). Expression levels are normalized to day 6 WT motor neurons. *P*-values were calculated by a two-sided, two-sample Student’s *t*-test (two cell differentiations with two replicates, *n* = 4 independent experiments). Lines in the dot plots show the average (Suppl. Data 7).

To test the requirement of CREST-bound enhancers for neuronal gene expression during motor neuron differentiation, we utilized the CRISPR/Cas9 system to generate mutant ES cell lines lacking specific CREST-bound enhancers^77^. We targeted genomic regions containing enhancers interacting with promoters of key mature neuronal genes, identified by Hi-C (Fig. 4), including the cholinergic receptor *Chrm2*, the potassium voltage-gated channel *Kcna5*, and the motor neuron-specific gene *Hb9*. In day 6 motor neurons, these specific CREST-bound enhancers were co-occupied by the motor neuron TF ONECUT1 and lost the active H3K27ac histone mark upon *CREST* knockout (Fig. 6a). Following the differentiation of WT and mutant ES cells into day 6 cells, RT-qPCR analysis revealed that deleting these enhancers significantly decreased target gene transcription by 45.6% to 61.7% (*P* < 1.14 × 10^−4^; Fig. 6b). We observed similarly significant downregulation when deleting CREST-bound enhancers interacting with the neurotrophin gene *Ntf3* and the G-protein-coupled receptor *Oprl1* (*P* < 2.88 × 10^−5^; Suppl. Figs. 9b, 15a). Complete target gene silencing was not achieved by single enhancer deletions, which is expected since neuronal genes are typically regulated by multiple redundant enhancers^77^. We next assessed whether targeted enhancer deletions disrupt physical neuronal differentiation. We found that *Chrm2* enhancer deletion severely impaired axon outgrowth, as visualized by TUBB3 staining, and *Kcna5* enhancer deletion disrupted the proper dendritic localization of MAP2, a mature neuron marker (Suppl. Fig. 15b). These results suggest that CREST-bound enhancers may drive the transcription of mature neuronal genes and the phenotypic changes required for proper neuronal maturation.

Additionally, we investigated whether *CREST* knockout permanently prevents or merely delays mature neuronal gene expression. Extending differentiation to day 9, we performed RNA-seq on WT and *CREST* KO cells. We focused on 1614 genes downregulated in day 6 KO cells (Group *i*, Fig. 2a), subdividing them into two subgroups by comparing their expression in day 9 mature versus day 5 nascent WT motor neurons: Group *1a* (879 genes; unchanged or downregulated on day 9) and Group *1b* (735 genes; upregulated on day 9; Suppl. Fig. 16a). GO analysis confirmed that Group *1b* genes are significantly enriched for mature neuronal functions, including chemical synaptic transmission (Suppl. Fig. 16b). As expected, these genes were significantly downregulated in day 6 KO cells compared to WT cells. However, their expression fully recovered in day 9 *CREST* KO cells, reaching WT-comparable levels (Suppl. Fig. 16c). This demonstrates that *CREST* knockout delays, rather than strictly prevents, neuronal maturation, suggesting other neuron-specific factors compensate for CREST during later developmental stages.

### CREST knockout downregulates the expression of specific neuronal BAF subunits

Finally, we investigated whether *CREST* knockout affects the expression of other BAF subunits during motor neuron differentiation. We compared the mRNA levels of neuronal BAF subunits (*CREST*, *BAF40b, BAF45b*, *BAF53b*), which are expressed specifically in day 6 motor neurons, to their homologous non-neuronal subunits (e.g., *Ss18, BAF40a, BAF45a*, *BAF53a*), which are expressed in day 0 ES cells or day 4 neural progenitors. During neuronal differentiation, non-neuronal subunits are typically replaced by these neuronal homologues^23^. Our analysis revealed that the neuronal BAF subunit genes were significantly downregulated in day 6 KO cells compared to WT cells (*P* < 4.88 × 10^−3^; Suppl. Fig. 17a). Similarly, other neuronal subunits (BAF45c, BAF170) were also significantly downregulated (Suppl. Fig. 17b). In contrast, the overall expression of the homologous non-neuronal and core BAF subunits (BAF57, BAF200) remained unchanged. This indicates that CREST may contribute to the expression of other neuronal BAF subunits during motor neuron differentiation.

To validate these transcriptional changes at the protein level, we performed Western blotting for three neuronal (BAF53b, BAF170, BRM) and four non-neuronal (ARID1A, BRG1, BAF57, SS18) BAF subunits. Quantitative analysis showed a significant reduction in BAF53b protein levels upon *CREST* knockout (*P* = 3.92 × 10^−3^), whereas BAF170, BRM, and non-neuronal BAF subunits showed no significant change (Suppl. Fig. 18a). We then utilized co-immunoprecipitation to determine whether *CREST* knockout disrupted protein-protein interactions within the BAF complex. The interaction between BAF170 and BRM was unaltered by *CREST* knockout, and BAF53b did not directly interact with BRM in motor neurons (Suppl. Fig. 18b). These results suggest that while *CREST* knockout may downregulate the protein levels of specific neuronal BAF subunits, *CREST* knockout does not disrupt their overall structural assembly within motor neuron BAF complexes.

Taken together, our study reveals that CREST, a non-catalytic subunit of neuronal BAF chromatin remodeling complexes, plays a critical non-canonical role in motor neuron development. By recruiting CBP histone acetyltransferase and BRM ATPase, and by interacting with neuronal TFs, CREST activates mature neuronal enhancers. Therefore, CREST acts as an essential mediator between epigenetic regulators and cell-type-specific enhancers to ensure proper motor neuron function.

## Discussion

### Non-catalytic functions of neuronal BAF complexes during motor neuron development

While numerous studies have examined the importance of catalytic ATPases in chromatin remodeling, the roles of non-catalytic subunits in gene expression remain largely elusive. Here, we reveal that the non-catalytic BAF subunit CREST activates mature neuronal enhancers during motor neuron development. Overexpression studies previously showed CREST interacts with CBP histone acetyltransferase via its C-terminal domain^78^. In patients with ALS, mutations in the *CREST* gene frequently occur within this C-terminal domain, rendering the CBP-interacting region non-functional^79^. This suggests the CBP-interacting domain is crucial for proper H3K27ac deposition in healthy motor neurons. Indeed, our data demonstrate that CREST specifically co-localizes with CBP, depositing H3K27ac to activate target genes in mature motor neurons. Conversely, *CREST* knockout disrupts CBP recruitment and abolishes this acetylation. Furthermore, while the related acetyltransferase p300 also deposits H3K27ac^80,81^, we found that its occupancy is not specific to CREST-bound enhancers. Thus, our study elucidates how a non-catalytic BAF subunit recognizes and activates specific neuronal enhancers via protein-protein interactions with distinct co-activators.

### Characterization of transcriptional and epigenetic features at CREST-bound enhancers

**Fig. 7:**
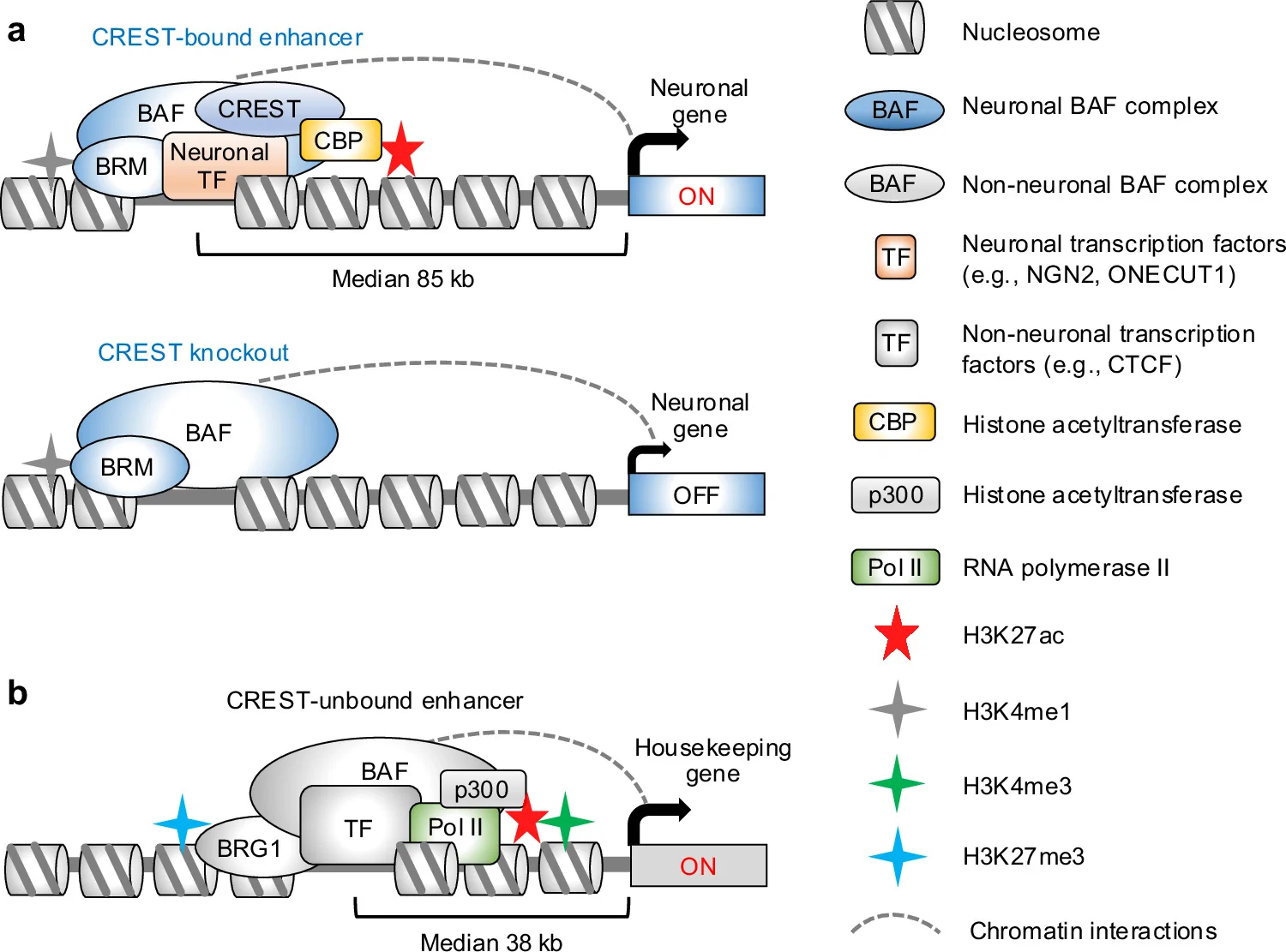
Model of CREST-dependent regulation of neuronal enhancers controlling the gene expression program for motor neuron maturation. **a** We propose that CREST- and BRM-containing BAF complexes are recruited to neuronal enhancers through interactions with neuronal TFs, such as ONECUT1 and NGN2, in motor neurons. Accessibility of CREST-bound enhancers is regulated by the neuron-specific ATPase, BRM, whose chromatin regulation is independent of CREST binding at enhancers. Then, CREST recruits CBP histone acetyltransferase, not p300, to neuronal enhancers for acetylation of histone H3K27. CREST-bound enhancers associate with promoters of mature neuronal genes through long-range chromatin interactions. CREST-bound enhancers were significantly distant from the TSSs of their target neuronal genes (Suppl. Fig. 6a; the median distance between a CREST-bound enhancer and the closest TSS: 84.67 kb), with long intergenic or genic DNA regions. In the absence of CREST, neuronal enhancers are bound by BRM-containing BAF complexes but not activated by CBP. **b** CREST-unbound enhancers are occupied by non-neuronal TFs, become accessible through BRG1 ATPase, and are activated by p300 histone acetyltransferase. CREST-unbound enhancers are epigenetically bivalent, having both active (H3K27ac, H3K4me3) and repressive chromatin marks (H3K27me3), and transcriptionally active via RNA polymerase II. CREST-unbound enhancers are associated with non-neuronal genes, such as housekeeping genes, and are closer to the TSSs of their target genes (median distance from the closest TSS: 38.27 kb) compared with CREST-bound enhancers.

We propose a model where CREST and other neuronal BAF subunits, such as the BRM ATPase, co-occupy mature neuronal enhancers to regulate chromatin accessibility and epigenetic states (Fig. 7a). BRM binds to these enhancers independently of CREST to establish accessibility. Our ChIP-seq data suggest these complexes may be recruited by neuron-specific TFs, such as ONECUT1 and NGN2 (Fig. 5g, h). Once localized, CREST recruits CBP to acetylate H3K27, activating the enhancer to drive mature neuronal gene transcription via long-range chromatin interactions. Notably, the primed enhancer mark H3K4me1 is present regardless of CREST binding, potentially acting as an initial recruitment signal for BAF complexes^82,83^.

In contrast, enhancers of housekeeping genes lack CREST-containing complexes (Fig. 7b). These CREST-unbound enhancers are occupied by non-neuronal BAF complexes, the ubiquitous TF CTCF, and the p300 co-activator. They exhibit bivalent epigenetic regulation, featuring both active (H3K27ac, H3K4me3) and repressive (H3K27me3) marks. Furthermore, these distinct enhancers exhibit divergent transcriptional states. Unlike non-neuronal enhancers, which are enriched for transcriptional marks, such as H3K4me3 and paused RNA polymerase II (associated with enhancer RNA)^70,84,85^, CREST-bound neuronal enhancers lack these marks. We propose classifying these non-neuronal BAF complexes as “transcriptional BAF (tBAF)” complexes, which maintain continuous transcription of generic cellular genes. Conversely, neuronal BAF complexes appear transcriptionally inactive at enhancers, likely mediating transient activation in response to specific neuronal signals. Additionally, CREST-bound enhancers are located significantly further from their target promoters than CREST-unbound enhancers (Suppl. Fig. 6a). This implies that neuronal genes require more distant regulatory elements to orchestrate the complex expression patterns of the CNS^77^.

### Context-dependent utilization of cell type-specific subunits of BAF complexes

BAF complexes utilize cell-type-specific subunits to form unique, multi-functional epigenetic regulators^14^. Subunits of these complexes are among the most frequently mutated chromatin regulators in neurological diseases^67^. To develop comprehensive therapeutic strategies for neurodegenerative diseases linked to CREST-containing BAF complexes, several critical questions remain. Future studies must determine exactly which neuronal TFs and DNA motifs are essential for recruiting these complexes, which specific CREST domains mediate TF interactions, whether neuronal BAF complexes maintain structural stability without CREST, and the precise physical mechanisms driving their long-distance chromatin interactions.

In this study, we established that CREST is critical for motor neuron maturation, actively accumulating distinct epigenetic marks at enhancers to drive the expression of synaptic and motor neuron-specific genes. However, CREST is also broadly expressed across other developing neuronal cell types^86^. Whether CREST is recruited to and activates enhancers in these other populations via different subtype-specific TFs remains an open question. Ultimately, our findings demonstrate that CREST-bound neuronal enhancers possess unique transcriptional and epigenetic features. Uncovering the molecular mechanisms governing chromatin remodeling and activation by CREST and other BAF subunits will significantly advance our understanding of cell-type-specific BAF functions in both the healthy and diseased central nervous system.

## Methods

### Experimental Methods

#### Cell culture and differentiation

The embryonic stem (ES) cell line was derived from a male embryo of the inbred mouse strain 129/Ola ES-E14. ES cells were cultured for two days on 0.1% gelatin-coated plates in ES growth medium consisting of EmbryoMax DMEM supplemented with 15% ES cell-grade fetal bovine serum, 2 mM L-glutamine, non-essential amino acids, nucleosides, 0.36 µM beta-mercaptoethanol, and 1000 U/mL leukemia inhibitory factor at 37 °C and 5% CO_2_. To initiate embryoid body formation, cells were trypsinized on day 0 and seeded at 8 × 10^5^ cells/mL in ADFNK medium, comprising Advanced DMEM/F12 and Neurobasal Medium at a 1:1 ratio, 10% Knockout Serum Replacement, 2 mM L-glutamine, and 0.4 µM beta-mercaptoethanol. Medium was changed on days 2 and 5. On day 2, 1 µM all-trans retinoic acid and 0.25 µM Smoothened agonist were added. On days 4 and 5, cells received 5 µM gamma-secretase inhibitor. Differentiated cells were collected on day 6 for downstream assays, including Western blotting, immunostaining, RNA-seq, ChIP-seq, ATAC-seq, and Hi-C. For day 9 neuronal cultures, embryoid bodies were collected on day 4 and plated on coverslips coated with 0.001% poly-L-ornithine and 1% Matrigel. Cells were maintained in a 1:1 mixture of Advanced DMEM/F12 and Neurobasal Medium supplemented with 2% B-27, horse serum, 2 mM L-glutamine, 0.1 mM beta-mercaptoethanol, and penicillin-streptomycin. Medium was replaced on days 6 and 8, and attached cells were harvested on day 9 for RNA-seq. Day 6 neuronal cultures were prepared similarly, except that coverslips were coated with 0.001% poly-L-ornithine and 4 µg/mL Matrigel.

#### Generation of the CREST mutant cell line

The 129/Ola ES-E14 line was used to generate homozygous and heterozygous CREST knockout cells by CRISPR/Cas9 editing. Guide RNAs (gRNAs) were designed to target both sides of CREST and generate a frameshift deletion. All guide RNA sequences listed in Supplementary Information were synthesized from Integrated DNA Technologies, Inc. (Coralville, IA, USA). Synthesized guide sequences were cloned into a U6 target gRNA expression plasmid (Addgene, Plasmid #41824). ES cells were seeded on mouse embryonic fibroblasts for 48 h and transiently nucleofected with the two gRNA vectors and a pCas9-mCherry plasmid containing Cas9 and the mCherry reporter. Nucleofection was performed using a P3 Primary Cell 4D-Nucleofector X Kit and Amaxa 4D-Nucleofector. After 40–48 h, cells were dissociated and selected by fluorescence-activated cell sorting based on mCherry expression. Approximately 2400 single cells were plated on mouse embryonic fibroblasts and cultured for 4–6 days to form colonies. Individual colonies were picked and genotyped by PCR, and genomic DNA from candidate knockout lines was Sanger sequenced to confirm the deletion. Clones containing homozygous frameshift deletions were expanded and frozen for subsequent motor neuron differentiation. Heterozygous mutant lines were generated using the same procedure, with heterozygous clones selected after genotyping.

#### Western blotting

Cell pellets were resuspended in lysis buffer containing 150 mM NaCl, 1% Triton X-100, 0.5% sodium deoxycholate, 0.1% sodium dodecyl sulfate, and 50 mM Tris-HCl (pH 8.0), supplemented with protease inhibitor. Samples were passed through 25-gauge needles, incubated on ice for 20 min, and centrifuged at 15,870 × g for 15 min at 4 °C. Laemmli sample buffer and beta-mercaptoethanol were added, and samples were heated at 95 °C for 5 min. Equivalent protein amounts were separated on 4–15% precast gels and transferred to 0.2 µm nitrocellulose membranes using a dry blotting system. Membranes were blocked for 2 h at 25 °C in PBS-T containing 6% bovine serum albumin (BSA), then incubated overnight at 4 °C with anti-CREST (Proteintech, 12439-1-AP; lot#, 00042079), anti-GAPDH (Cell Signaling, 2118; lot# 082020-14), anti-MAP2 (Cell Signaling, 4542S; lot# 032019-4), anti-SS18 (Cell Signaling, 21792; lot# 092029-1), anti-ChAT (Millipore Sigma, AB144P; lot# 4254299), anti-BAF170 (Cell Signaling, 12760; lot# 052030-2), anti-BAF53b (Antibodies Incor., 75-311; lot# 449-4AK-23), anti-BRM (Bethyl Lab, A301-015A; lot# 2), anti-ARID1A (Cell Signaling, 12354S; lot# 022025-4), anti-BRG1 (Abcam, ab110641; lot# 1141327-3), and anti-BAF57 (Bethyl Lab, A300810AT; lot#, 2) antibodies. After five PBS-T washes, membranes were incubated for 1 h at 25 °C with HRP-conjugated anti-rabbit IgG in 6% BSA/PBS-T, washed again, developed using a chemiluminescent substrate, and imaged. For quantification, target-protein band intensities were normalized to the corresponding GAPDH loading-control intensity within each biological replicate.

#### Immunofluorescence staining

Day 6 embryoid bodies were fixed in 4% paraformaldehyde in PBS for 5 min at 25 °C, embedded in O.C.T. compound, frozen, cryosectioned, and placed on glass slides. Sections were washed with PBS and blocked for 30 min at 25 °C in PBS containing 10% fetal bovine serum or donkey serum, 0.3% Triton X-100, and 0.05% sodium azide. After additional washes, sections were incubated overnight at 4 °C with anti-CREST (Proteintech, 12439-1-AP; lot#, 00042079), anti-HB9 (DSHB, 81.5C10), anti-ISL1 (Neuromics, GT15051), and anti-H3K27ac (Abcam, ab4729; lot# 1059037-6) antibodies in dilution buffer containing 2% serum, 0.1% Triton X-100, and 0.05% sodium azide. Sections were then washed and incubated for 1 h at 25 °C with Alexa Fluor-conjugated anti-goat, anti-mouse, and anti-rabbit secondary antibodies. Samples were treated with DAPI for 20 min, mounted in DAPI-containing medium, and stored at 4 °C. At least five embryoid bodies per time point or condition were randomly selected for quantification, with an average of 393 H3K27ac-positive cells per embryoid body. For day 6 neuronal cultures, attached embryoid bodies were washed with PBS and fixed in 4% paraformaldehyde for 20 min at room temperature. Cells were washed with 0.1% BSA in ice-cold PBS, permeabilized with 0.035% Triton X-100 for 15 min, and blocked for 30 min. Cells were incubated overnight at 4 °C with anti-MAP2 (Cell Signaling, 4542S; lot# 032019-4), anti-HB9 (DSHB, 81.5C10), and anti-TUBB3 (Sigma, T2200-200ul) antibodies. The next day, cells were washed, incubated with anti-rabbit secondary antibody for 1 h at room temperature, washed again, mounted with DAPI-containing medium, and dried overnight at 4 °C before imaging.

#### Alkaline phosphatase assay

Wild-type and CREST knockout ES cells were plated on 0.1% gelatin-coated plates in EmbryoMax DMEM supplemented with 10% ES cell-grade fetal bovine serum, 2 mM L-glutamine, 0.1 mM beta-mercaptoethanol, and leukemia inhibitory factor. After two days, alkaline phosphatase staining was performed using a commercial staining kit, and ES cell colonies were imaged at 10× magnification by light microscopy.

#### RNA-seq

Total RNA was extracted from differentiated cells using TRIzol reagent and treated with DNase to remove DNA contamination. Poly(A) mRNA was enriched using magnetic isolation, complementary DNA was synthesized, and directional RNA-seq libraries were prepared using the NEBNext Ultra II Directional RNA Library Prep Kit. Libraries were sequenced on Illumina platforms.

#### ChIP-seq

Approximately 30 million cells were collected per ChIP-seq experiment. Cells used for CREST and CBP ChIP-seq were double-crosslinked with disuccinimidyl glutarate for 30 min followed by 1% formaldehyde for 15 min; other samples were crosslinked with 1% formaldehyde alone. Cells were lysed, chromatin pellets were isolated and solubilized, and chromatin was fragmented by sonication. Protein G- or Protein A-coupled magnetic beads were used to immunoprecipitate chromatin with anti-CREST (ProteinTech, 12439-1-AP; lot#, 00042079), anti-CBP (Cell Signaling, 7389S), anti-p300 (Abcam, ab275378; lot# 1074860-31), anti-p300 (Sigma, 05-257), anti-TIP60 (ProteinTech, 10827-1-AP; lot # 00138365), anti-IgG (Cell Signaling, 2729; lot# 11), anti-BRM (Bethyl, A301-015A; lot# 2), anti-BRG1 (Abcam, ab110641; lot# 1000647-1), anti-H3K27ac (Abcam, ab4729; lot# GR3251519-2), anti-H3K4me1 (Abcam, ab8895; lot# GR3442591-2), anti-H3K4me3 (Abcam, ab8580; lot# GR3362386-3), anti-H3K27me3 (Abcam, ab6002), anti-CTCF (Millipore, 07-729; lot# 3515588), and anti-RPB1 (Cell Signaling, 2629; lot# 062025-4) antibodies. Fragmented chromatin and antibody-coated beads were incubated overnight at 4 °C. Immunoprecipitated chromatin was washed, eluted, and reverse-crosslinked; proteins and RNA were degraded, and DNA was purified. Libraries were prepared by end preparation, adaptor ligation, purification and size selection, ligation-mediated PCR amplification, and gel purification, followed by Illumina sequencing.

#### ATAC-seq

Approximately 50,000 cells were collected for each ATAC-seq sample. Cell pellets were resuspended in lysis buffer containing 10 mM NaCl, 3 mM MgCl2, 0.1% IGEPAL CA-630, and 10 mM Tris-HCl (pH 7.4). Lysed pellets were isolated and resuspended in tagmentation master mix containing Tn5 transposase, 0.5% digitonin, 10% Tween 20, and PBS, then incubated at 37 °C for 30 min. Tagmented DNA was purified, size-selected, and amplified by ligation-mediated PCR. Amplified libraries were purified with magnetic clean-up and size-selection beads and sequenced on Illumina platforms.

#### Hi-C

Two biological replicates each of day 6 CREST knockout and wild-type motor neurons (1 × 10^6^ cells) were crosslinked in 1% formaldehyde for 10 min at 25 °C. Cells were lysed in Tris-HCl, NaCl, and IGEPAL CA-630 and digested with MboI. Digested fragments were labeled with biotin-14-dCTP and proximally ligated with T4 DNA ligase. Crosslinks were reversed overnight at 68 °C using proteinase K, SDS, and NaCl. DNA was purified with AMPure XP beads and sonicated. Ligated fragments were captured with streptavidin magnetic beads and washed thoroughly. Hi-C libraries were prepared by end repair, removal of unligated ends, 3′ adenosine addition, indexed-adapter ligation, and PCR amplification. PCR cycle numbers were determined using library quantification, and libraries were deep-sequenced in paired-end mode.

#### Co-immunoprecipitation

Day 6 cells were subjected to nuclear extraction using a Nuclear Complex Co-IP kit. Nuclear extracts were quantified by BCA assay and used immediately or stored at −80 °C. For each Co-IP, 150 µg nuclear extract was incubated overnight at 4 °C with 5 µg anti-CREST or anti-BRM antibody; a no-antibody control contained 100 µg nuclear extract. Protein A-coupled magnetic beads were added for 1 h at 4 °C, washed to remove nonspecific proteins and DNA, and proteins were eluted in 4× Laemmli sample buffer for 10 min at 95 °C. Seventy-five percent of each Co-IP sample was separated by SDS-PAGE alongside 25 µg nuclear extract as a control and transferred to PVDF membranes by wet transfer. Membranes were blocked for 1 h in PBS-T containing 3% BSA and incubated overnight at 4 °C with anti-BRM (Bethyl Lab, A301-015A; lot# 2), anti-ONECUT1 (Santa Cruz, sc-376167; lot# J2417), anti-NGN2 (Cell Signaling, 13144; lot# 052030-3), anti-β-Actin (Santa Cruz, sc-47778; lot# H1420), anti-BAF170 (Cell Signaling, 12760; lot# 052030-2), anti-BAF53b (Antibodies Incor., 75-311; lot# 449-4AK-23), and anti-GAPDH (Cell Signaling, 2118; lot# 082020-14) antibodies. Membranes were then incubated for 1 h with HRP-conjugated secondary antibodies, developed by chemiluminescence, and imaged.

### Data Analyses

#### RNA-seq analysis

Raw RNA-seq FASTQ files were mapped to the mouse genome (mm10) and reference gene annotation (M25, GRCm38.p6) using Salmon to estimate transcript read counts. Differential expression was analyzed with DESeq2. For transcript abundance estimates, raw datasets were also aligned to mm10 using RNA-STAR, and FPKM values were calculated for each gene and sample using Cufflinks. A value of 0.1 was added to each gene to avoid zero denominators on logarithmic scales, after which median-normalized FPKM values were log_2_-transformed.

#### Genomic mapping analysis

ChIP-seq and ATAC-seq reads were processed by removing low-quality reads and adapter sequences with Trim Galore v0.6.7, followed by alignment to mm10/GRCm38 using Bowtie2. BAM files were converted to single-read BED format with bedtools and to BIGWIG format for signal visualization in Integrative Genomics Viewer. Peaks were called with MACS2 using IgG or no-antibody ChIP-seq data as negative controls. Day 6 wild-type CREST ChIP-seq replicates yielded 9719 and 8620 peaks, while wild-type ATAC-seq replicates yielded 17,080 and 23,165 highly accessible regions. High-confidence CREST peaks were identified by differential binding analysis with DiffBind, comparing two wild-type CREST replicates with two CREST knockout replicates at *P* < 0.05. Peaks with midpoints within 2.0 kb were merged, retaining the more significant peak, to define CREST-bound regions. Regions within 3 kb of the nearest transcription start site (TSS) were classified as promoters; regions farther than 3 kb were classified as distal cis-regulatory regions. Analyses focused on highly accessible DNA regions. Among 14,373 highly accessible distal regions identified on day 6, 4221 were classified as CREST-bound by overlap with CREST peaks within 2.0 kb, and 10,152 were classified as CREST-unbound.

#### Hi-C analysis

Hi-C reads were mapped to mm10 using BWA-MEM. Chimeric reads, reads with MAPQ < 10, and putative self-ligated reads with genomic distances < 5 kb were removed, and potential PCR duplicates were marked with Picard MarkDuplicates. Hi-C contact matrices were Knight-Ruiz normalized, converted to .hic format, and subjected to bin-wise quantile normalization to reduce between-sample variation. Directionality index scores were calculated from normalized 40 kb-resolution contact maps using Domain Caller within a 1 Mb window. To identify altered contacts between differentially expressed genes and CREST-bound accessible regions, normalized 40-kb bin pairs connecting gene TSSs and CREST-bound accessible-region summits within 2 Mb were collected for day 6 wild-type and CREST knockout samples. Contacts were analyzed with LIMMA and considered differential when the normalized Hi-C contact difference exceeded 100 and *P* < 0.01. Genome-wide long-range gene-to-enhancer relationships were further modeled using the activity-by-contact (ABC) framework, which combines cis-regulatory activity with chromatin contact frequency. ABC scores were calculated at 10 kb resolution for cis-regulatory element-gene pairs within 2 Mb. Cis-regulatory activities were quantile-normalized, analysis was restricted to expressed genes, and promoter-centered connections with ABC scores >0.1 were considered significant.

#### Gene ontology analysis

Gene ontology (GO) enrichment was assessed for CREST-bound and CREST-unbound regions using GOrilla. REViGO was used with a 0.7 SimRel similarity threshold based on Resnik and Lin similarity measures to generate summarized, non-redundant biological-process terms. For ChIP-seq and ATAC-seq analyses, intragenic peaks were assigned to the nearest TSS among mm10 mRNA genes, and all annotated protein-coding genes were used as the background set. For RNA-seq GO analyses, all expressed mRNA genes were used as the background.

#### Box plot analysis

For box-and-whisker analyses of log2 FPKM relative to median values, data were restricted to mRNA genes significantly downregulated or upregulated (*P* < 0.05) in day 6 CREST knockout cells compared with day 6 wild-type mature motor neurons; non-differentially expressed and non-expressed genes were excluded. Boxes represent the first and third quartiles, the internal line indicates the median, and whiskers extend to 1.5 times the interquartile range.

#### Statistical analyses

For paired comparisons of RNA expression expressed as log_2_(FPKM + 0.1) between wild-type and knockout cells, P values were calculated using two-tailed paired t-tests. Two-tailed unpaired Student’s t-tests assuming equal variance were used for comparisons of RNA-expression fold change, FPKM values, distances between distal regions and TSSs, and ChIP-seq signal intensities between datasets. Differences in gene expression for rank-based comparisons were evaluated using two-sided Wilcoxon rank-sum tests. For comparisons of all possible pairs of means while controlling the experiment-wise error rate, one-way analysis of variance (ANOVA) followed by Tukey’s Honestly Significant Difference post hoc test was used.

### Reporting summary

Further information on research design is available in the Nature Portfolio Reporting Summary linked to this article.

## Supplementary information

Supplementary Information

Transparent Peer Review file

Reporting Summary

Description of Additional Supplementary Files

Supplementary Dataset 1

Supplementary Dataset 2

Supplementary Dataset 3

Supplementary Dataset 4

Supplementary Dataset 5

Supplementary Dataset 6

Supplementary Dataset 7

Supplementary Dataset 8

Supplementary Dataset 9

Supplementary Dataset 10

Supplementary Dataset 11

## Data Availability

The raw and processed sequencing data generated in this study have been deposited in the National Center for Biotechnology Information (NCBI) Gene Expression Omnibus (GEO) under the accession codes GSE234503, GSE291506, and GSE326223 for RNA-seq, GSE291240, GSE291396, and GSE326333 for ChIP-seq, GSE291510 for ATAC-seq, and GSE281024 for Hi-C. We also used publicly available data in the NCBI GEO under the accession codes GSE79561 for RNA-seq, ChIP-seq, and ATAC-seq in this study. All other relevant data generated in this study are provided in the Supplementary Data files.
