# Supplementary material for: CREST functions as a molecular bridge between neuron-specific enhancers and epigenetic regulators in mature motor neurons
Source: Nat Commun. 2026 Oct 10;17:11520. doi: 10.1038/s41467-026-78396-x (PMC13745572; doi:10.1038/s41467-026-78396-x)
Supplement: Supplementary file 1 [file 41467_2026_78396_MOESM1_ESM.pdf]

CREST functions as a molecular bridge between neuron-specific enhancers and epigenetic regulators in mature motor neurons

Supplementary Information

SUPPLEMENTARY FIGURES

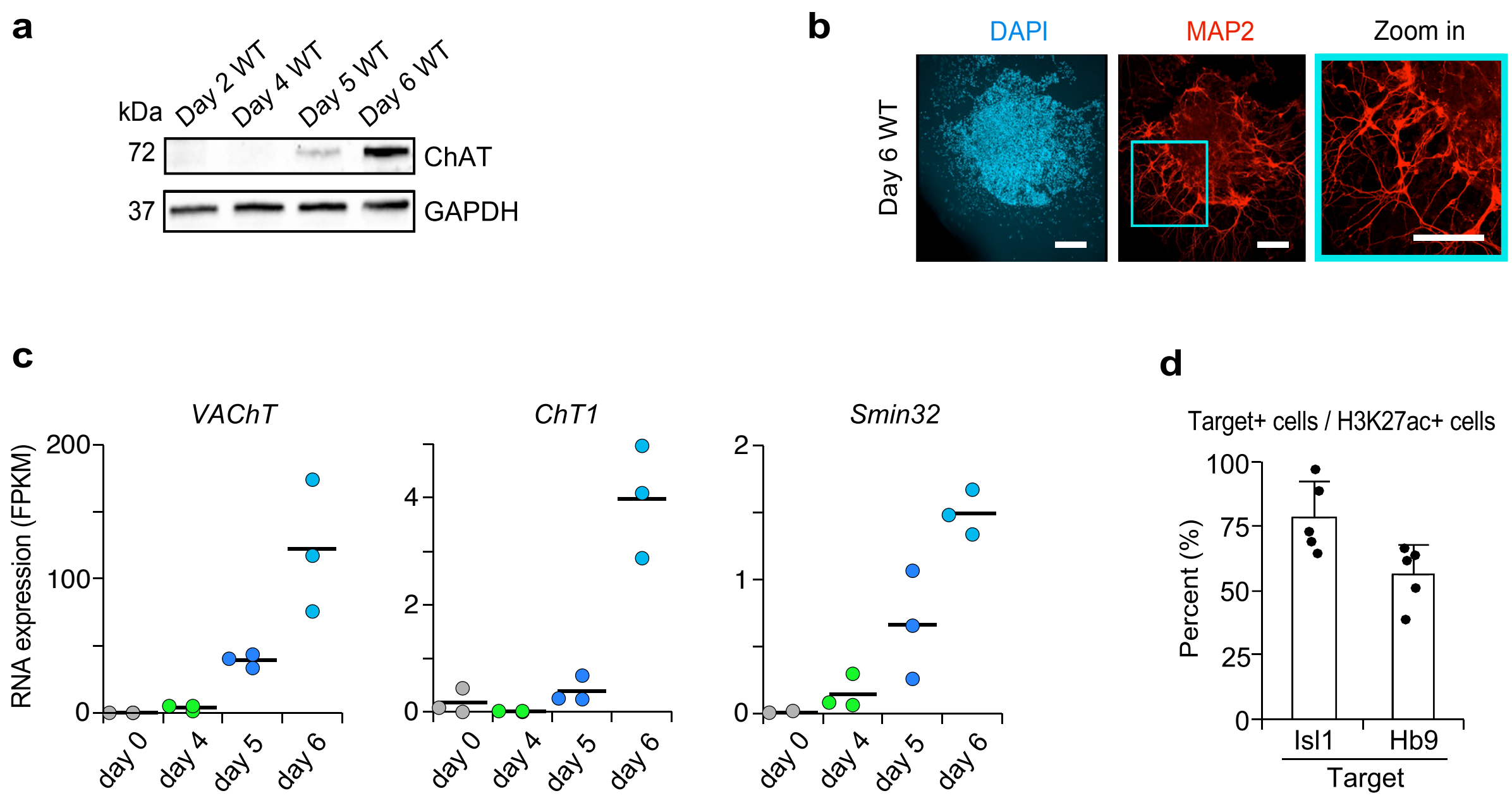

Supplementary Fig. 1 | **Mouse ES cell-derived mature motor neurons exhibit neuronal dendritic outgrowth.**

a, Western blot confirmed that the cholinergic neuronal marker ChAT is expressed at ~72 kDa (kilodaltons) only in day 6 wild-type (WT) motor neurons during the differentiation. A housekeeping protein GAPDH (~37 kDa) was used as a loading control (Supplementary Data 3).

b, Immunostaining of differentiated embryoid bodies on laminin-coated surfaces showing the nuclear-specific dye DAPI (4',6-diamidino-2-phenylindole) and the mature neuronal marker MAP2 (microtubule-associated protein 2) in day 6 motor neurons. Scale bars represent 50 µm.

c, RNA expression (FPKM) of three genes encoding motor neuron markers, *VAChT, ChT1*, and *Smin32*, measured by RNA-seq during motor neuron differentiation (day 0 ES cells, day 4 neural progenitors, day 5 nascent motor neurons, and day 6 WT mature motor neurons) from three biological replicates (*n* = 3 independent experiments) of independent cell differentiations. Horizontal lines in the dot plots show the average.

d, Quantification of transcriptionally active (H3K27ac-positive) day 6 WT cells expressing the motor neuron-specific markers ISL1 or HB9. Embryoid bodies were immunostained for HB9, ISL1, and H3K27ac, followed by the quantification of immuno-positive cells. Data are presented as mean ± SEM (*n* = 5 embryoid bodies per target marker).

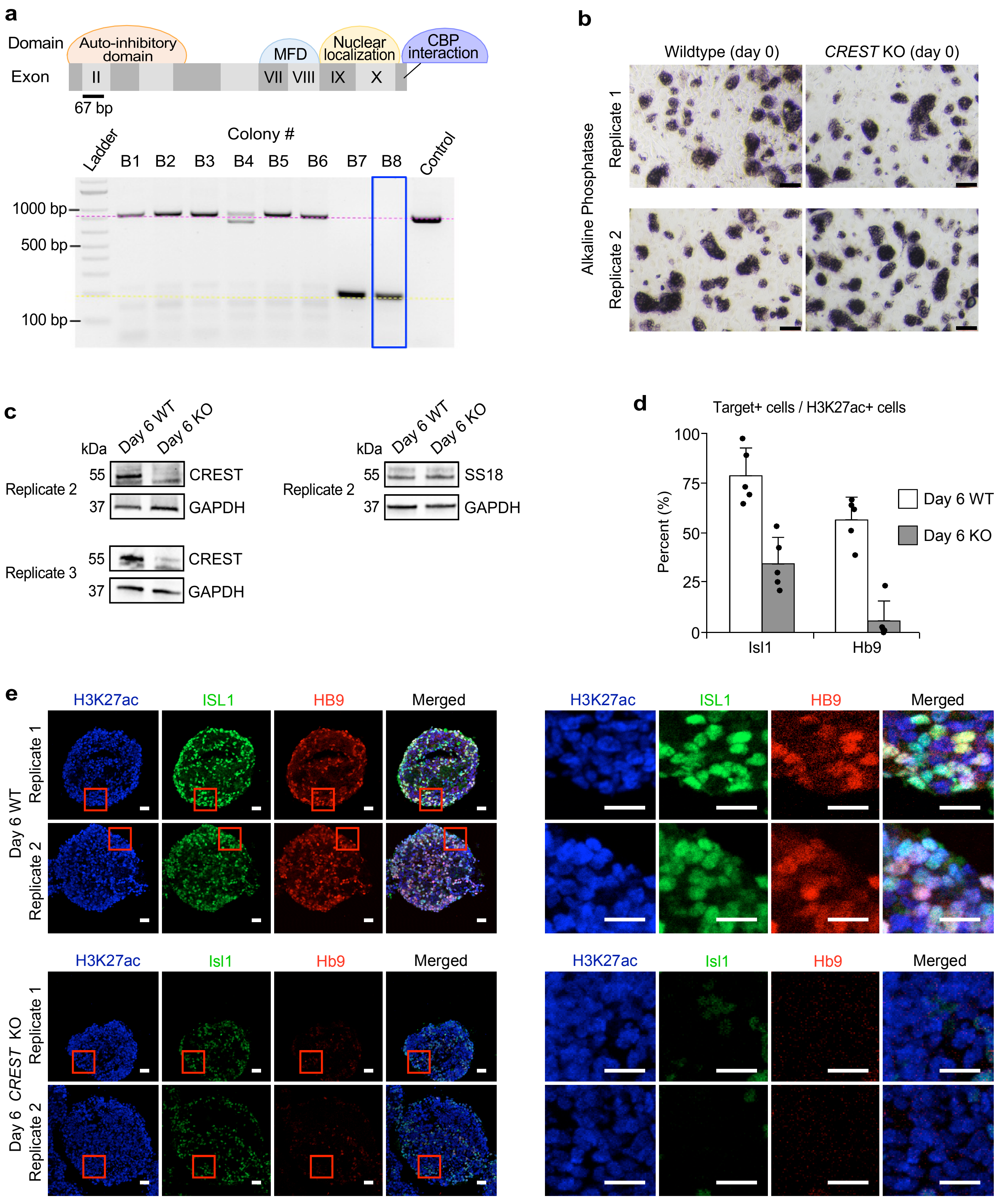

Supplementary Fig. 2 | **Generation of a mouse ES cell line containing the *CREST* knockout mutation.**

a, *Upper:* Protein domain structure of *CREST*, which contains 11 exons with four known protein domains: auto-inhibitory domain, multi-functional domain (MFD), nuclear localization domain, and CREB-binding protein (CBP) interaction domain. To generate the frameshift knockout (KO) mutation of *CREST*, 67 bp from exon 2 and 643 bp from the 5' intron were deleted using a CRISPR/Cas9 genome editing system. *Lower:* Genotyping of 8 ES cell colonies, generated via CRISPR/Cas9, analyzed on a 2% agarose gel. A 710 bp deletion (67 + 643 bp) was confirmed in the homozygous KO colonies B7 and B8 (*CREST* $^{-/-}$). The B8 ES cell colony was used for the differentiation and downstream analyses in this study. The control lane represents the genotype of a WT colony.

b, Day 0 ES cells were stained for the pluripotency marker alkaline phosphatase. Both WT and *CREST* KO ES cells exhibited similar levels of purple staining, indicating comparable ALP activity. Scale bars represent 200 µm.

c, Western blot confirmed that the *CREST* KO, generated via CRISPR/Cas9, causes a KO in CREST protein levels in day 6 differentiated cells. CREST is expressed at ~55 kDa (upper band) in day 6 WT cells, but not in day 6 KO cells. The CREST paralog, SS18, is expressed at ~55 kDa (lower band) in both day 6 WT and KO cells. The second and third biological replicates are shown here. Replicate 1 was shown in **Fig. 1d** (Supplementary Data 3).

d, Quantification of transcriptionally active (H3K27ac-positive) cells expressing the motor neuron-specific markers ISL1 or HB9 in day 6 WT and day 6 KO cells. Embryoid bodies were immunostained for HB9, ISL1, and H3K27ac, followed by the quantification of immuno-positive cells. Data are presented as mean ± SEM (n = 5 embryoid bodies per target marker).

e, Immunostaining of differentiated embryoid bodies showing H3K27ac, ISL1, and HB9 in day 6 WT motor neurons and day 6 KO cells. Red boxes in the left panels indicate the regions at higher magnification on the right. Scale bars represent 20 µm.

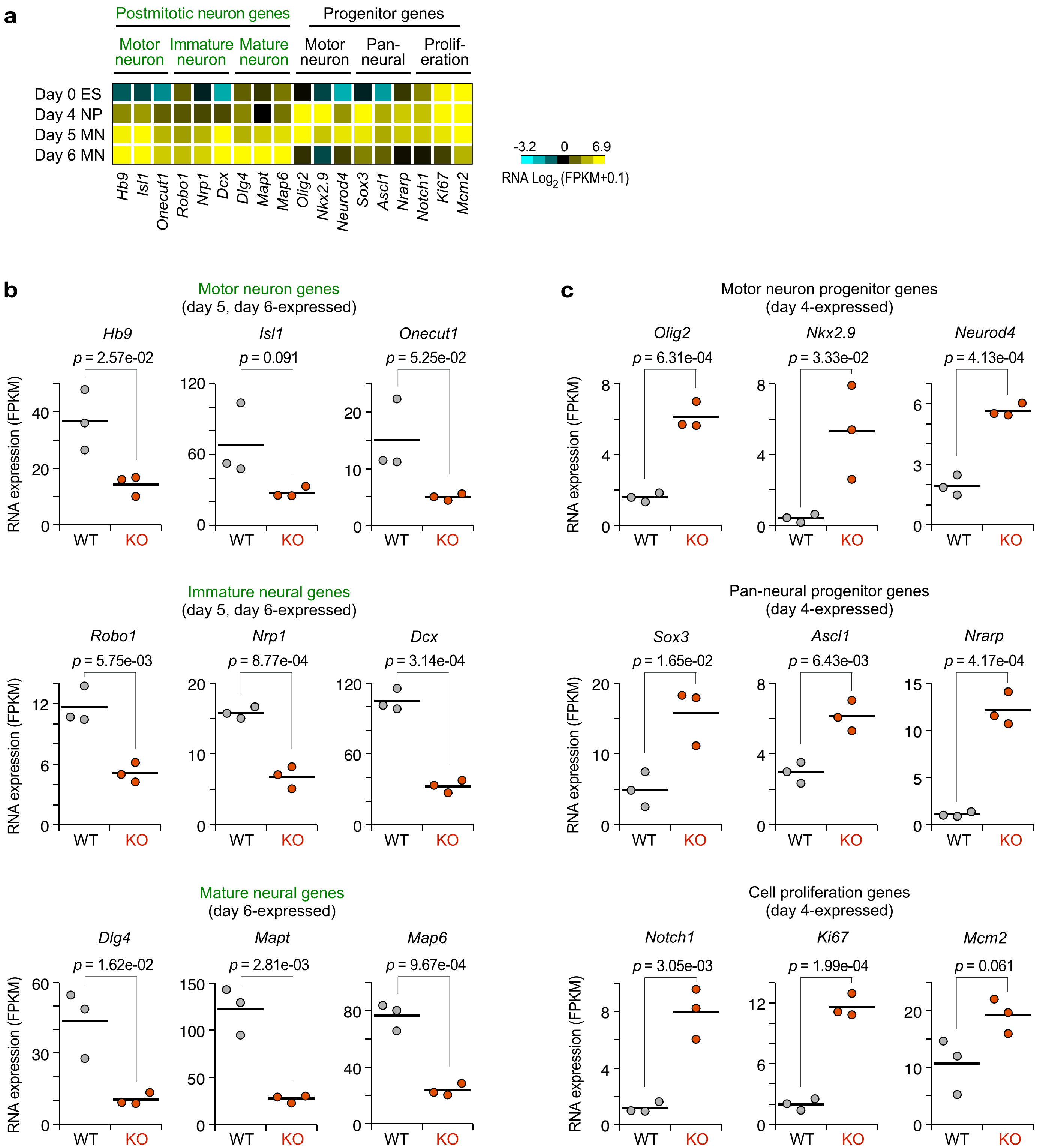

Supplementary Fig. 3 | Expression of motor neuron genes, immature, and mature neural genes downregulated by *CREST* knockout.

a, Heatmap of RNA expression ($\log_2$ FPKM+0.1) of selected genes encoding known markers of motor neurons (*Hb9, Isl1*, and *Onecut1*), immature neurons or developing axon guidance (*Robo1, Nrp1*, and *Dcx*), mature neurons (*Dlg4, Mapt*, and *Map6*), motor neuron progenitors (*Olig2, Nkx2.9*, and *Neurod4*), pan-neural progenitors or neural precursor (*Sox3, Ascl1*, and *Nrarp*), and cell proliferation (*Notch1, Ki67*, and *Mcm2*) during motor neuron differentiation; day 0 embryonic stem cell (ES), day 4 neural progenitor (NP), day 5 nascent and day 6 mature motor neuron (MN). FPKM values had 0.1 added to avoid producing a zero value, were median-normalized, and then $\log_2$-transformed (Supplementary Data 4). FPKM values represent averages from three biological replicates of independent motor neuron differentiations, shown in **Fig. 2a**.

b, c, RNA expression (FPKM) of selected neural genes (**b**) and progenitor genes (**c**), described in panel (**a**), was measured by RNA-seq in day 6 WT mature motor neurons and day 6 KO cells. FPKM values represent averages from three biological replicates on day 6 in WT and KO conditions. Horizontal lines in the dot plots show the average. *P*-values were calculated by a two-sided, two-sample *t*-test.

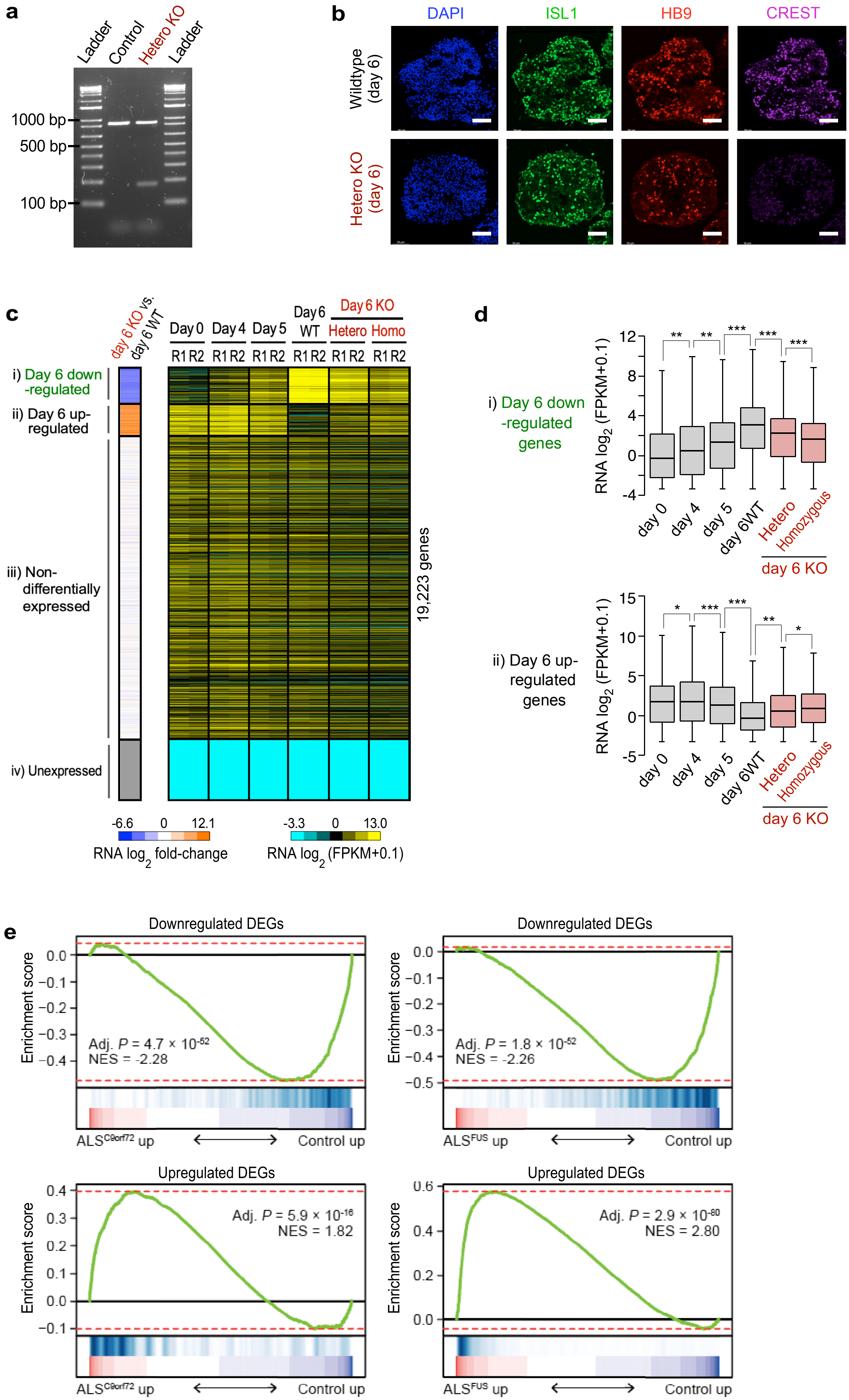
a
Ladder
Control
Hetero KO
Ladder
1000 bp
500 bp
100 bp
b
DAPI
ISL1
HB9
CREST
Wildtype (day 6)
Hetero KO (day 6)
c
day 6 KO vs. day 6 WT
Day 0
Day 4
Day 5
Day 6 WT
Day 6 KO
Hetero
Homo
R1 R2
i) Day 6 down -regulated
ii) Day 6 up-regulated
iii) Non-differentially expressed
iv) Unexpressed
19,223 genes
-6.6 0 12.1
RNA log2 fold-change
-3.3 0 13.0
RNA log2 (FPKM+0.1)
d
i) Day 6 down -regulated genes
ii) Day 6 up-regulated genes
RNA log2 (FPKM+0.1)
day 0
day 4
day 5
day 6WT
Hetero
Homozygous
day 6 KO
e
Downregulated DEGs
Upregulated DEGs
Enrichment score
Adj. P = 4.7 × 10-52
NES = -2.28
Adj. P = 1.8 × 10-52
NES = -2.26
Adj. P = 5.9 × 10-16
NES = 1.82
Adj. P = 2.9 × 10-80
NES = 2.80
ALSC9orf72 up
ALSFUS up
Control up

Supplementary Fig. 4 | The heterozygous *CREST* knockout mutation drives gene expression changes similar to those of the homozygous *CREST* knockout mutation.

a, Genotyping of a heterozygous *CREST* KO ES cell colony (*CREST* $^{+/-}$), generated via CRISPR/Cas9, analyzed on a 2% agarose gel. A 710 bp deletion (67 + 643 bp) was confirmed only in one allele, while the other allele remained intact. The control lane represents the genotype of a WT colony (Supplementary Data 3).

b, Immunostaining of differentiated embryoid bodies showing the nuclear-specific dye DAPI, the motor neuron-specific markers ISL1 and HB9, and CREST in day 6 WT motor neurons and day 6 heterozygous KO cells (*CREST* $^{+/-}$). Scale bars represent 50 µm.

c, *Left*: Heatmap of RNA expression ($\log_2$ fold-changes) of all 19,223 mRNA genes, ordered as displayed in **Fig. 2a**, in day 6 KO cells (*CREST* $^{-/-}$) compared with day 6 WT motor neurons. The four gene groups were defined in F**ig. 2a**. *Right*: Heatmap of RNA expression ($\log_2$ FPKM+0.1) of all mRNA genes in days 0, 4, 5, and 6 WT cells, day 6 heterozygous mutant cells (*CREST* $^{+/-}$), and day 6 homozygous mutant cells (*CREST* $^{-/-}$). FPKM values were adjusted by adding 0.1, median-normalized, $\log_2$-transformed, and ordered as displayed on the *left*. The heatmap includes data from two biological replicates (R1 and R2) derived from independent cell differentiations.

d, Box plots showing RNA expression ($\log_2$ FPKM+0.1) for 1614 downregulated genes (Group *i*) and 1429 upregulated genes (Group *ii*) shown in panel (**c**). FPKM values represent averages from two biological replicates, shown in panel (**c**). The plots display the median (line within boxes), first-to-third quartiles (bottom and top of boxes, respectively), and 1.5x the interquartile range (whiskers). ***$P < 2.51 \times 10^{-96}$; **$P < 5.83 \times 10^{-37}$; *$P < 9.23 \times 10^{-9}$; a two-sided paired *t*-test.

e, Analysis of day 6 *CREST* KO differentially expressed genes (DEGs) in mRNA-seq data from human iPSC-derived motor neurons with ALS mutations.[55] Gene set enrichment analysis (GSEA) plots showing down- and up-regulated genes in day 6 KO cells in the context of ALSC9orf72 mutations (left) and ALSFUS mutations (right) compared with control motor neurons.

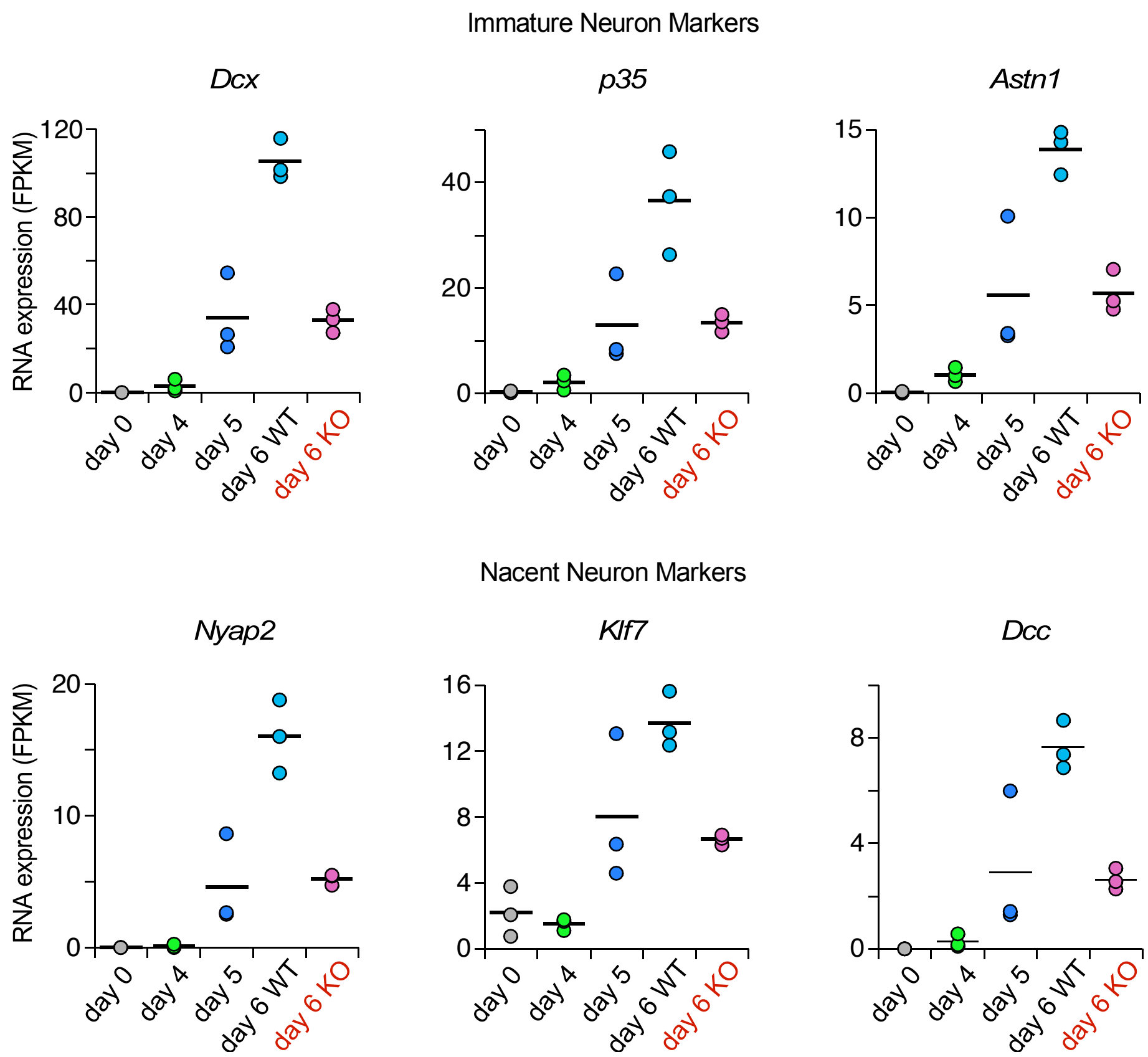

Supplementary Fig. 5 | The expression of key marker genes for immature and nascent neurons was similar between day 6 KO and day 5 WT cells.

RNA expression (FPKM) of key marker genes for immature neurons (*Dcx, p35,* and *Astn1*) and nascent neurons or developing axon guidance (*Nyap2, Klf7,* and *Dcc*), measured by RNA-seq during motor neuron differentiation (day 0 ES cells, day 4 neural progenitors, day 5 nascent motor neurons, day 6 WT mature motor neurons, and day 6 KO cells) from three biological replicates (*n* = 3 independent experiments) of independent cell differentiations. Horizontal lines in the dot plots show the average.

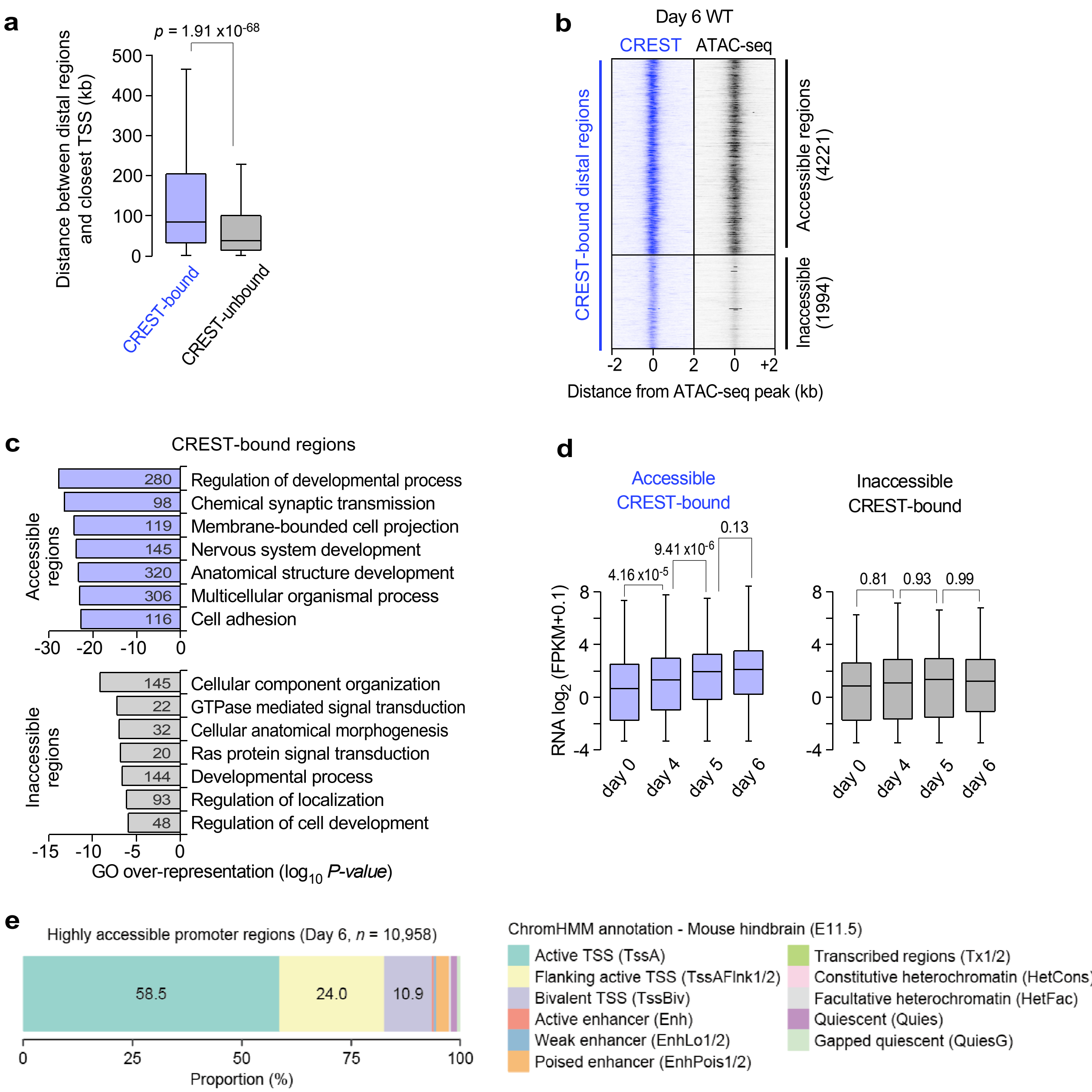

Supplementary Fig. 6 | **CREST mainly binds to accessible distal *cis*-regulatory regions of mature neuronal genes.**

a, Box plots of the distance between distal *cis*-regulatory regions and the closest transcription start site (TSS) for 6215 CREST-bound and 10,152 CREST-unbound distal regions are shown in **Fig. 3** (Supplementary Data 8). The box plots display the median (line within boxes), first-to-third quartiles (bottom and top of boxes, respectively), and 1.5x the interquartile range (whiskers). *P*-values were calculated by a two-sided, two-sample Student's *t*-test.

b, Genomic mapping of ChIP-seq CREST and ATAC-seq in day 6 WT motor neurons. 6215 CREST-bound distal *cis*-regulatory regions, defined in **Fig. 3a**, were grouped into accessible regions (n = 4221 regions), overlapped with ATAC-seq peaks, and inaccessible regions (n = 1994 regions) in day 6 WT motor neurons. All data were plotted relative to the ATAC-seq peak midpoints, except for inaccessible regions.

c, GO analysis of the 1082 genes associated with CREST-bound accessible distal regions (same as shown in **Fig. 3c**) and the 392 genes associated with CREST-bound inaccessible distal regions shown in panel (**b**). The seven most over-represented and non-redundant GO terms are displayed. Numbers within bars represent the count of genes associated with each GO term.

d, Box plots showing RNA expression (log_2 FPKM+0.1) for 1082 genes linked to accessible CREST-bound regions and 392 genes linked to inaccessible CREST-bound regions, identified in panel (**b**). FPKM values represent the average of three

biological replicates (detailed in **Fig. 2a**). Boxes display the first and third quartiles with the median indicated by the center line; whiskers extend to 1.5x the interquartile range. *P*-values shown above the whiskers were determined using a one-way analysis of variance (ANOVA) followed by Tukey's post hoc tests. Specific ANOVA results are as follows: Accessible group, $F(3,8) = 52.6$, $P = 2.28 \times 10^{-33}$; and inaccessible group, $F(3,8) = 1.29$, $P = 0.78$. Complete statistical values are provided in Supplementary Data 10.

e, A stacked bar plot showing the proportion of accessible promoter regions at day 6 with respect to 15-state chromHMM annotations derived from the E11.5 mouse hindbrain. CREST-bound distal regions were assigned to the closest TSS of these annotated known gene promoters.

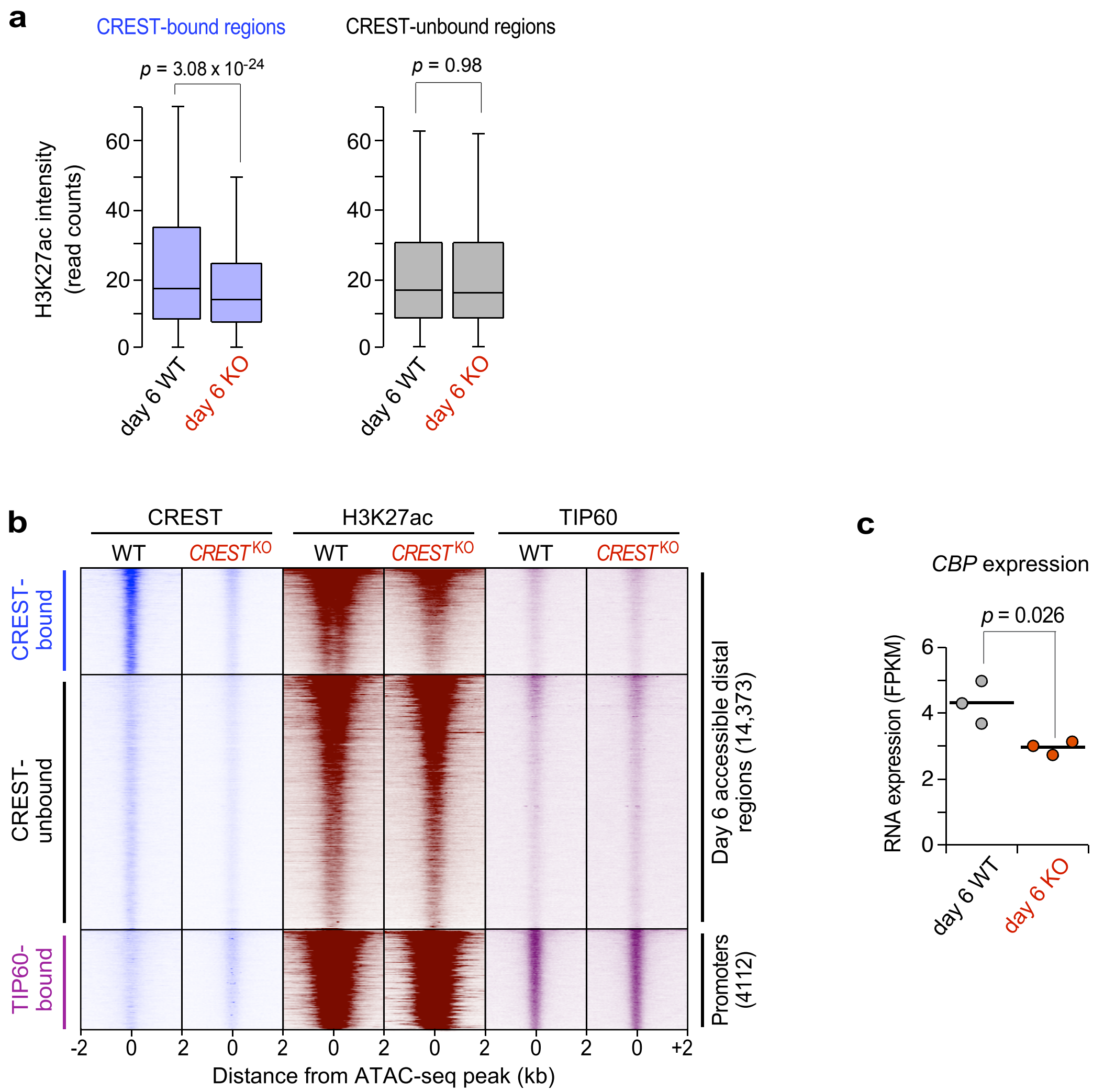

Supplementary Fig. 7 | **The ChIP-seq signals of H3K27ac in CREST-bound distal regions were significantly decreased following *CREST* knockout.**

a, Box plots of H3K27ac ChIP-seq intensity (read counts) in day 6 WT motor neurons and day 6 KO cells at CREST-bound distal regions (n = 4221 regions; left) and CREST-unbound distal regions (n = 10,152 regions; right), shown in **Fig. 3d**. The box plots display the median (line within boxes), first-to-third quartiles (bottom and top of boxes, respectively), and 1.5x the interquartile range (whiskers). *P*-values were calculated by a two-sided, two-sample Student's *t*-test.

b, ChIP-seq intensity of CREST, H3K27ac, and Tip60 in day 6 WT motor neurons at 14,373 accessible distal regions and 4112 promoter regions, bound by Tip60. All data were plotted relative to the ATAC-seq midpoints (shown in **Fig. 3b**), sorted, and ordered by ATAC-seq intensity, except TIP60-bound regions.

c, RNA expression (FPKM) of the *CBP* gene was measured by RNA-seq in day 6 WT mature motor neurons and day 6 KO cells. FPKM values represent averages from three biological replicates on day 6 in WT and KO conditions (Supplementary Data 4). Horizontal lines in the dot plots show the average. *P*-values were calculated by a two-sided, two-sample Student's *t*-test.

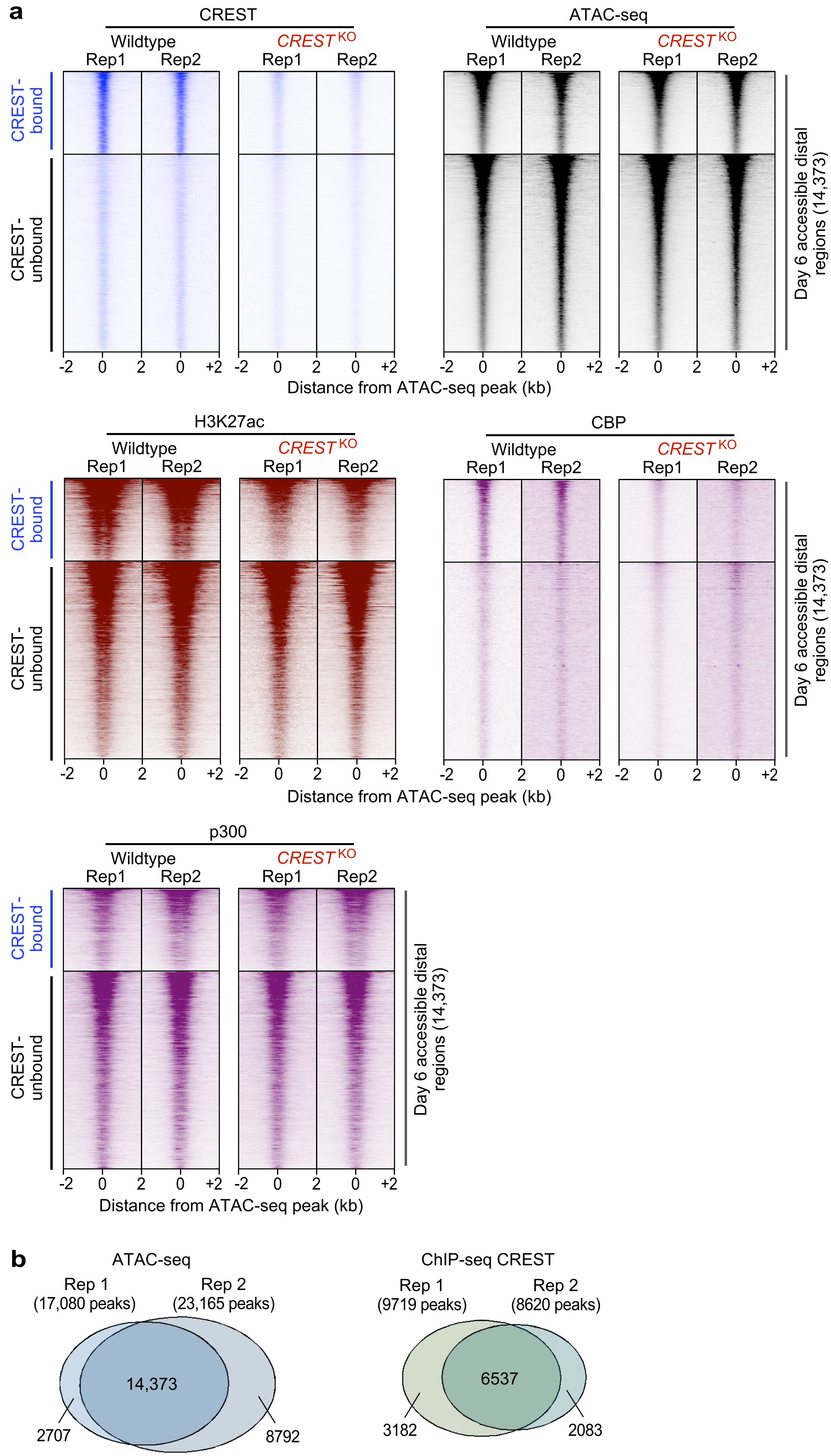

Supplementary Fig. 8 | ChIP-seq results in Fig. 3 are reproducible with independent motor neuron differentiations.

a, ChIP-seq mapping of CREST, H3K27ac, CBP, and p300, as well as ATAC-seq mapping at 4221 CREST-bound and 10,152 CREST-unbound distal *cis*-regulatory regions in day 6 WT motor neurons and day 6 KO cells, shown in **Fig. 3b***,* **d**, with two biological replicates of independent differentiations. All data were plotted relative to the ATAC-seq peak midpoints, sorted, and ordered by ATAC-seq intensity in day 6 WT motor neurons.

b, Venn diagram showing the overlap between two biological replicates of day 6 ATAC-seq and day 6 CREST ChIP-seq peak calls. These overlapped peaks were used for the subsequent analyses.

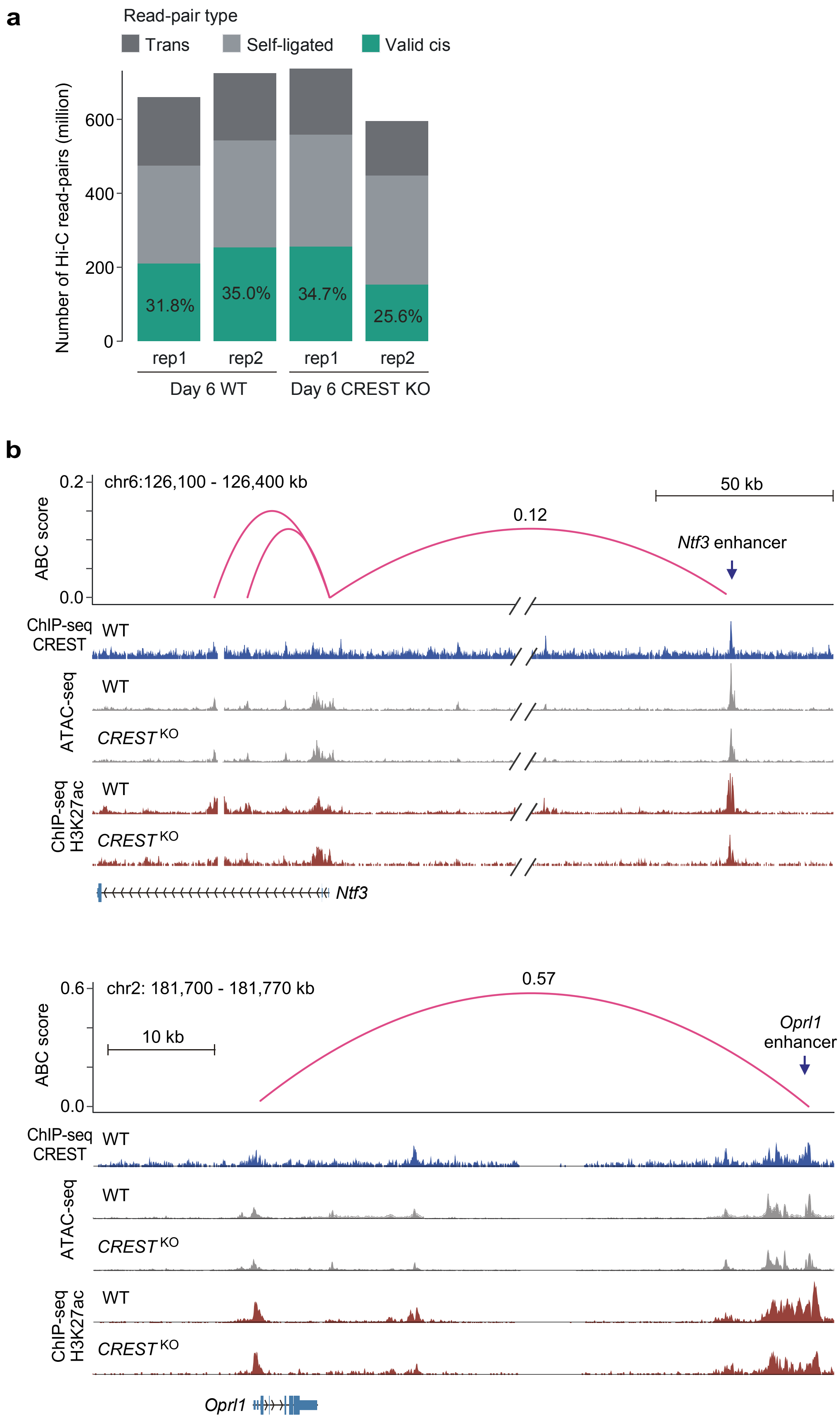

Supplementary Fig. 9 | CREST-bound distal regions activate neuronal genes through long-range chromatin interactions

a, Bar plots showing the number of sequenced read-pairs from *in situ* Hi-C libraries derived from day 6 WT motor neurons and day 6 KO cells using two biological replicates (rep) of independent differentiations. The Hi-C read-pairs were

categorized into trans (a pair of reads from different chromosomes), self-ligated (< 5 kb), and valid *cis* read-pairs (a pair of reads from the same chromosome).

b, The locations of CREST-bound distal regions, detected by ChIP-seq CREST, and H3K27ac, as well as ATAC-seq, for *Ntf3* (upper) and *Oprl1* (lower) genes in day 6 WT motor neurons and day 6 KO cells. The CREST-bound distal regions are linked to their target genes by a significant long-range chromatin interaction, as shown by the purple arc. The numbers (0.12 and 0.57) above the purple arcs indicate ABC scores.

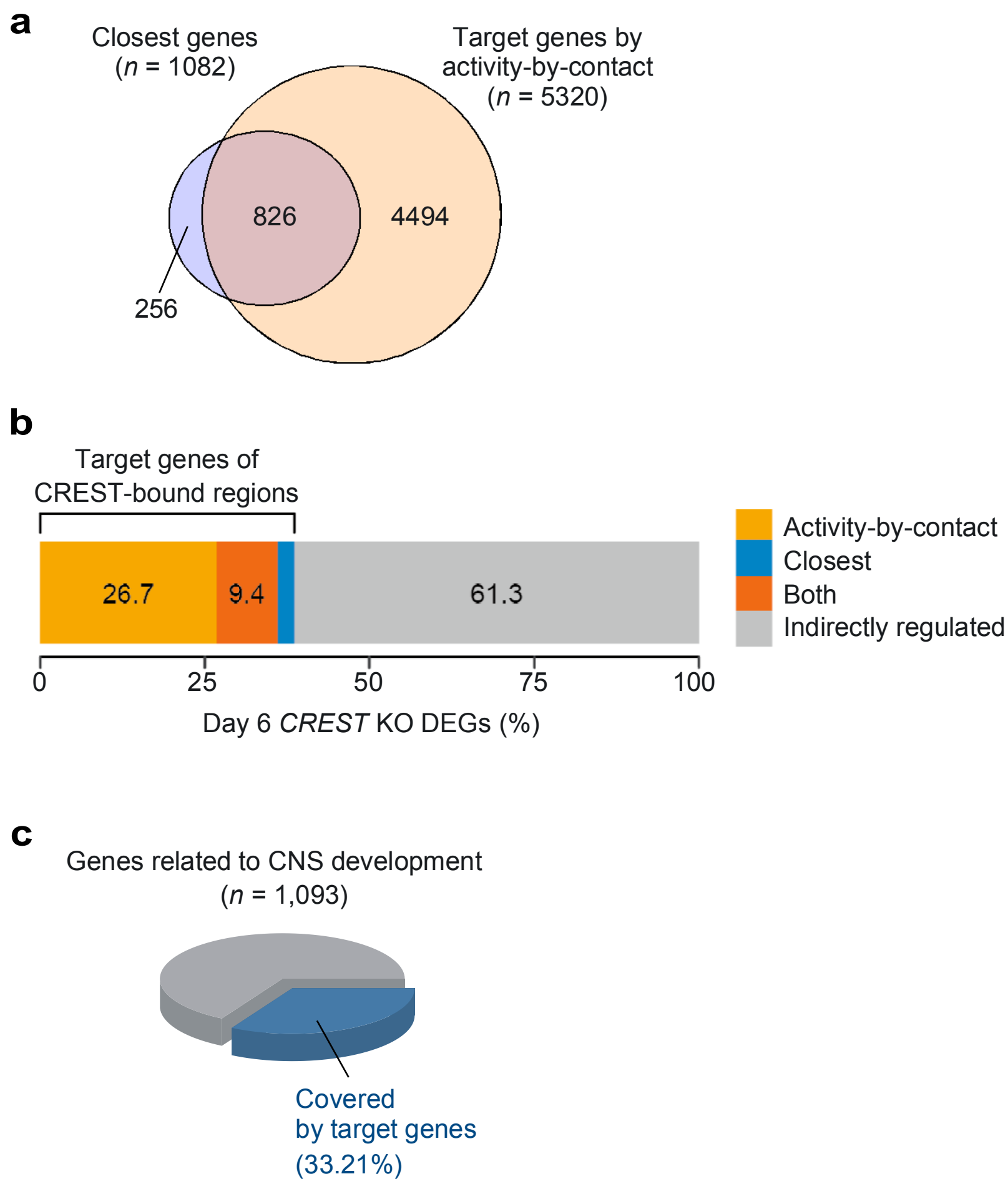

Supplementary Fig. 10 | Hi-C analysis demonstrates that CREST activates distal *cis*-regulatory regions of CNS developmental genes.

a, Venn diagram showing the overlap between the closest proximity genes, defined in **Fig. 3c** (*n* = 1082 genes; Supplementary Data 5), and the distal target genes (*n* = 5320 genes) of CREST-bound accessible regions, as identified by Hi-C Activity-by-Contact (ABC) analysis.

b, Stacked bar plot showing the proportion of day 6 *CREST* KO differentially expressed genes (DEGs) associated with CREST-bound accessible regions. These target genes are identified either by proximal localization (the closest genes) or Hi-C ABC analysis. The “Both” category (9.4%) indicates target genes identified by both methods.

c, Pie chart illustrating the proportion of central nervous system (CNS) development-related genes that overlap with the target genes of CREST-bound distal regions. Specifically, 363 out of the 1082 target genes overlap with the CNS-related gene set (*n* = 1093 genes), curated from the Mouse Genome Informatics (MGI) database.

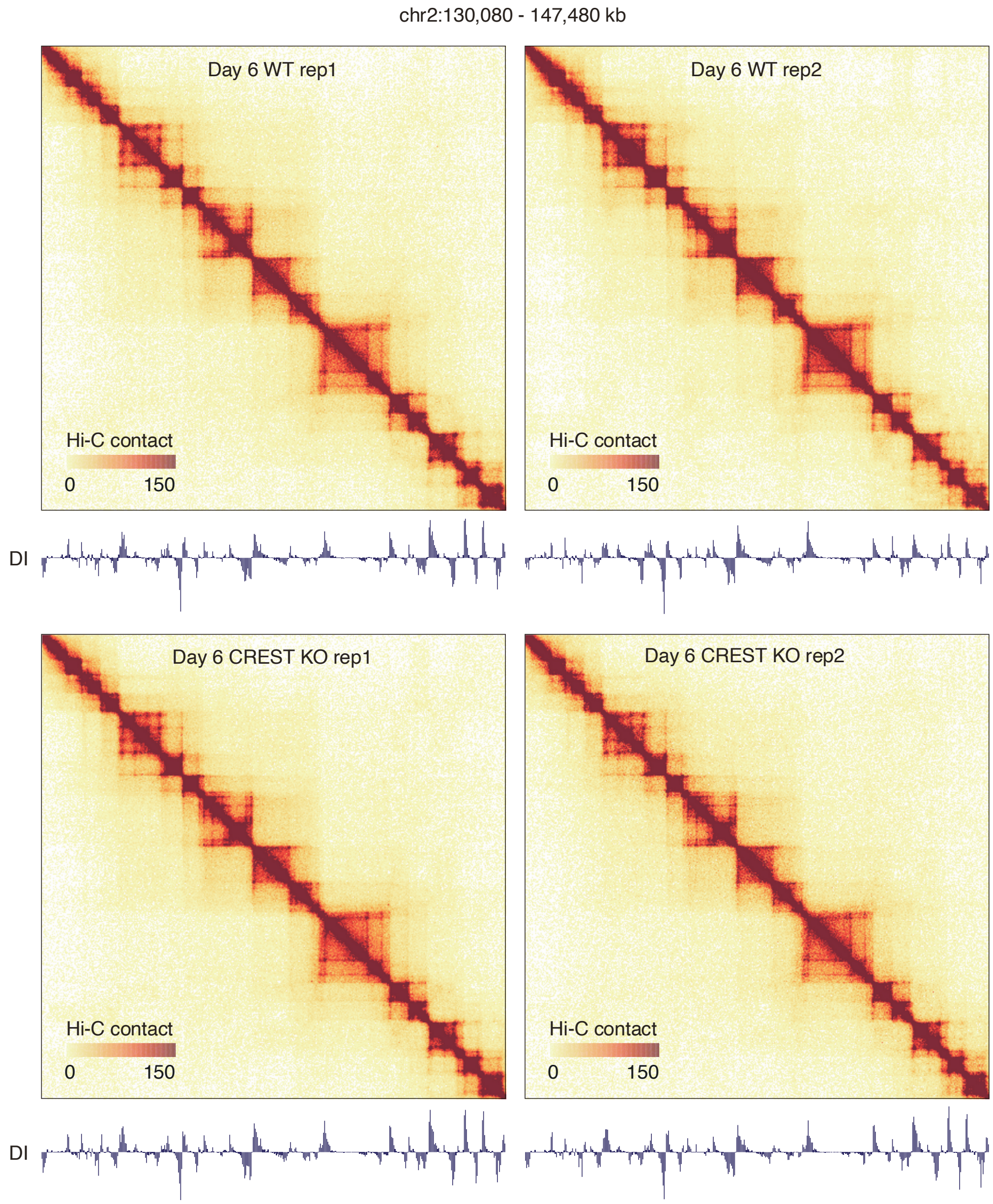

Supplementary Fig. 11 | **High-resolution 3D chromatin contact maps of day 6 WT and KO cells.**

Normalized three-dimensional (3D) Hi-C contact maps of day 6 WT motor neurons (top) and day 6 KO cells (bottom), displaying chromosome region chr2:130,080,000-147,480,000 from two biological replicates at 40 kb resolution, along with directionality index (DI) tracks. DI values in the bottom track indicate a preference for making chromatin contacts with upstream (negative DI) and downstream (positive DI) genomic regions.

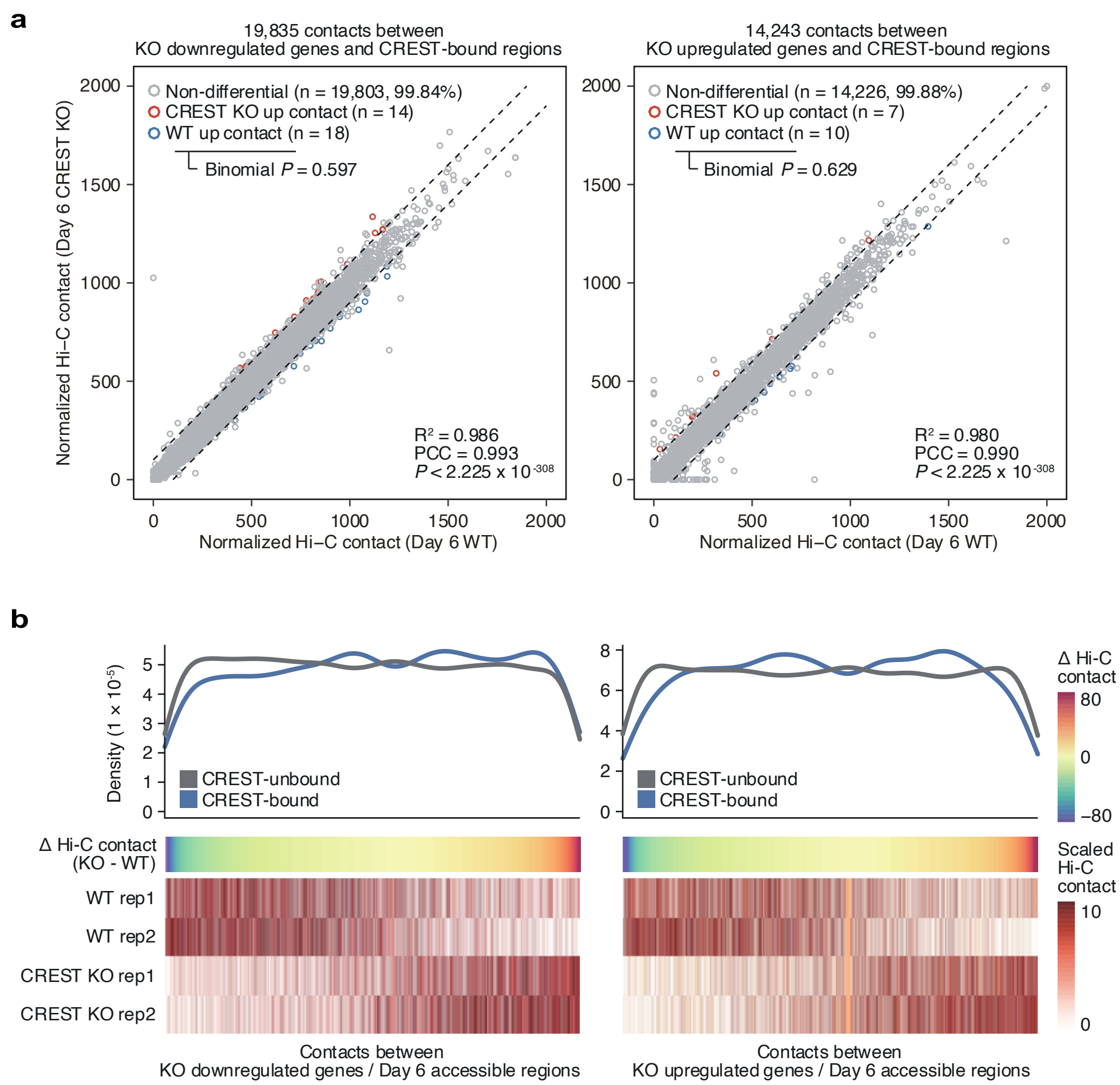

Supplementary Fig. 12 | **Genome-wide 3D chromatin contacts in CREST-bound distal regions remain unchanged in day 6 KO cells.**

a, Scatter plots of normalized Hi-C contacts between CREST-bound distal regions and differentially expressed genes in day 6 KO compared to day 6 WT motor neurons, including KO downregulated genes/CREST-bound regions (left) and *CREST* KO upregulated genes/CREST-bound regions (right). The contacts were considered differential if the difference in normalized Hi-C frequencies was > 100 (dashed lines) and $P < 0.01$, highlighted in red for day 6 *CREST* KO up contacts and blue for day 6 WT up contacts. The preferential inclusion of differential contacts was assessed using the two-sided exact binomial test. The significance *p*-values for preferential inclusion of differential contacts (binomial test) and correlation of normalized contacts between day 6 WT motor neurons and day 6 KO cells (Pearson correlation test) are indicated.

b, Density plots illustrating the distribution of chromatin contacts between differentially expressed genes and CREST-bound and -unbound distal regions with respect to differences in Hi-C contacts ranked in increasing order, showing *CREST* KO downregulated genes/day 6 accessible regions (left) and *CREST* KO upregulated genes/day 6 accessible regions (right).

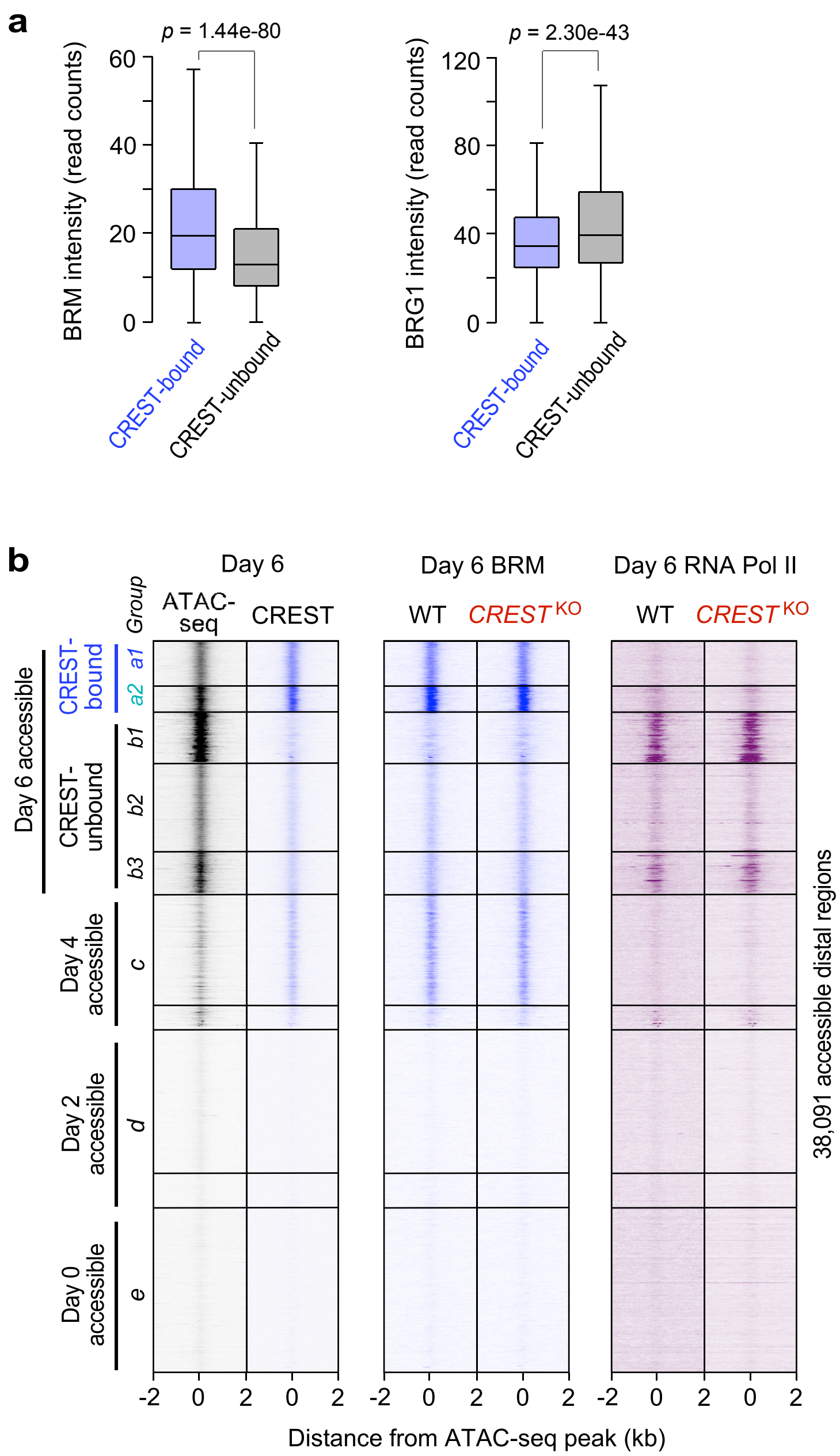

Supplementary Fig. 13 | **BRM ATPase, not BRG1, is involved in the accessibility of CREST-bound distal regions.**

a, Box plots of BRM and BRG1 ChIP-seq intensity (read counts) at CREST-bound (*Groups a1* and *a2*) and -unbound (*Groups b1-b3*) distal regions shown in **Fig. 5a**, **d**. The box plots display the median (line within boxes), first-to-third quartiles (bottom and top of boxes, respectively), and 1.5x the interquartile range (whiskers). *P*-values were calculated by a two-sided, two-sample Student's *t*-test.

b, Mapping of ATAC-seq and ChIP-seq CREST in day 6 WT motor neurons (*left*), and ChIP-seq BRM (*middle*) and RNA polymerase II (phosphorylated on the C-terminal domain serine 5; *right*) in day 6 WT motor neurons and day 6 KO cells. All data were plotted relative to 38,091 ATAC-seq peak midpoints in the same order as shown in **Fig. 5a**.

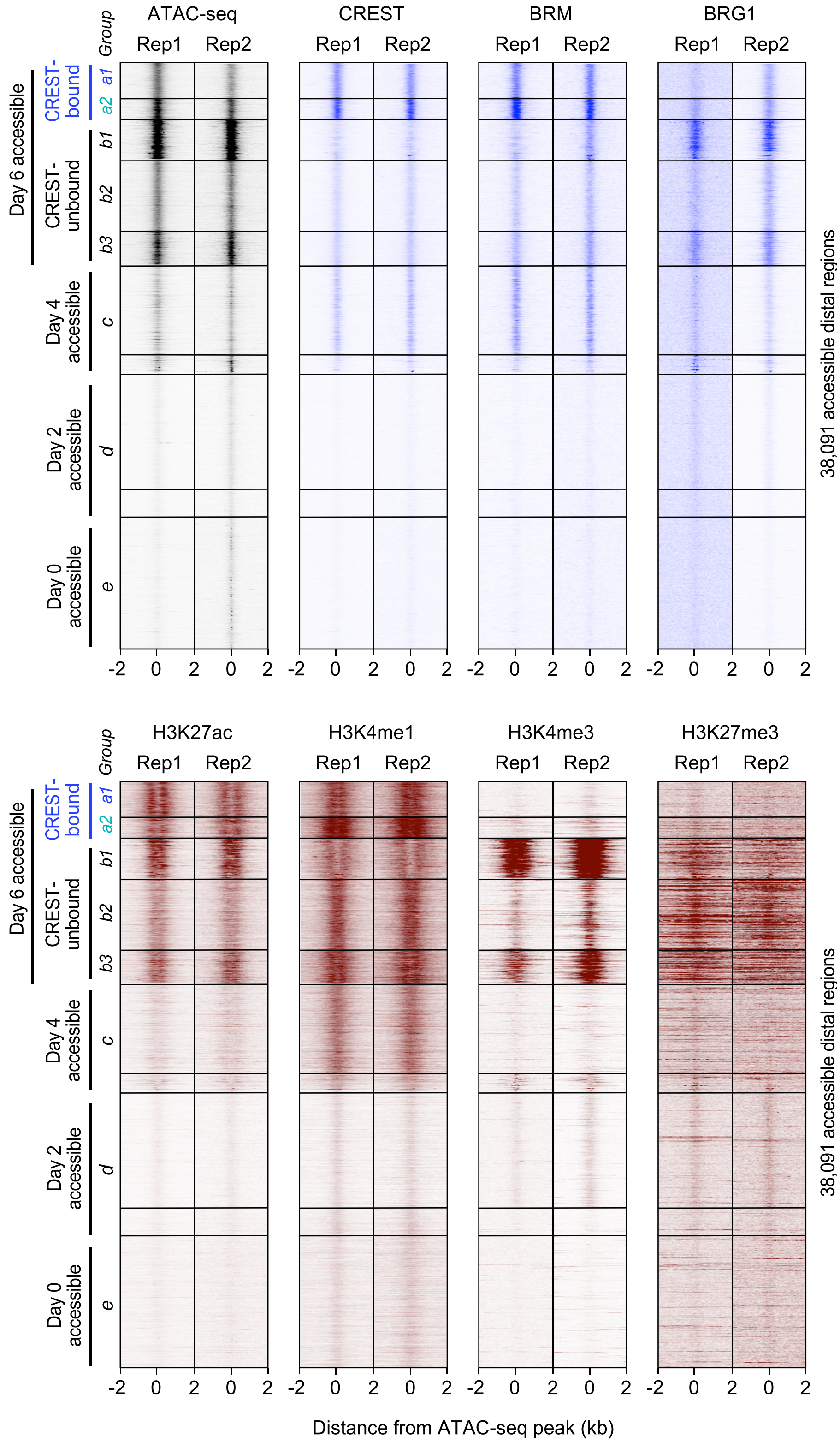

Supplementary Fig. 14 | ChIP-seq results in Fig. 5 are reproducible with independent motor neuron differentiations. ChIP-seq mapping of CREST, BRM, BRG1, H3K27ac, H3K4me1, H3K4me3, and H3K27me3, as well as ATAC-seq mapping at 38,091 accessible distal *cis*-regulatory regions in day 6 WT motor neurons, shown in **Fig. 5**, with two replicates of independent differentiations. All data were plotted relative to the ATAC-seq peak midpoints in the same order as shown in **Fig. 5a**.

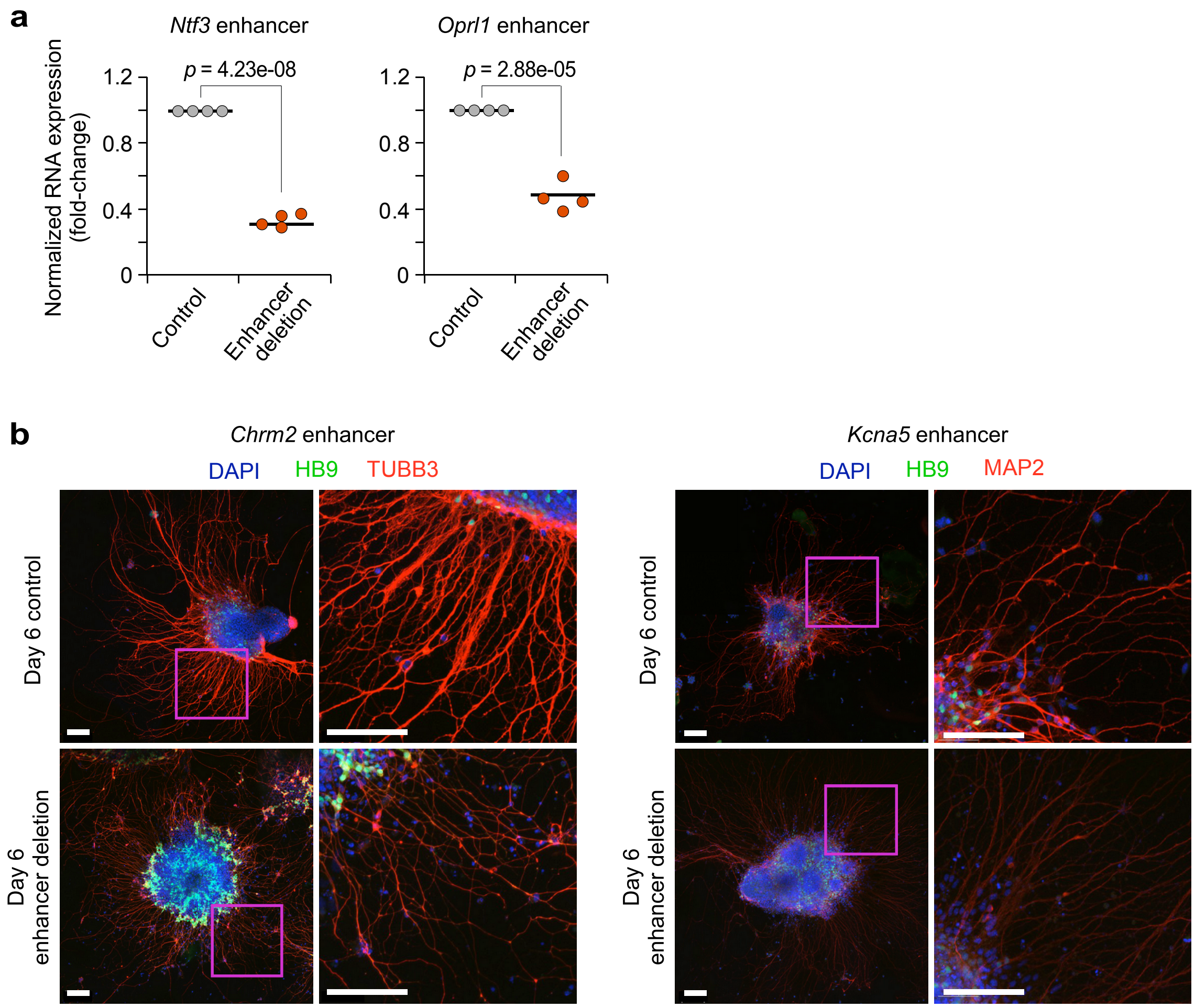

Supplementary Fig. 15 | **CREST-bound enhancers regulate the transcription of neural genes and promote neurite outgrowth.**

a, Fold-change in the expression of neuronal genes, *Ntf3* and *Oprl1,* measured by RT-qPCR in day 6 mutant cells harboring deletions of their respective CREST-bound enhancers (indicated by arrows in Supplementary Fig. 9b; 1.7 kb for *Ntf3* and 1.1 kb for *Oprl1*). Expression levels are normalized to day 6 WT motor neurons. Enhancer deletions were generated in ES cells using CRISPR/Cas9 genome editing before a six-day motor neuron differentiation. *P*-values were calculated by a two-sided, two-sample Student's *t*-test (two cell differentiations with two replicates, $n = 4$ independent experiments). Lines in the dot plots show the average.

b, Immunostaining of differentiated embryoid bodies on laminin-coated surfaces showing the nuclear-specific dye DAPI, the motor neuron-specific marker HB9, the neuronal marker TUBB3 (class III β-tubulin), and the mature neuronal marker MAP2 (microtubule-associated protein 2) in day 6 WT control and the two day 6 enhancer deletion cell lines (*Chrm2* and *Kcna5* enhancer deletions), as shown in **Fig. 6a**. Magenta boxes in the left panels indicate the regions at higher magnification on the right. Scale bars represent 100 µm.

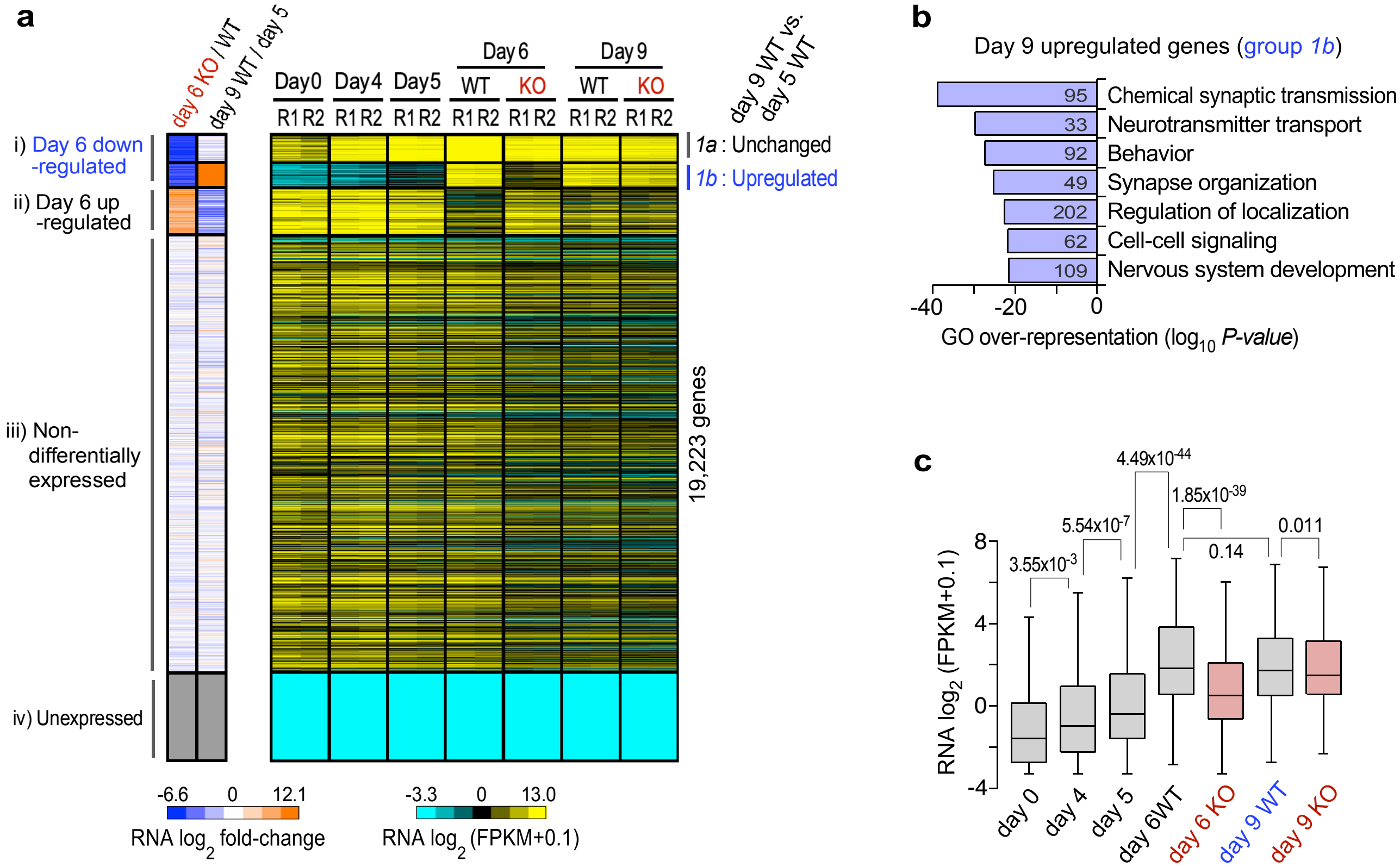

Supplementary Fig. 16 | ***CREST* knockout delays neuronal maturation, extending the differentiation process from 6 days to 9 days.**

a, *Left*: Heatmap of RNA expression (log_2 fold-changes) of all 19,223 mRNA genes in day 6 KO cells compared with day 6 WT cells. Genes are ordered as displayed in **Fig. 2a**, except for the Day 6 downregulated genes (Group *i*), which were further subdivided based on RNA expression changes in day 9 WT cells compared with day 5 WT cells (Group *1a*: 879 unchanged or downregulated genes; Group *1b*: 735 upregulated genes). *Right*: Heatmap of RNA expression (log_2 FPKM+0.1) of all mRNA genes in days 0, 4, 5, and 6 WT and day 6 KO cells, as well as day 9 WT and day 9 KO cells. FPKM values were adjusted by adding 0.1, median-normalized, log_2-transformed, and ordered as displayed on the *left*. The heatmap includes data from two biological replicates (R1 and R2) derived from independent cell differentiations.

b, GO analysis of 735 upregulated genes (Group *1b*) identified in panel (**a**). The seven most over-represented and non-redundant GO terms are displayed. Numbers within bars represent the count of genes associated with each GO term.

c, Box plots showing RNA expression (log_2 FPKM+0.1) for 95 upregulated chemical synaptic transmission genes (Group *1b*, the top GO term genes), identified in panel (**b**). FPKM values represent averages from two biological replicates, shown in panel (**a**). The plots display the median (line within boxes), first-to-third quartiles (bottom and top of boxes, respectively), and 1.5x the interquartile range (whiskers). A two-sided paired *t*-test.

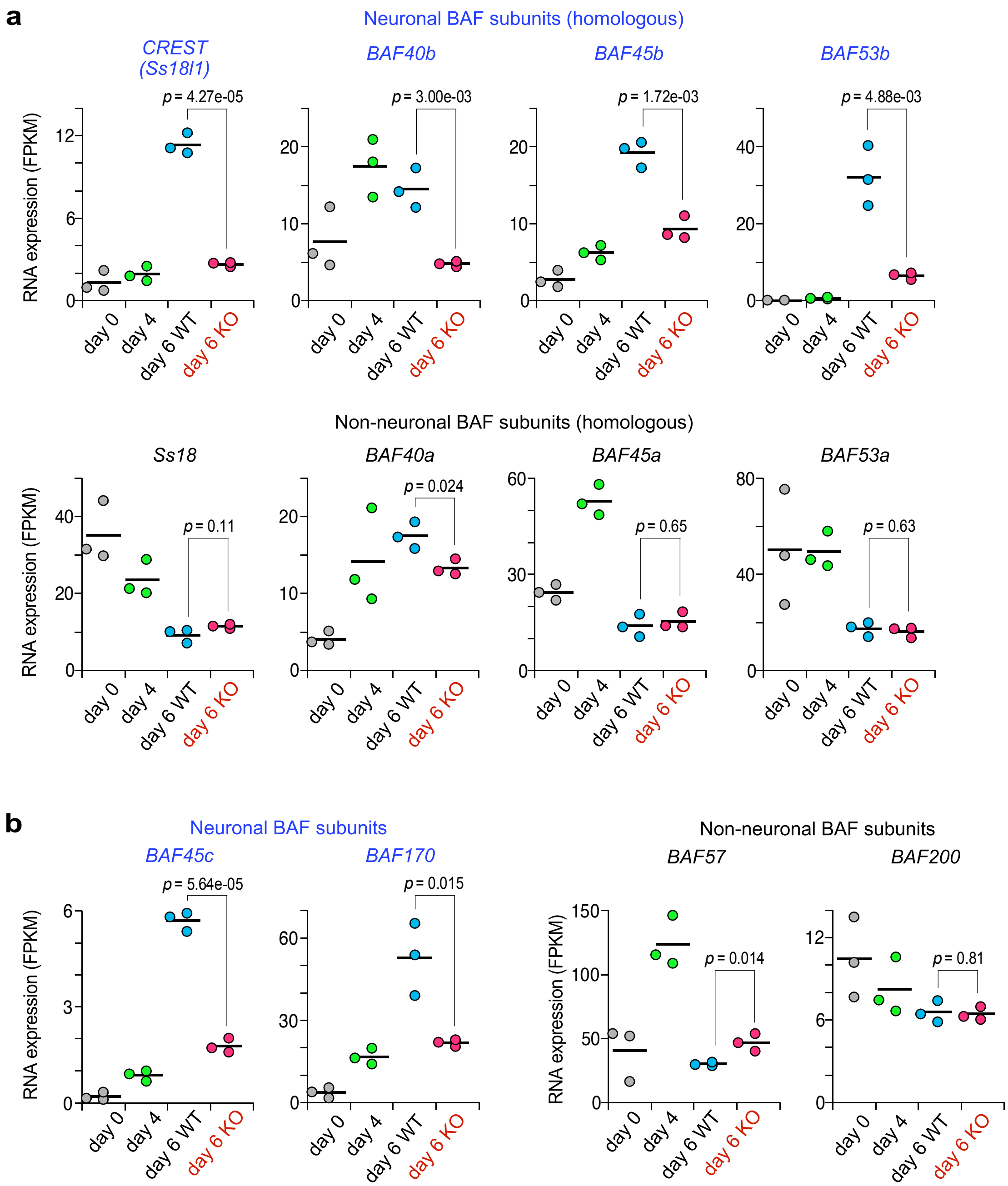

Supplementary Fig. 17 | **CREST knockout downregulates the mRNA expression of specific neuronal BAF subunits during motor neuron differentiation.**

a, RNA expression (FPKM) of four genes encoding neuronal BAF subunits and four genes encoding their homologous non-neuronal BAF subunits, measured by RNA-seq during WT motor neuron differentiation (day 0 ES cells, day 4 neural progenitors, day 6 WT motor neurons) and in day 6 KO cells from three biological replicates (n = 3 experiments) of independent cell differentiations (Supplementary Data 4). Horizontal lines in the dot plots show the average. P-values were calculated by a two-sided, two-sample Student's t-test.

b, RNA expression (FPKM) of two genes encoding neuronal BAF subunits and two genes encoding non-neuronal BAF subunits, measured by RNA-seq during WT motor neuron differentiation and in day 6 KO cells from three biological replicates (n = 3 experiments) of independent cell differentiations (Supplementary Data 4). Horizontal lines in the dot plots show the average. P-values were calculated by a two-sided, two-sample Student's t-test.

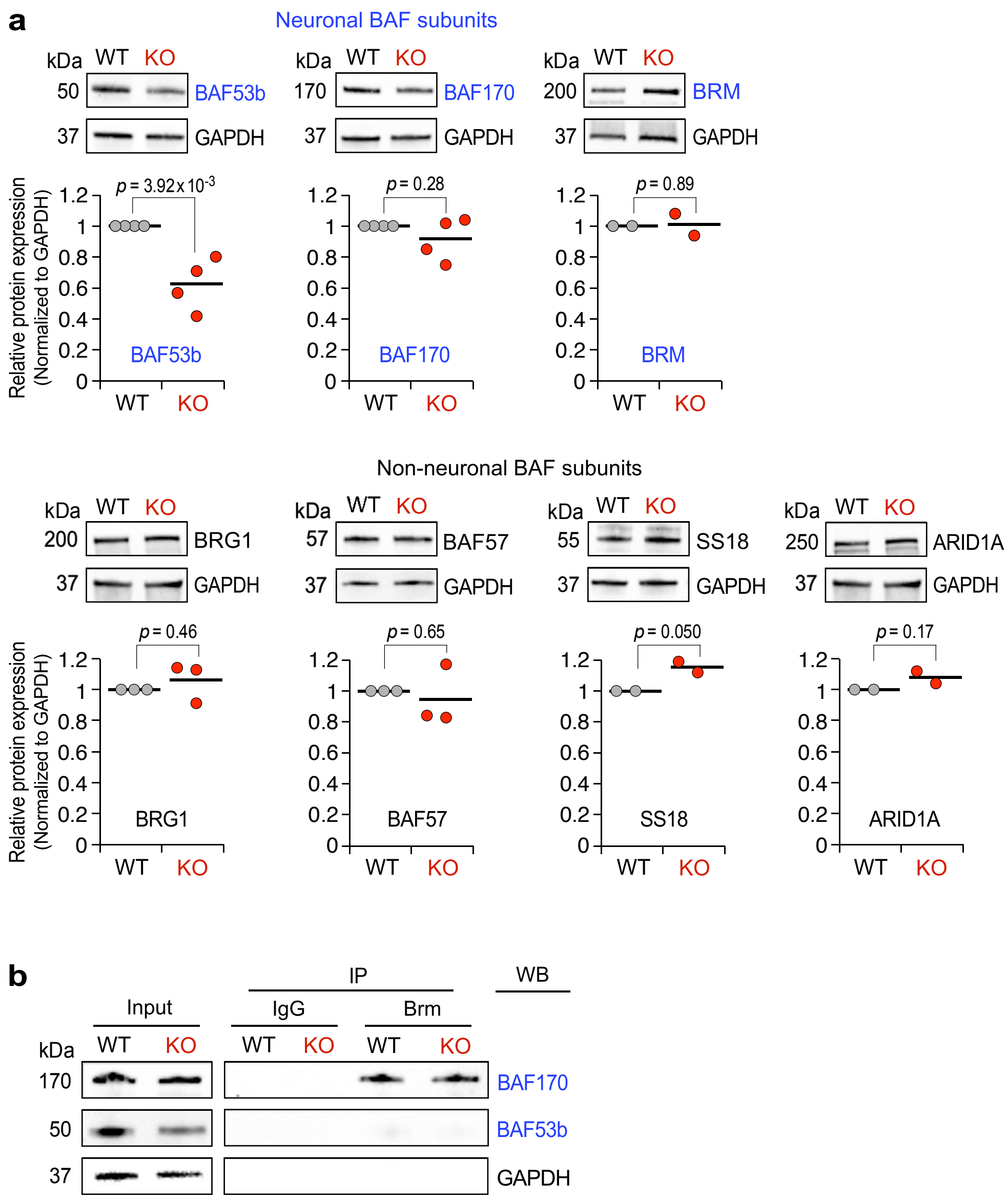

Supplementary Fig. 18 | ***CREST* knockout downregulates the protein levels of specific neuronal BAF subunits in day 6 differentiated cells.**

a, Western blot analysis of three neuronal BAF subunits (BAF53b, BAF170, and BRM) and four non-neuronal BAF subunits (BRG1, BAF57, SS18, and ARID1A) in day 6 WT and *CREST* KO cells. Detected bands correspond to ~50 kDa (BAF53b), ~170 kDa (BAF170), ~200 kDa (BRM and BRG1), ~57 kDa (BAF57), ~55 kDa (SS18), ~250 kDa (ARID1A), and ~37 kDa (GAPDH, loading control). ARID1A and BRG1 were resolved on the same gel, sharing the same GAPDH loading control. Horizontal lines in the dot plots represent the average. *P*-values were calculated using a two-sided, two-sample Student's *t*-test. A representative immunoblot from biological replicate 1 is shown here. All raw densitometric intensity values and immunoblot images for additional biological replicates of BAF53b and BAF170 are available in Supplementary Data 3 and 11.

b, Biochemical demonstration of the interactions of BRM with BAF170 and BAF53b in day 6 WT and day 6 KO cells. Immunoprecipitation (IP) of day 6 cell lysates was performed using an anti-BRM antibody, followed by Western blot (WB) analysis for BAF170, BAF53b, and GAPDH (used as a negative control). The detected bands correspond to ~170 kDa (BAF170), ~50 kDa (BAF53b), and ~37 kDa (GAPDH). The input consists of 25 µg of nuclear extract; normal IgG served as a negative control for the IP (Supplementary Data 3).

SUPPLEMENTARY METHODS

Experimental Methods

Cell culture and differentiation. The embryonic stem (ES) cell line originated from the male embryo of the inbred mouse strain 129/Ola ES-E14. ES cells were cultured and differentiated into motor neurons as described.[1] Briefly, ES cells were cultured for two days on 0.1% gelatin-coated plates in ES growth medium (EmbryoMax DMEM [Millipore, SLM220] supplemented with 15% ES cell-grade fetal bovine serum [Wisent, 920-040], 2 mM L-glutamine [Gibco, 25030-081], 1x EmbryoMax non-essential amino acid [Millipore, TMS-001-C], 1x Nucleosides [Millipore, ES-008-D], 0.36 μM beta-mercaptoethanol [Sigma Aldrich, M6250], and 1000 U/mL ESGRO leukemia inhibitory factor [Millipore, ESG1107]) at 37°C and 5% CO_2. To begin the formation of embryoid bodies, clusters of differentiated cells. ES cells were trypsinized (Gibco, 25300-054) on day 0 and seeded at 8 x 10^5 cells per mL in ADFNK medium (Advanced DMEM/F12 (Gibco, 12634-010) and Neurobasal Medium (Gibco, 21103-049) at a 1:1 ratio, 10% Knockout Serum Replacement (Gibco, 10828-028), 2 mM L-glutamine, and 0.4 uM beta-mercaptoethanol). ADFNK medium was changed on days 2 and 5 of the differentiation. On day 2, ADFNK medium supplemented with 1 µM all-trans retinoic acid (Sigma Aldrich, R2625) and 0.25 µM Smoothened agonist of hedgehog signaling (Selleckchem, S7779) was added to the embryoid bodies. On days 4 and 5, ADFNK medium supplemented with 5 μM gamma-secretase inhibitor (Selleckchem, S2215) was added to the differentiating cells. Differentiated cells were collected on day 6 and used for downstream experiments such as western blotting, immunostaining, RNA-seq, ChIP-seq, ATAC-seq, and Hi-C.

For day 9 neuronal culture, embryoid bodies were collected on day 4 and plated onto coverslips coated with 0.001% poly-L-ornithine (Sigma, A-004-M) and 1% Matrigel (VWR, 356234). Cells were cultured in a 1:1 mixture of Advanced DMEM/F12 (Thermo, 12634010) and Neurobasal Medium (Gibco, 21103049) supplemented with 2% B-27 (Gibco, A3582801), horse serum (Gibco, 16050130), 2 mM L-glutamine (Gibco, 25030081), 0.1 mM β-mercaptoethanol, and penicillin-streptomycin (Thermo, 15140122). The culture medium was replaced on day 6 and day 8. On day 9, the attached cells were harvested for RNA-seq. For day 6 neuronal culture, procedures were identical to those for day 9 neuronal culture, except that embryoid bodies were collected on day 4 and plated onto coverslips coated with 0.001% poly-L-ornithine (Sigma, A-004-M) and 4 µg/mL Matrigel (Corning, 356231).

Generation of the CREST mutant cell line. The ES cell line, derived from the inbred mouse strain 129/Ola ES-E14 male embryo, was used for homozygous *CREST* knockout. CRISPR/Cas9 genome editing was performed by following Ran et al.[2] and Jaura et al.[1] with some alterations. Briefly, using software from CRISPOR (http://crispor.tefor.net/crispor.py) and CHOPCHOP (https://chopchop.cbu.uib.no/), guide RNAs (gRNAs) were designed to target the left and right sides of the *CREST* gene and cause a frameshift deletion. The pair of oligonucleotide guide sequences were 23 bp and of the form 5'-$(N)^{20}$-NGG-3' (N = A, T, G, or C; PAM = protospacer adjacent motif is underlined). The gRNA sequences were designed to target a 710 bp region of CREST intron-exon 2 (chromosome 2):
Forward (left gRNA) 5' GTACACGTGGTAGCGCTGGGAGG 3' (reverse/negative strand)
Reverse (right gRNA) 5' TACTCACTGCGTGCACTCGGCGG 3' (reverse/negative strand)
The 23 bp guide sequences were synthesized from Integrated DNA Technologies, Inc. (Coralville, IA, USA), and then cloned into a U6 target gRNA expression plasmid (Addgene, Plasmid #41824). Next, DNA cloning was performed as described[3] with a few modifications. ES cells were seeded on mouse embryonic fibroblast cells and incubated for 48 hours. ES cells were then transiently nucleofected with the pair of gRNA expression

vectors (left and right gRNAs), and the pCas9-mCherry plasmid (a gift from H. Wichterle), which contained the Cas9 DNA endonuclease and the reporter gene mCherry. To perform the nucleofection, P3 Primary Cell 4D-Nucleofector X Kit (Lonza, V4XP-3024) and Amaxa 4D-Nucleofector (Lonza, AAF-1003B) were used, according to the manufacturer's instructions. 40-48 hours after nucleofection, the ES cells were dissociated and selected by fluorescence-activated cell sorting (FACS) based on mCherry reporter expression. Approximately 2400 single ES cells were plated on mouse embryonic fibroblast cells and grown for 4-6 days to form clones. Individual colonies were picked and genotyped using PCR primers. Genomic DNA from the ES cell lines containing the CREST knockout was Sanger sequenced to confirm the deletion. Finally, the ES cell clones containing a homozygous frameshift deletion were sub-cultured onto mouse embryonic fibroblast cells to be frozen for later use in the motor neuron differentiation protocol as described above. Procedures for generating heterozygous CREST KO mutations were identical to those for homozygous mutations, except that ES cell clones containing a heterozygous deletion were selected following genotyping.

Western blotting. Protein extracts were prepared by resuspending cell pellets in Western Lysis buffer (150 mM NaCl, 1% Triton X-100, 0.5% sodium deoxycholate, 0.1 sodium dodecyl sulfate, 50 mM Tris-HCl, pH 8.0) supplemented with complete protease inhibitor (Roche, 04693159001) using 25-gauge syringe needles. Resuspended cells were incubated on ice for 20 minutes and then centrifuged at 15,870 x g for 15 minutes at 4°C to pellet the debris. 4x Laemmli sample buffer (BioRad, 1610747) and 40x β-mercaptoethanol were added to the protein samples and the mixture was heated at 95°C for 5 minutes as described.[4] Equivalent concentrations of samples were loaded in 4-15% Mini-PROTEAN TGX Precast Gels (BioRad, 4561085) and run using Novex Bolt Mops sodium dodecyl sulfate (SDS) Running Buffer (Life Technologies, B0001) to separate proteins. After gel electrophoresis, the proteins were dry transferred to a 0.2 μm nitrocellulose membrane using iBlot 2 nitrocellulose Regular Stacks (Invitrogen, IB23001) with the iBlot 2 Dry Blotting System (Invitrogen, IB21001). The membrane was blocked in phosphate-buffered saline (PBS)-T (0.1% Tween-20) containing 6% bovine serum albumin (BSA) at 25°C for 2 hours. Afterward, the following two primary antibodies were diluted in 6% BSA/PBS-T and incubated with the nitrocellulose membrane at 4°C overnight: anti-CREST (Proteintech, 12439-1-AP; lot#, 00042079), anti-GAPDH (Cell Signaling, 2118; lot# 082020-14), anti-MAP2 (Cell Signaling, 4542S; lot# 032019-4), anti-SS18 (Cell Signaling, 21792; lot# 092029-1), anti-ChAT (Millipore Sigma, AB144P; lot# 4254299), anti-BAF170 (Cell Signaling, 12760; lot# 052030-2), anti-BAF53b (Antibodies Incor., 75-311; lot# 449-4AK-23), anti-BRM (Bethyl Lab, A301-015A; lot# 2), anti-ARID1A (Cell Signaling, 12354S; lot# 022025-4), anti-BRG1 (Abcam, ab110641; lot# 1141327-3), and anti-BAF57 (Bethyl Lab, A300810AT; lot#, 2). The membranes were washed five times using PBS-T to remove residual primary antibodies. The following secondary antibody conjugated to horseradish peroxidase (HRP) was diluted in 6% BSA/PBS-T and incubated with the nitrocellulose membranes at 25°C for 1 hour: anti-rabbit IgG HRP (Santa Cruz, sc2357; lot# E0819). Five more washing steps with PBS-T were followed to remove residual secondary antibody. Finally, membranes were developed using a SuperSignal West Pico PLUS Chemiluminescent Substrate (Fisher Scientific, 34577) and imaged using the iBright 1500 Imager (Invitrogen). Densitometric intensity values for Supplementary Fig. 18a were calculated using ImageJ (https://imagej.net/ij/) and are reported in Supplementary Data 11. For Western blot quantification, target protein band intensities were normalized to their corresponding GAPDH loading control intensities within each biological replicate. Immunoblot images (unprocessed scans) of Western blotting and co-IP experiments are shown in Supplementary Data 3.

Immunofluorescence staining. Day 6 embryoid bodies, which are clusters of differentiated motor neurons, were fixed in 4% paraformaldehyde in PBS at 25°C for 5 minutes, then embedded in Tissue-Plus O.C.T

Compound (Fisher Scientific, 4585) and frozen. The frozen embryoid bodies were sectioned on a cryostat, placed on microscope glass slides, washed twice with PBS, then blocked with Staining Blocking Solution (10% fetal bovine serum or donkey serum, 0.3% Triton X-100 (BioShop Inc., TRX506.500), and 0.05% sodium azide (Sigma-Aldrich, S2002) in PBS) at 25°C for 30 minutes. Next, the sectioned embryoid bodies were washed three more times with PBS, once with Staining Dilution Buffer (2% fetal bovine serum or donkey serum, 0.1% Triton X-100, and 0.05% sodium azide in PBS), then stained overnight at 4°C with the following primary antibodies in Staining Dilution Buffer: anti-CREST (Proteintech, 12439-1-AP; lot#, 00042079), anti-HB9 (DSHB, 81.5C10), anti-ISL1 (Neuromics, GT15051), and anti-H3K27ac (Abcam, ab4729; lot# 1059037-6). The sectioned embryoid bodies were washed three more times in PBS then incubated at 25°C for 1 hour with the following secondary antibodies in Staining Dilution Buffer: anti-goat IgG Alexa Fluor 488 (Thermo, A11055; lot# 1942238), anti-mouse IgG Alexa Fluor 555 (Thermo, A31570; lot# 1905844), and anti-rabbit IgG Alexa Fluor 647 (Thermo, A31573; lot# 1964354). The stained neurons on microscope glass slides were treated with DAPI at 25°C for 20 minutes, then mounted with Mounting Medium containing DAPI (Abcam, ab104139, lot# GR3274489-1) and stored at 4°C. For cell quantification, at least five embryoid bodies per time point or condition (averaging 393 H3K27ac-positive cells per embryoid body) were randomly selected. Cells were scored for the expression of the motor neuron markers HB9 and ISL1 using CellProfiler (v4.2.8).

For day 6 neuronal culture, embryoid bodies containing differentiated day 6 motor neurons attached to coverslips were washed once with PBS and fixed with 4% paraformaldehyde in PBS for 20 minutes at room temperature. Following fixation, the cells were washed once with 0.1% BSA in ice-cold PBS, permeabilized with 0.035% Triton X-100 for 15 minutes, and blocked for 30 minutes at room temperature. The cells were then incubated overnight at 4°C with the following primary antibodies: anti-MAP2 (Cell Signaling, 4542S; lot# 032019-4), anti-HB9 (DSHB, 81.5C10), and anti-TUBB3 (Sigma, T2200-200ul). The following day, cells were washed three times with 0.1% BSA in ice-cold PBS and incubated with an anti-rabbit secondary antibody (Thermo, A31573) for 1 hour at room temperature. After a final wash with 0.1% BSA in ice-cold PBS, coverslips were then mounted onto slides using a mounting medium containing DAPI (Abcam, ab104139) and allowed to dry overnight at 4°C before imaging.

Alkaline phosphatase assay. Wild-type and CREST KO ES cells were plated on 0.1% gelatin-coated plates in EmbryoMax DMEM (Millipore, SLM220) supplemented with 10% ES cell-grade fetal bovine serum (Invitrogen, 12483020), 2 mM L-glutamine (Gibco, 25030081), 0.1 mM β-mercaptoethanol, and leukemia inhibitory factor (Millipore, ESG1107). After two days of culture, staining was performed using an Alkaline Phosphatase Staining Kit (Abcam, ab242287). The ES cell colonies were imaged at 10X magnification under a light microscope.

RNA-seq. RNA-seq libraries were prepared as described with minimal modifications.[1] Briefly, total RNA was extracted from differentiated cells using TRIzol™ reagent (Invitrogen, 15596018). Total RNA was treated with DNase (Invitrogen, AM1906) to remove DNA contamination. NEBNext Poly(A) mRNA Magnetic Isolation Kit (New England Biolabs, E7490) was used on the DNase-treated RNA samples to enrich mRNA. Complementary DNA was synthesized and RNA-seq libraries were prepared using NEBNext Ultra II Directional RNA Library Prep Kit (New England Biolabs, E7760). Finally, RNA-seq libraries were sequenced on Illumina Sequencing Platforms.

ChIP-seq. Approximately 30 million cells were collected for ChIP-seq. Cells for ChIP-seq CREST and CBP were double-crosslinked with disuccinimidyl glutarate (Thermo Scientific, AAH58208MC) for 30 minutes, followed by 1% formaldehyde for 15 minutes, and lysed. Cells for all other ChIP-seq samples were crosslinked using 1% formaldehyde only and then lysed. Chromatin pellets were isolated, solubilized, and fragmented by sonication.

Fragmented chromatin was immunoprecipitated as described with minimal modifications.[1] Briefly, Protein G- or Protein A-coupled magnetic beads (Invitrogen, 10001D or 10004D) were used to immunoprecipitate chromatin against target antibodies: anti-CREST (ProteinTech, 12439-1-AP; lot#, 00042079), anti-CBP (Cell Signaling, 7389S; lot# NA), anti-p300 (Abcam, ab275378; lot# 1074860-31), anti-p300 (Sigma, 05-257), anti-TIP60 (ProteinTech, 10827-1-AP; lot # 00138365), anti-IgG (Cell Signaling, 2729; lot# 11), anti-BRM (Bethyl, A301-015A; lot# 2), anti-BRG1 (Abcam, ab110641; lot# 1000647-1), anti-H3K27ac (Abcam, ab4729; lot# GR3251519-2), anti-H3K4me1 (Abcam, ab8895; lot# GR3442591-2), anti-H3K4me3 (Abcam, ab8580; lot# GR3362386-3), anti-H3K27me3 (Abcam, ab6002), anti-CTCF (Millipore, 07-729; lot# 3515588), and anti-RPB1 (Cell Signaling, 2629; lot# 062025-4). Fragmented chromatin was added to the protein-coated beads and rocked overnight at 4°C. The immunoprecipitated chromatin was washed, eluted, and reverse crosslinked. Proteins and RNA were degraded, and then the DNA was purified using NucleoMag NGS Clean-up and Size Selection Beads (TaKaRa, 744970). ChIP-seq libraries were prepared for sequencing using the NEBNext Ultra II DNA Library Prep Kit (New England Biolabs, E7645). Briefly, ChIP DNA was end-prepped, adaptors were ligated, DNA was purified and size-selected again, then amplified using ligation-mediated PCR. Finally, ChIP-seq libraries were gel-purified and sequenced by Illumina Sequencing Platforms.

ATAC-seq. ATAC-seq samples were prepared for sequencing using the ATAC-seq Kit (Active Motif, 53150) with minimal modifications. Briefly, approximately 50,000 cells were collected for ATAC-seq. Cell pellets were isolated and resuspended in ATAC-seq Lysis Buffer (10 mM NaCl, 3 mM MgCl2, 0.1% octylphenoxypolyethoxyethanol [IGEPAL CA-630], 10 mM Tris-HCl, pH 7.4). Lyzed cell pellets were isolated, resuspended in Tagmentation Master Mix (Tn5 Transposase, 0.5% Digitonin, 10% Tween 20, 1x PBS), and incubated at 37°C for 30 minutes. Tagmented DNA was then purified, size-selected, and amplified using ligation-mediated PCR. Finally, the amplified ATAC-seq libraries were purified using NucleoMag NGS Clean-up and Size Selection Beads (TaKaRa, 744970) and sequenced on Illumina Sequencing Platforms.

Hi-C. In situ, Hi-C libraries were generated as described with minimal modifications.[5] Two biological replicates of day 6 CREST knockout cells and day 6 WT motor neurons (1×10^6 cells) were harvested and crosslinked in 1% formaldehyde for 10 min at 25°C. Crosslinked cells were lysed with 10nM Tris-HCl (pH 8.0), 10mM NaCl, and 0.2% IGEPAL CA630 (Sigma-Aldrich, 18896), and digested with 100U MboI (NEB, R0147). Digested fragments were labelled with biotin-14-dCTP (Invitrogen, 19518018) and proximally ligated with T4 DNA Ligase (NEB, M0202), followed by reverse-crosslinking with 2 μg/uL proteinase K (NEB, P8102), 1% SDS, and 500mM NaCl overnight at 68°C. The DNA fragments were purified with AMPure XP beads (Beckman Coulter, A63880), and subjected to sonication (Covaris, S220). The ligated DNA fragments were pulled down with Dynabeads MyOne streptavidin T1 beads (Invitrogen, 65602) and thoroughly washed. Hi-C libraries were prepared manually by performing DNA end repair, removal of unligated ends, adenosine addition at the 3' end (NEB, M0212), ligation of Illumina-indexed adapters (NEB, M2200), and PCR amplification (Thermo Fisher Scientific, F549). The number of cycles for PCR amplification was determined based on the KAPA library quantification kit (KAPA, KK4854). Finally, the DNA libraries were then subjected to deep sequencing in paired-end mode using the MGI DNBSEQ-G400 platform.

Co-immunoprecipitation. For co-immunoprecipitation (Co-IP) experiments, cells were at D6, then subjected to nuclear extraction using the Active Motif Nuclear Complex Co-IP kit (Active Motif 54001). Nuclear extracts were quantified using the Pierce BCA Protein Assay Kits (Thermo Fisher A65453) and either used immediately or stored at -80°C. Co-IP was performed following the Co-IP kit manufacturer's protocol. Briefly, 150 μg of nuclear

extract and 5 μg of anti-CREST antibody (Proteintech, 12439-1-AP; lot# 00042079) or anti-BRM antibody (Bethyl Lab, A301-015A; lot# 2) were incubated overnight at 4°C. A no-antibody negative control was also prepared using 100 μg of nuclear extract. Magnetic beads coupled with Protein A (Invitrogen 10001D) were added to each sample and rocked for 1 hour at 4°C. Beads were washed to remove non-specific proteins and DNA, and proteins were eluted by incubating in 4X Laemmli Sample Buffer (Bio-Rad 1610747) for 10 minutes at 95°C. 75% of each Co-IP sample was loaded onto Mini-Protean TGX Precast Gels (Bio-Rad 4561085), alongside 25 μg of nuclear extract as a staining control. Electrophoresis was performed at 100 volts for 1.5 hours in an SDS-PAGE running buffer, followed by protein transfer to 0.45 mm PVDF membranes (Sigma IPVH07850) using wet transfer at 100 volts for 2 hours. Gel electrophoresis and transfer were performed using the Mini-PROTEAN Tetra Cell system (Bio-Rad 1658033). Membranes were blocked in PBS-T (0.1% Tween-20) containing 3% bovine serum albumin (BSA) for 1 hour at room temperature and incubated overnight at 4°C with primary antibodies: anti-BRM (Bethyl Lab, A301-015A; lot# 2), anti-ONECUT1 (Santa Cruz, sc-376167; lot# J2417), anti-NGN2 (Cell Signaling, 13144; lot# 052030-3), anti-β-Actin (Santa Cruz, sc-47778; lot# H1420), anti-BAF170 (Cell Signaling, 12760; lot# 052030-2), anti-BAF53b (Antibodies Incor., 75-311; lot# 449-4AK-23), and anti-GAPDH (Cell Signaling, 2118; lot# 082020-14). For chemiluminescence detection, membranes were incubated for 1 hour with HRP-conjugated goat anti-mouse secondary antibody (Proteintech, SA00001-1, lot# NA) or HRP-conjugated mouse anti-rabbit secondary antibody (Santa Cruz, sc-2357; lot# E0819), developed with SuperSignal West Pico PLUS Chemiluminescent Substrate (Thermo Fisher 34580), and imaged with the iBright FL1500 Imaging System.

Data Analyses

RNA-seq analysis. RNA-seq raw data (FASTQ) files were aligned to the mouse genome (Mus musculus, mm10) and the reference gene annotation (M25, GRCm38.p6) to estimate read counts per transcript using the transcript-level mapping algorithm in Salmon (v1.10.0).[6] Differential expression analysis was performed using DESeq2 software (v1.32.0).[7] To calculate the abundances of transcripts, RNA-seq raw datasets were aligned to the mm10 mouse genome using the RNA-seq alignment algorithm in RNA-STAR (v2.7.0).[8] Then, FPKM (Fragments Per Kilobase of exon per Million fragments mapped) was calculated per individual gene, for each sample in Cufflinks (v2.2.1). [9] To avoid producing denominators with a zero value in logarithmic scales (log), a value of 0.1 was added to each gene for each sample. Finally, the median normalized FPKM values were $\log_2$-transformed.

Genomic mapping analysis. ChIP-seq and ATAC-seq analyses were performed using the Galaxy platform (https://usegalaxy.eu).[10] Low-quality reads and adapter sequences were removed from raw FASTQ files using Trim Galore (v0.6.7). Trimmed reads were aligned to the mouse genome (mm10/GRCm38) using Bowtie2 (v2.4.5),[11] and the output BAM files were converted to single-read BED format using bedtools (v2.30.0).[12] For signal visualization in the Integrative Genomics Viewer (IGV; v2.8.12),[13] BED files were converted to BIGWIG format using Galaxy tools. Peak calling was performed using MACS2 (v2.2.9.1),[14] utilizing IgG or no-antibody ChIP-seq datasets as negative controls. For ChIP-seq, we identified 9,719 and 8,620 MACS2 peaks from day 6 WT CREST replicates 1 and 2, respectively. For ATAC-seq, we identified 17,080 and 23,165 highly accessible DNA regions from day 6 WT replicates 1 and 2, respectively. To isolate high-confidence CREST peaks and remove background, we conducted differential binding analysis using DiffBind (v3.12.0).[15] Two WT CREST replicates were compared against two CREST KO replicates ($P < 0.05$), using IgG or no-antibody ChIP-seq datasets as negative controls in DiffBind. If two CREST peak midpoints resided within 2.0 kb of each other, they

were merged into a single "CREST-bound region" by retaining the peak with the more significant *P*-value. Finally, genomic regions within 3 kb of the nearest transcription start site (TSS) of mm10 mRNA genes were classified as promoters (*n* = 10,958 regions), whereas regions >3 kb from the nearest TSS were classified as distal *cis*-regulatory regions. Because CREST is a BAF subunit involved in chromatin accessibility, we restricted our ChIP-seq analysis to highly accessible DNA regions. Of the 14,373 highly accessible distal regions identified on day 6, we defined 4,221 as CREST-bound (overlapping with CREST peaks within 2.0 kb) and 10,152 as CREST-unbound (located >2.0 kb from CREST peaks). All 14,373 highly accessible distal *cis*-regulatory regions are detailed in Supplementary Data 8.

Hi-C analysis. The sequencing output from Hi-C libraries was mapped to the reference genome (mm10) using Burrows-Wheeler Aligner (BWA-MEM; v0.7.17).[16] Chimeric reads spanning multiple sites of the genome were filtered out. The reads with poor mapping quality (MAPQ < 10) and putative self-ligated reads (genomic distance < 5kb) were discarded. Potential PCR duplicates were marked using MarkDuplicates (Picard; v2.6.0, https://broadinstitute.github.io/picard/). The Hi-C contact matrices were Knight-Ruiz normalized and converted into .hic file format by using the juicer (github.com/aidenlab/juicer).[17] Bin-wise quantile normalization was conducted as described [18] to remove unwanted between-sample variations. The directionality index (DI) scores were systematically identified based on normalized 40kb resolution Hi-C contact maps with DomainCaller (v1.0)[19] in a 1 Mb window.

To investigate altered chromatin contacts between differentially expressed genes (DEGs) and CREST-bound accessible regions, we collected the normalized 40 kb resolution bin-pairs linked to all DEGs (at the transcription start site) and CREST-bound accessible regions (at the summit) within a 2 Mb window for individual day 6 WT and day 6 CREST KO Hi-C samples. The contacts were then used as input to LIMMA (v3.50.3).[20] The contacts were considered differential based on a threshold of difference in normalized Hi-C contact > 100 and *P* < 0.01. In order to construct a genome-wide, long-range gene-to-enhancer *cis*-regulation network, we applied the activity-by-contact (ABC) model. [21] The ABC model quantitatively estimated the effect of an accessible chromatin region on target gene expression by combining the *cis*-regulatory activity and chromatin contact frequency. The ABC scores for all *cis*-regulatory elements to gene relationships within the 2 Mb window were computed at 10 kb resolution. The *cis*-regulatory activities were subjected to quantile normalization to match the reference genome, and the estimation was conducted only on the expressed genes. The promoter-centered connections with an ABC score greater than 0.1 were defined as significant interactions.

Gene ontology analysis. Gene ontology (GO) analysis was performed as described by Jaura et al., 2022.[1] Gene ontology terms enriched in CREST-bound and -unbound regions were identified using the Gene Ontology Enrichment Analysis and Visualization (GOrilla) system.[22] The Reduce and Visualize Gene Ontology (REViGO) software was then used with a 0.7 SimRel similarity score based on Resnik's and Lin's similarity measures to obtain summarized, non-redundant gene ontology terms.[23] Reported in the figures are the top seven non-redundant GO terms for biological processes. To define GO target genes of ChIP-seq and ATAC-seq peaks, we assigned ChIP-seq and ATAC-seq intragenic peaks to the closest TSS of mm10 mRNA genes (*n* = 19,980 genes). All mm10 annotated protein-coding genes were used as background for ChIP-seq and ATAC-seq GO analyses. All expressed mRNA genes were used as a background gene set for RNA-seq GO analyses. All GO target genes and background genes are reported in Supplementary Data 5.

Box plot analysis. In **Fig. 2c**, the box and whisker plots of Log_2 FPKM over median values, data were filtered to include the mRNA genes significantly ($P < 0.05$) downregulated or upregulated in day 6 *CREST* ^{KO} cells compared to day 6 WT mature motor neurons. Therefore, data for the non-differentially expressed or not expressed genes were not included. In the box and whisker plots, the lower (1st) and upper (3rd) quartiles are represented by the ends of the box. Therefore, the box spans the interquartile interval. The line inside the box marks the median. 1.5x the interquartile range is shown by the two lines extending outside the box (whiskers). The box plot was generated using KaleidaGraph 5.0.1 (Synergy Software, www.synergy.com).

Statistical analyses. For the comparison of RNA expression Log_2 (FPKM+0.1) between WT and KO cells (**Fig. 2c**, Supplementary Fig. 4d, 16), *P*-values were calculated using a two-tailed paired t-test. For comparisons involving RNA expression fold-change (**Fig. 6b**, Supplementary Fig. 15), RNA expression in FPKM (Supplementary Fig. 3, 7c, 17), distance between distal regions and TSSs (Supplementary Fig. 6a), and ChIP-seq intensities between datasets (Supplementary Fig. 7a, 13), *P*-values were calculated using a two-tailed unpaired Student's t-test assuming equal variance. In **Fig. 4d**, differences in gene expression were evaluated using a two-sided Wilcoxon rank-sum test. In **Fig. 5c** and Supplementary Fig. 6d, to compare all possible pairs of means while controlling for the experiment-wise error rate, statistical differences were assessed using a one-way analysis of variance (ANOVA) followed by a post hoc Tukey's Honestly Significant Difference (HSD) test using the Real Statistics Resource Pack (https://real-statistics.com). Complete statistical details for each group, including means, variances, *F*-values, and *P*-values, are provided in Supplementary Data 10.

Supplementary References

1 Jaura, R. *et al.* Extended intergenic DNA contributes to neuron-specific expression of neighboring genes in the mammalian nervous system. *Nat Commun* **13**, 2733 (2022). https://doi.org:10.1038/s41467-022-30192-z

2 Ran, F. A. *et al.* Genome engineering using the CRISPR-Cas9 system. *Nat Protoc* **8**, 2281-2308 (2013). https://doi.org:10.1038/nprot.2013.143

3 Rhee, H. S. *et al.* Expression of Terminal Effector Genes in Mammalian Neurons Is Maintained by a Dynamic Relay of Transient Enhancers. *Neuron* **92**, 1252-1265 (2016). https://doi.org:10.1016/j.neuron.2016.11.037

4 McCarthy, R. L. *et al.* Diverse heterochromatin-associated proteins repress distinct classes of genes and repetitive elements. *Nat Cell Biol* **23**, 905-914 (2021). https://doi.org:10.1038/s41556-021-00725-7

5 Rao, S. S. *et al.* A 3D map of the human genome at kilobase resolution reveals principles of chromatin looping. *Cell* **159**, 1665-1680 (2014). https://doi.org:10.1016/j.cell.2014.11.021

6 Patro, R., Duggal, G., Love, M. I., Irizarry, R. A. & Kingsford, C. Salmon provides fast and bias-aware quantification of transcript expression. *Nat Methods* **14**, 417-419 (2017). https://doi.org:10.1038/nmeth.4197

7 Love, M. I., Huber, W. & Anders, S. Moderated estimation of fold change and dispersion for RNA-seq data with DESeq2. *Genome Biol* **15**, 550 (2014). https://doi.org:10.1186/s13059-014-0550-8

8 Dobin, A. *et al.* STAR: ultrafast universal RNA-seq aligner. *Bioinformatics* **29**, 15-21 (2013). https://doi.org:10.1093/bioinformatics/bts635

9 Trapnell, C. *et al.* Transcript assembly and quantification by RNA-Seq reveals unannotated transcripts and isoform switching during cell differentiation. *Nat Biotechnol* **28**, 511-515 (2010). https://doi.org:10.1038/nbt.1621

10 Afgan, E. *et al.* The Galaxy platform for accessible, reproducible and collaborative biomedical analyses: 2018 update. *Nucleic Acids Res* **46**, W537-W544 (2018). https://doi.org:10.1093/nar/gky379

11 Langmead, B. & Salzberg, S. L. Fast gapped-read alignment with Bowtie 2. *Nat Methods* **9**, 357-359 (2012). https://doi.org:10.1038/nmeth.1923

12 Quinlan, A. R. & Hall, I. M. BEDTools: a flexible suite of utilities for comparing genomic features. *Bioinformatics* **26**, 841-842 (2010). https://doi.org:10.1093/bioinformatics/btq033

13 Robinson, J. T. *et al.* Integrative genomics viewer. *Nat Biotechnol* **29**, 24-26 (2011). https://doi.org:10.1038/nbt.1754

14 Zhang, Y. *et al.* Model-based analysis of ChIP-Seq (MACS). *Genome Biol* **9**, R137 (2008). https://doi.org:10.1186/gb-2008-9-9-r137

15 Ross-Innes, C. S. *et al.* Differential oestrogen receptor binding is associated with clinical outcome in breast cancer. *Nature* **481**, 389-393 (2012). https://doi.org:10.1038/nature10730

16 Li, H. & Durbin, R. Fast and accurate long-read alignment with Burrows-Wheeler transform. *Bioinformatics* **26**, 589-595 (2010). https://doi.org:10.1093/bioinformatics/btp698

17 Durand, N. C. *et al.* Juicer Provides a One-Click System for Analyzing Loop-Resolution Hi-C Experiments. *Cell Syst* **3**, 95-98 (2016). https://doi.org:10.1016/j.cels.2016.07.002

18 Fletez-Brant, K., Qiu, Y., Gorkin, D. U., Hu, M. & Hansen, K. D. Removing unwanted variation between samples in Hi-C experiments. *Brief Bioinform* **25** (2024). https://doi.org:10.1093/bib/bbae217

19 Dixon, J. R. *et al.* Topological domains in mammalian genomes identified by analysis of chromatin interactions. *Nature* **485**, 376-380 (2012). https://doi.org:10.1038/nature11082

20 Ritchie, M. E. *et al.* limma powers differential expression analyses for RNA-sequencing and microarray studies. *Nucleic Acids Res* **43**, e47 (2015). https://doi.org:10.1093/nar/gkv007

21 Fulco, C. P. *et al.* Activity-by-contact model of enhancer-promoter regulation from thousands of CRISPR perturbations. *Nat Genet* **51**, 1664-1669 (2019). https://doi.org:10.1038/s41588-019-0538-0

22 Eden, E., Navon, R., Steinfeld, I., Lipson, D. & Yakhini, Z. GOrilla: a tool for discovery and visualization of enriched GO terms in ranked gene lists. *BMC Bioinformatics* **10**, 48 (2009). https://doi.org:10.1186/1471-2105-10-48

23 Supek, F., Bosnjak, M., Skunca, N. & Smuc, T. REVIGO summarizes and visualizes long lists of gene ontology terms. *PLoS One* **6**, e21800 (2011). https://doi.org:10.1371/journal.pone.0021800
