# Supplementary material for: CREST functions as a molecular bridge between neuron-specific enhancers and epigenetic regulators in mature motor neurons
Source: Nat Commun. 2026 Oct 10;17:11520. doi: 10.1038/s41467-026-78396-x (PMC13745572; doi:10.1038/s41467-026-78396-x)
Supplement: Supplementary file 2 [file 41467_2026_78396_MOESM2_ESM.pdf]

nature portfolio

Peer Review File

CREST functions as a molecular bridge between neuron-specific enhancers and epigenetic regulators in mature motor neurons

Corresponding Author: Professor Ho Sung Rhee

This file contains all reviewer reports in order by version, followed by all author rebuttals in order by version.

Version 0:

Reviewer comments:

Reviewer #1

(Remarks to the Author)
Montanera et al. present a compelling study uncovering a non-enzymatic role for the BAF subunit CREST in motor neuron differentiation. Through an integrated multi-omics approach—including ChIP-seq, ATAC-seq, Hi-C/ABC modeling, RNA-seq, and CRISPR-mediated enhancer deletions—the authors demonstrate that CREST binds neuron-specific enhancers co-occupied by Onecut1 and Ngn2, recruits CBP to catalyze H3K27ac deposition, and activates key motor neuron genes via long-range chromatin looping. Loss of CREST disrupts the expression of neuronal markers and global transcriptional programs, suggesting that CREST serves as an epigenetic “bridge” that links enhancer recognition to transcriptional activation during motor neuron maturation. The data are of high quality and largely convincing, and the study significantly advances our understanding of BAF complex function in neuronal development. However, several key issues remain that merit careful revision, particularly regarding mechanistic interpretations and translational/ in vivo relevance.

Major comments)

1. Throughout the text, the authors imply that CREST may be involved in remodeling chromatin accessibility or mediating the formation of new chromatin loops. However, the data show that CREST deletion does not reduce chromatin accessibility or disrupt chromatin looping. The phrasing should be revised to reflect this more accurately. For example: “CREST activates neuronal gene expression through established long-range chromatin loops.”
2. While the study is presented in the context of motor neuron development and potential ALS relevance, its translational implications are currently limited. Including additional data from human systems—such as CREST-dependent transcriptional or epigenetic profiling in iPSC-derived motor neurons, or analysis of public ALS datasets—would strengthen the clinical relevance of the findings.
3. The conclusion that CREST knockout disrupts BAF complex assembly is based solely on mRNA expression changes of BAF subunits. Additional biochemical validation (e.g., co-immunoprecipitation or native complex profiling) is needed to support this claim.
4. In Figure 2c, the authors observe that the average expression levels of up- or downregulated genes in Day 6 CREST KO neurons are closer to those of Day 5 WT nascent neurons than Day 4 WT progenitors. However, concluding that CREST KO neurons resemble WT nascent neurons based only on averaged gene expression is premature. Additional validation—such as cell morphology, marker gene expression, and functional assessments—is needed to support this interpretation.
5. In lines 237–240, the authors suggest that p300 is likely not associated with CREST, as it is enriched in both CREST-bound and unbound regions. Figure 7 also suggests that p300 acts mainly at CREST-unbound enhancers. However, H3K27ac, which also shows enrichment in both CREST-bound and unbound regions, is downregulated in CREST KO samples (Figure 3b), indicating functional association. The authors should assess whether p300 enrichment at CREST-bound enhancers is altered in CREST KO neurons to evaluate a possible regulatory relationship.
6. In Figure 6b, it remains unclear whether enhancer deletions result in observable effects on neuronal differentiation or maturation. Clarifying whether these manipulations induce phenotypic consequences would strengthen the interpretation of enhancer function.

Minor comments)
7. In Figure 2c, the authors used a two-sample t-test based on averaged gene expression values per gene set. However, the

aim is to test differences in expression at the individual gene level. A paired t-test would be more appropriate, as it accounts for the matched structure of the data within each gene set.
8. In Figure 5c, comparisons across four time points require a formal statistical framework, such as one-way ANOVA followed by appropriate post-hoc tests. Please update the figure legend and Methods section to specify the statistical analysis, including F-values and p-values, and clearly annotate significance in the figure.
9. In the legend of Figure 4e, correct the statement "a $\log_2$ fold-change < 1" to "a $\log_2$ fold-change < −1" for accuracy.

Reviewer #2

(Remarks to the Author)
This manuscript aims to investigate how CREST, a non-catalytic subunit of the BAF chromatin remodeling complex, regulates motor neuron differentiation. The authors demonstrate the interplay between CREST, the CBP histone acetyltransferase, the transcription factors Onecut1 and Ngn2, and the Brm ATP-dependent chromatin remodeling enzyme. They creatively propose a strategy to jointly analyze ATAC and ChIP signals by grouping DNA regions according to their binding and accessibility status. The complementarity of the various techniques used provides strong experimental support for their study. However, the quality of the manuscript could be further improved in the following aspects.

1- Line 102: The marker Map2 was used to identify mature motor neurons at day 6, which was the primary time point used throughout the study. It would be important to clarify the onset of Map2 expression during the differentiation process, specifically whether it is detectable as early as day 4 or day 5.
2- Line 111: CRISPR-Cas9–mediated genome editing was used to generate the CREST knockout cell line. However, it is unclear whether the control cell line was subjected to the same CRISPR process (without CREST mutation).
3- Line 113: In Fig. 1c, the western blot was performed on cells from day 2 and day 6. It would be interesting to examine CREST expression at day 4, either by western blot or immunostaining.
4- Line 115: In Fig. 1d, immunostaining shows the expression of Hb9 and Isl1 under WT and KO conditions. A higher magnification would allow for a better assessment of the signal reduction. Moreover, quantification of the proportion of positive cells or of signal intensity would more robustly support the claim that "These results showed that CREST knockout inhibited the differentiation of ES cells into motor neurons."
5- Line 127: The experimental setup does not allow for the evaluation of CREST's effect on the maintenance of cell identity. The conclusion should instead emphasize the impact of CREST on cell specification and differentiation.
6- Sequencing Data: For ChIP-seq and ATAC-seq experiments, technical replicates were performed, which strengthens the reliability of the data. This should be more clearly stated in the Methods section. Additionally, a comparison between replicates would be informative (e.g., the number of overlapping regions). Using only the common regions between replicates could have helped eliminate potential non-specific peaks, rather than analyzing each replicate separately.
7- CHIP-seq Data: It is unclear whether a non-ChIP sample (input) was used as a control for the MACS2 analysis.
8- ATAC-seq: Genomic regions were classified as promoters if they were located within 3 kb of the TSS. For greater precision, it would be beneficial to compare the identified peak regions with annotated known promoter regions.
9- Line 207: It was indicated that 29.4% (4221 out of 14,373) of highly accessible distal cis-regulatory regions are occupied by CREST, meaning that 73% (4221 out of 5726) of CREST-bound regions are highly accessible. The analysis of the 27% of regions that are bound but not accessible could provide additional insights. It would be useful to explore whether these regions are associated with genes that are not expressed.
10- Line 240: Using ChIP-seq, the authors show that CBP binding is decreased in CREST-KO cells. Since this decrease appears to be genome-wide, it would be advisable to check CBP expression levels to ensure that the absence of CREST does not affect CBP expression.
9- Line 268: The Hi-C method, followed by ABC analysis, enabled the authors to assign distal regions to their cognate promoters, thereby directly identifying the target genes of CREST. Including the number of target genes would further strengthen the findings. Additionally, earlier in the study, a list of bound genes was established by associating peaks with the closest gene. A comparison between these two lists would provide a clearer perspective on the depth of the analysis.
10- Line 271: A comparison between CREST-bound genes and DEGs is presented, but no numbers are provided. Including the numbers and proportions of direct CREST targets would further strengthen the analysis.
11- Hi-C: ChIP-seq data show that CREST primarily binds to distal regions (enhancers). It would be useful to examine whether the few bound promoters correlate with their cognate enhancers. Specifically, it would be informative to explore whether the presence of CREST at promoters reflects a loop between enhancer and promoter. Additionally, investigating whether genes with CREST binding at promoters are particularly accessible or expressed could provide further insights.
12- Line 491: H3K4me1-ChIP data are mentioned but not presented. Including the H3K4me1 data would provide valuable additional context.

Reviewer #3

(Remarks to the Author)
I co-reviewed this manuscript with one of the reviewers who provided the listed reports. This is part of the Nature Communications initiative to facilitate training in peer review and to provide appropriate recognition for Early Career Researchers who co-review manuscripts.

Reviewer #4

(Remarks to the Author)
Summary

Montanera et. al., investigate the role of the neuron-specific BAF complex subunit CREST in motor neuron (MN) differentiation using a mESC-derived MN model and a suite of chromatin profiling tools including RNA-seq, ChIP-seq, ATAC-seq, and Hi-C. Their findings show that CREST does not mediate long-range chromatin interactions but instead facilitates CBP-mediated enhancer activation through acetylation at H3K27. These CREST-bound enhancers are enriched for MN transcription factor motifs, specifically Onecut1 and Ngn2, and show selective co-occupancy with Brm but not Brg1. This suggests a possible mechanism for cell type-specific BAF subunit recruitment.

Overall, the manuscript is conceptually compelling and provides valuable insight into how neuron-specific chromatin regulators establish subtype-specific gene programs. The data supports a model in which transcriptional and chromatin remodeling machinery recruits CREST-CBP to motor neuron enhancers for deposition of activating histone modifications. Addressing key experimental gaps, particularly around model validation, knockout specificity, and the disease relevance of a CREST KO model, would help solidify the conclusions and broaden the impact of the study.

Major Comments

1.Validation and characterization of cell models

a.The authors should provide more robust evidence that their day 6 ES-derived cultures contain fully mature MNs. While the chosen RNA-seq markers and MAP2 staining are supportive, Map2 is not specific to motor neurons. Consider including additional markers such as ChAT, SMI32, or even electrophysiological profiles, or provide further rational for the current choices

b.The manuscript does not address the purity of MN cultures, which is critical given the potential for signal dilution or contamination in bulk omics assays. Were any FACS-based purifications or single-cell validations performed?

c.The IHC in Figure 1 shows persistent CREST signal in the KO line. This contradicts the western blot and raises concerns about knockout efficiency. Please clarify whether this is due to mosaic KO, antibody background, or something else.

d.The claim that CREST KO results in reduced Hb9 and Isl1 expression (lines 115-117) should be supported by quantification, as the images are not conclusive.

e.The analysis stops at day 6. Later-stage timepoints would clarify whether CREST KO fully prevents MN differentiation preserving a naive state, or just delays MN differentiation.

2.Relevance to ALS and CREST mutations

a.The current study employs a full knockout, yet ALS-associated CREST mutations are heterozygous with one allele fully intact and the other containing a Q388X truncation of the C-terminal region required for CBP interaction. Comparing a CREST +/- or Q388X mutant line as in Cheng et al, 2019, would greatly enhance disease relevance.

b.Only two of the three citations in line 65 identify CREST mutations in ALS patients. Moreover, the known mutations have been identified in just a few patients. Please revise the language around the disease relevance to avoid overstating the findings.

3.Acetyltransferase specificity

a.The manuscript only investigates CBP/p300 involvement. Are these the only acetyltransferases expressed in these neurons? If others are present, e.g. Tip60, please justify why they are not examined or discuss their potential roles.

4.CREST ChIP signal in KO cells

a.The authors should explain the presence of CREST ChIP signal in KO cells. Is this background noise, non-specific antibody binding, or incomplete knockout?

5.Evidence for direct protein interactions

a.Many mechanistic claims rely on co-occupancy in ChIP-seq. Without biochemical validation (co-IP, PLA, mass spec, etc.) the proposed CREST-BRM-TF complexes remain speculative. Consider changing the language or incorporating direct interaction data if possible.

Minor Comments

•Line 330: “CREST-bound regions become accessible” implies causality. Rephrase to clarify that CREST correlates with accessibility but is not directly responsible for it.

•Lines 351 & 498: The implication that Brg1 is not involved in neuronal BAF complexes is misleading. Clarify that Brg1 may play roles in other neuronal contexts or subtypes but not in these.

•Lines 396-399: This section reads like a laundry list. Please rewrite to aid in flow and clarity

•Lines 489-490: Claims about Brm-CREST recruitment by Onecut1 and Ngn2 are based on motif enrichment, not functional or binding evidence. Please adjust the language to reflect this.

•Line 499: Claims CTCF and p300 are non-neuronal transcription factors. P300 is a transcription coactivator, not a transcription factor and CTCF is certainly present in neurons. Please revise.

•Figure 7: Shows multiple BAF complex cartoons in the same protein complex.

Contributions: Remove placeholder text “will be added after reviews from co-authors”

Version 1:

Reviewer comments:

Reviewer #1

(Remarks to the Author)

The authors have substantially improved the manuscript and addressed many of my previous concerns. The revised

analyses support the conclusion that CREST functions at neuronal enhancers that are already engaged in long-range chromatin interactions, rather than establishing chromatin accessibility or chromatin looping de novo.

However, I remain concerned about the interpretation of the newly added western blot data for the BAF complex subunits. The reported reductions in BAF170 and BAF53b protein levels are not readily apparent from the presented blots. In particular, the decrease in BAF170 appears quite modest. Quantitative densitometric analyses from multiple biological replicates, together with statistical evaluation, are necessary to support the claim that these proteins are reduced.

Reviewer #2

(Remarks to the Author)
I don't have any further comments. I am satisfied by the authors' responses.

Reviewer #3

(Remarks to the Author)
I co-reviewed this manuscript with one of the reviewers who provided the listed reports. This is part of the Nature Communications initiative to facilitate training in peer review and to provide appropriate recognition for Early Career Researchers who co-review manuscripts.

Reviewer #4

(Remarks to the Author)
The revised manuscript has addressed all the concerns I had.

Version 2:

Reviewer comments:

Reviewer #1

(Remarks to the Author)
The authors have addressed my remaining concern by providing additional quantitative analyses and appropriately revising their interpretation based on the new data. The updated results do not materially affect the overall conclusions of the study. I have no further concerns and support acceptance of the manuscript in its current form.

Open Access This Peer Review File is licensed under a Creative Commons Attribution 4.0 International License, which permits use, sharing, adaptation, distribution and reproduction in any medium or format, as long as you give appropriate credit to the original author(s) and the source, provide a link to the Creative Commons license, and indicate if changes were made.
In cases where reviewers are anonymous, credit should be given to 'Anonymous Referee' and the source.

The images or other third party material in this Peer Review File are included in the article's Creative Commons license, unless indicated otherwise in a credit line to the material. If material is not included in the article's Creative Commons license and your intended use is not permitted by statutory regulation or exceeds the permitted use, you will need to obtain permission directly from the copyright holder.
To view a copy of this license, visit https://creativecommons.org/licenses/by/4.0/

REVIEWER COMMENTS

Reviewer #1 (Remarks to the Author):

Montanera et al. present a compelling study uncovering a non-enzymatic role for the BAF subunit CREST in motor neuron differentiation. Through an integrated multi-omics approach—including ChIP-seq, ATAC-seq, Hi-C/ABC modeling, RNA-seq, and CRISPR-mediated enhancer deletions—the authors demonstrate that CREST binds neuron-specific enhancers co-occupied by Onecut1 and Ngn2, recruits CBP to catalyze H3K27ac deposition, and activates key motor neuron genes via long-range chromatin looping. Loss of CREST disrupts the expression of neuronal markers and global transcriptional programs, suggesting that CREST serves as an epigenetic "bridge" that links enhancer recognition to transcriptional activation during motor neuron maturation. The data are of high quality and largely convincing, and the study significantly advances our understanding of BAF complex function in neuronal development. However, several key issues remain that merit careful revision, particularly regarding mechanistic interpretations and translational/ in vivo relevance.

Major comments

1. Throughout the text, the authors imply that CREST may be involved in remodeling chromatin accessibility or mediating the formation of new chromatin loops. However, the data show that CREST deletion does not reduce chromatin accessibility or disrupt chromatin looping. The phrasing should be revised to reflect this more accurately. For example: "CREST activates neuronal gene expression through established long-range chromatin loops."

Response to Point #1:
We thank the reviewer for suggesting this. We now rephrased this statement. For example, we removed the following statements in the text: "CREST-bound cis-regulatory regions may be associated with their target genes via long-range chromatin interactions." and "CREST activates neurodevelopmental genes through long-range chromatin interactions between CREST-bound distal regions and their target genes." Instead, we revised the text as suggested to reflect this more accurately, like "CREST-bound enhancers modulate neuronal gene expression by utilizing pre-existing long-range chromatin interactions rather than actively reorganizing the genomic architecture" (Revised Line 281).

2. While the study is presented in the context of motor neuron development and potential ALS relevance, its translational implications are currently limited. Including additional data from human systems—such as CREST-dependent transcriptional or epigenetic profiling in iPSC-derived motor neurons, or analysis of public ALS datasets—would strengthen the clinical relevance of the findings.

Response to Point #2:
We appreciate this constructive feedback. Although a previous study by Chen et al. (2019, Translational Neurodegeneration, PMID: 30976389) examined the effects of a CREST mutation in CREST knockout and Q394X knock-in mice using microarray analysis, the dataset was not made publicly available. Although we requested this dataset to the authors, we were unable to obtain it.

As an alternative, we processed RNA-seq datasets generated by Catanese et al. (2021, EMBO Molecular Medicine, PMID: 34125498) to assess the potential relevance of our day 6 *CREST* knockout (KO) cells to ALS-associated mutations. These RNA-seq datasets were from human inducible pluripotent stem cell (iPSC)-derived motor neurons, obtained from ALS patients harboring *C9orf72* mutations (two patients), the most common cause of familial and sporadic ALS, and *FUS*

mutations (two patients), which are responsible for juvenile-onset ALS. Control samples included replicates from three human iPSC lines from one healthy individual and replicates from three CRISPR-corrected iPSC lines derived from a *C9orf72*-mutant individual. We evaluated the gene expression changes driven by our *CREST* KO system compared to those of ALS-associated mutations using gene set enrichment analysis (Supplementary Fig. 4e). We found that the differentially expressed genes in our day 6 KO cells significantly correlated with the transcriptional changes induced by ALS mutations in both the *C9orf72* and *FUS* genes. These results demonstrate that *CREST* KO-induced gene expression changes in our differentiation system are highly relevant to ALS-associated mutations found in human patients.

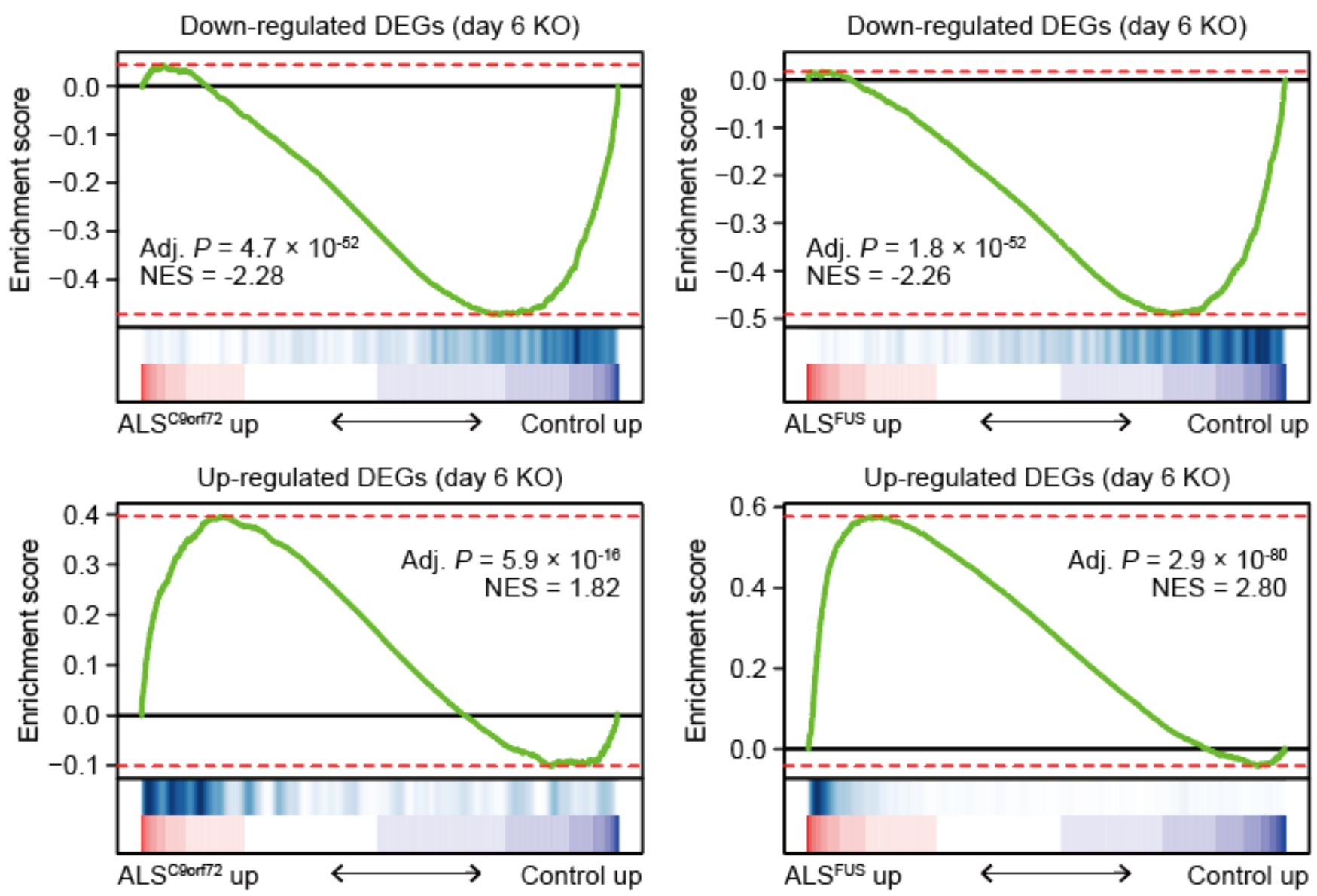

Supplementary Fig. 4e | Analysis of day 6 CREST knockout differentially expressed genes (DEGs) in mRNA-seq data from human iPSC-derived motor neurons with ALS mutations.55 Gene set enrichment analysis (GSEA) plots showing down- and up-regulated genes in day 6 KO cells in the context of ALSC9orf72 mutations (left) and ALSFUS mutations (right) compared with control motor neurons.

3. The conclusion that CREST knockout disrupts BAF complex assembly is based solely on mRNA expression changes of BAF subunits. Additional biochemical validation (e.g., co-immunoprecipitation or native complex profiling) is needed to support this claim.

Response to Point #3:

We thank the reviewer for suggesting additional biochemical validation. To confirm the protein expression levels of BAF subunits upon CREST knockout, we also performed western blotting for three neuronal subunits (BAF170, BAF53b, BRM) and four non-neuronal subunits (ARID1A, BRG1, BAF57, SS18) in day 6 WT and day 6 *CRESK* KO cells (Supplementary Fig. 18a). We found that CREST knockout resulted in a reduction of the protein levels of two neuronal BAF subunits, BAF170 and BAF53b, consistent with the changes in their mRNA levels upon CREST knockout (Supplementary Fig. 17a,b). In contrast, the protein levels of BRM and non-neuronal BAF subunits (ARID1A, BRG1, BAF57, SS18) did not decrease.

Furthermore, we examined whether *CREST* KO affects the protein-protein interactions of these two neuronal BAF subunits, BAF170 and BAF53b, with another neuronal BAF subunit, BRM, using co-immunoprecipitation (Supplementary Fig. 18b). First, we confirmed that BRM interacts with CREST in day 6 WT motor neurons (**Fig. 5h**). Then, we found that the interactions between BRM and BAF170 were not altered by *CREST* KO, and that BAF53b did not interact with BRM in motor neurons. Together, these results suggest that CREST knockout downregulates the expression of

certain neuronal BAF subunits, including BAF170 and BAF53b, but does not disrupt the assembly of these neuronal subunits within the BAF complexes of motor neurons.

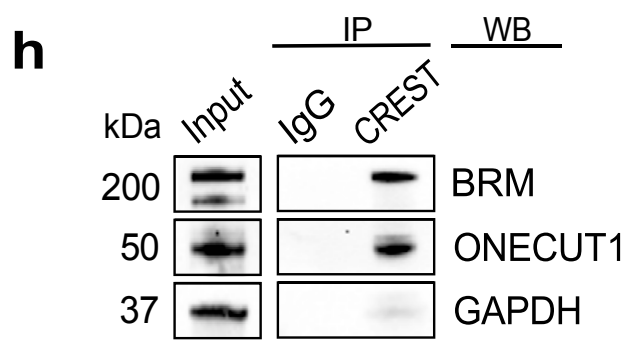

Figure 5h | Biochemical demonstration of the interactions of CREST with BRM and ONECUT1 in day 6 WT motor neurons. Immunoprecipitation (IP) of day 6 cell lysates was performed using an anti-CREST antibody, followed by western blot (WB) analysis for BRM and a negative control (GAPDH). The detected bands correspond to ~200 kDa for BRM, ~50 kDa for ONECUT1, and ~37 kDa for GAPDH. The input consists of 25 µg of nuclear extract; normal IgG serves as a negative control for the IP.

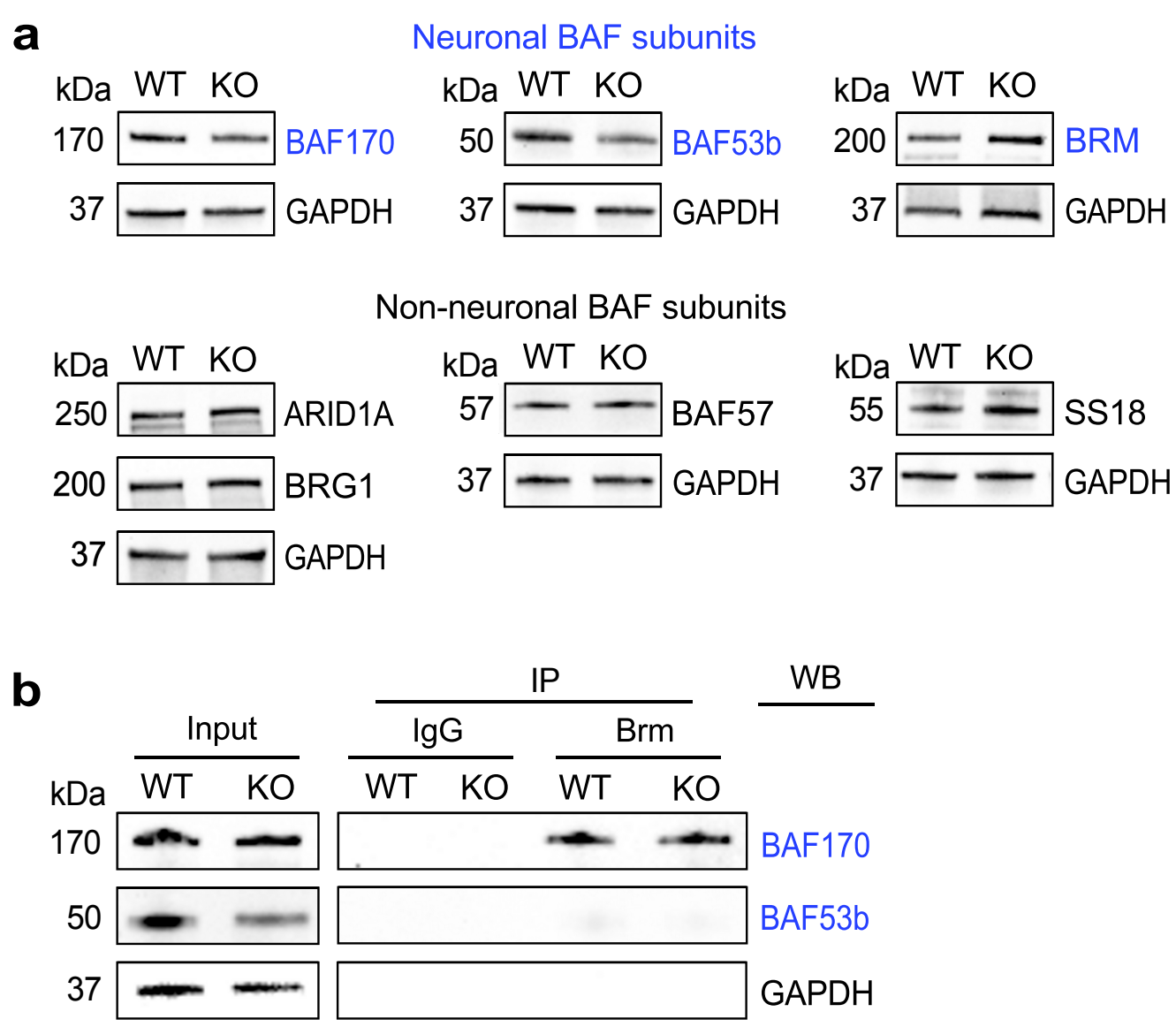

Supplementary Fig. 18 | CREST knockout downregulates the protein levels of specific neuronal BAF subunits in day 6 differentiated cells. **a,** Western blot analysis confirms that the CREST knockout reduces the protein levels of two neuronal BAF subunits, BAF170 and BAF53b, in day 6 differentiated cells. However, it does not affect the protein levels of another neuronal BAF subunit, BRM, or the non-neuronal BAF subunits ARID1A, BRG1, BAF57, and SS18. The detected bands correspond to ~170 kDa for BAF170, ~50 kDa for BAF53b, ~200 kDa for BRM and BRG1, ~250 kDa for Arid1a, ~57 kDa for BAF57, ~55 kDa for SS18, and ~37 kDa for GAPDH (used as a loading control). **b,** Biochemical demonstration of the interactions of BRM with BAF170 and BAF53b in day 6 WT and day 6 KO cells. Immunoprecipitation (IP) of day 6 cell lysates was performed using an anti-BRM antibody, followed by western blot (WB) analysis for BAF170, BAF53b, and GAPDH (used as a negative control). The detected bands correspond to ~170 kDa for BAF170, ~50 kDa for BAF53b, and ~37 kDa for GAPDH. The input consists of 25 µg of nuclear extract; normal IgG served as a negative control for the IP.

4. In Figure 2c, the authors observe that the average expression levels of up- or downregulated genes in Day 6 CREST KO neurons are closer to those of Day 5 WT nascent neurons than Day 4 WT progenitors. However, concluding that CREST KO neurons resemble WT nascent neurons based only on averaged gene expression is premature. Additional validation—such as cell morphology, marker gene expression, and functional assessments—is needed to support this interpretation.

Response to Point #4:

We thank the reviewer for suggesting additional analysis. To address this, we further confirmed that the expression of day 5-specific key marker genes for immature neurons (*Dcx, p35,* and *Astn1*) and

nascent neurons (*Nyap2, Klf7,* and *Dcc*) was similar between day 6 KO and day 5 WT cells (Supplementary Fig. 5).

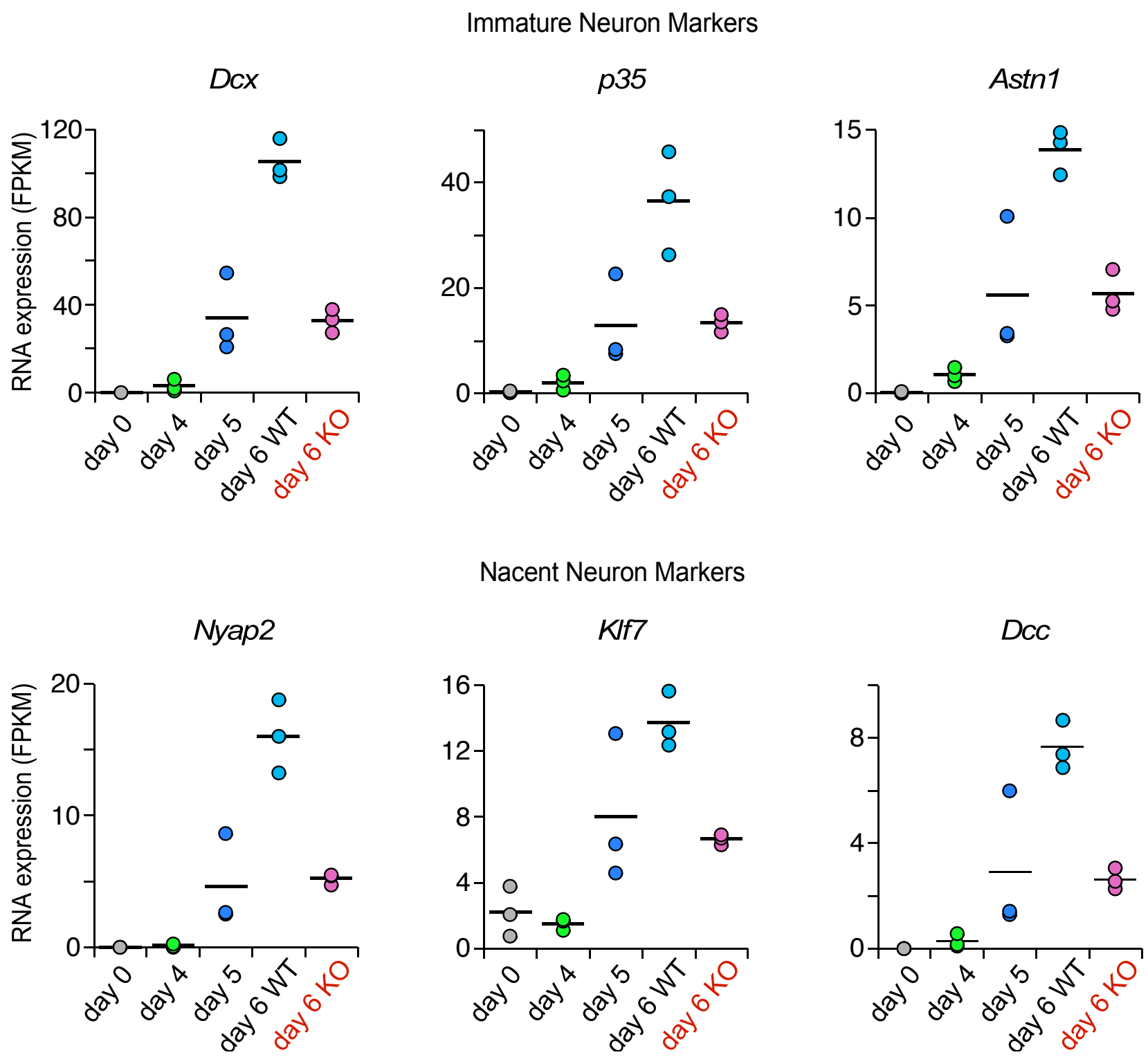

Supplementary Fig. 5 | The expression of key marker genes for immature and nascent neurons was similar between day 6 KO and day 5 WT cells. RNA expression (FPKM) of key marker genes for immature neurons (Dcx, p35, and Astn1) and nascent neurons or developing axon guidance (Nyap2, Klf7, and Dcc), measured by RNA-seq during motor neuron differentiation (day 0 ES cells, day 4 neural progenitors, day 5 nascent motor neurons, day 6 WT mature motor neurons, and day 6 KO cells) from three biological replicates (n = 3) of independent cell differentiations. Lines in the dot plots show the average.

5. In lines 237–240, the authors suggest that p300 is likely not associated with CREST, as it is enriched in both CREST-bound and unbound regions. Figure 7 also suggests that p300 acts mainly at CREST-unbound enhancers. However, H3K27ac, which also shows enrichment in both CREST-bound and unbound regions, is downregulated in CREST KO samples (Figure 3b), indicating functional association. The authors should assess whether p300 enrichment at CREST-bound enhancers is altered in CREST KO neurons to evaluate a possible regulatory relationship.

Response to Point #5:

We thank the reviewer for suggesting this analysis. To determine whether there is a functional association between CREST binding and p300 recruitment, we performed ChIP-seq for p300 in day 6 KO cells. We found that p300 enrichment at both CREST-bound and -unbound enhancers was not changed upon CREST knockout (**Fig. 3b,d**, Supplementary Fig. 8a). This demonstrates that there is no functional association between CREST binding and p300 recruitment. We also observed that H3K27ac levels at CREST-bound enhancers did not completely disappear in day 6 KO cells. These results suggest that p300 may contribute to H3K27ac enrichment at both CREST-bound and -unbound enhancers independently of CREST binding.

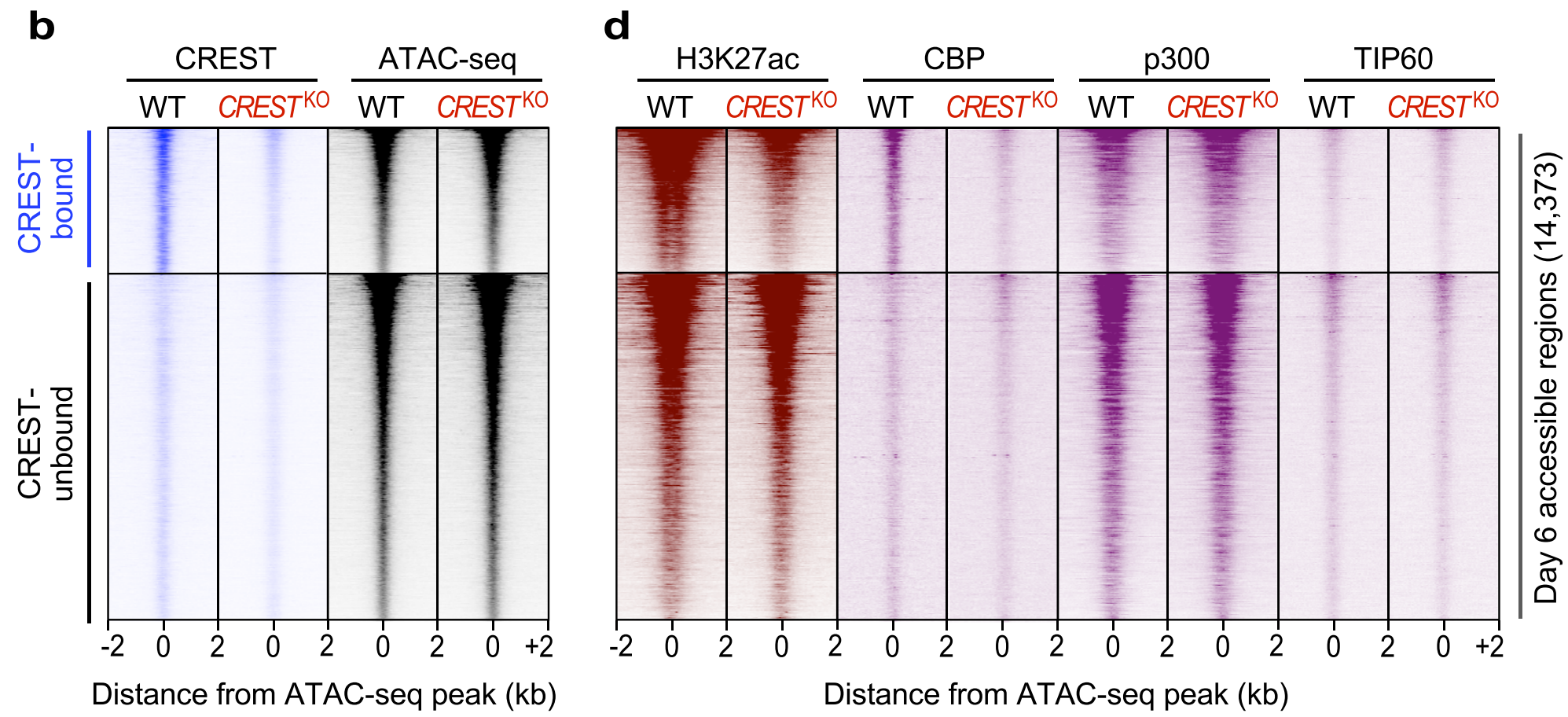

Figure 3 | CREST activates neuron-specific distal *cis*-regulatory regions by recruiting CBP to acetylate histone H3K27. **b,** Genomic mapping of ChIP-seq CREST and ATAC-seq in day 6 WT motor neurons and day 6 KO cells. 14,373 highly accessible distal cis-regulatory regions were identified from ATAC-seq in day 6 WT motor neurons, grouped by CREST-bound (n = 4221) and -unbound distal regions (n = 10,152). All data were plotted relative to the ATAC-seq peak midpoints, sorted, and ordered by ATAC-seq intensity in day 6 WT motor neurons (Supplementary Data 6). **d,** ChIP-seq mapping of H3K27ac and histone acetyltransferases (CBP, p300, and TIP60) in day 6 WT motor neurons and day 6 KO cells at the accessible distal cis-regulatory regions identified in panel (b). All data were plotted relative to 14,373 ATAC-seq peak midpoints in the same order as shown in panel (b).

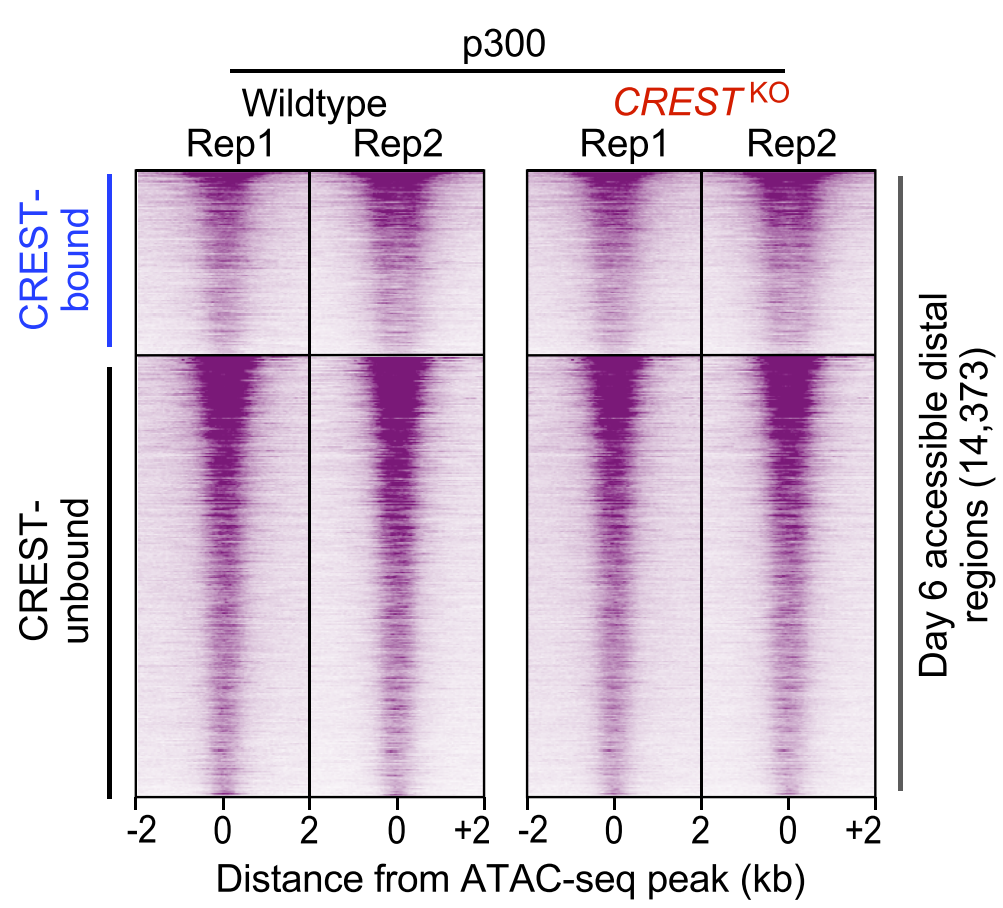

Supplementary Fig. 8a | ChIP-seq mapping of p300 at 4221 CREST-bound and 10,152 CREST-unbound distal cis-regulatory regions in day 6 WT motor neurons and day 6 KO cells, shown in Fig. 3b, d, with two biological replicates of independent differentiations. All data were plotted relative to the ATAC-seq peak midpoints, sorted, and ordered by ATAC-seq intensity in day 6 WT motor neurons.

6. In Figure 6b, it remains unclear whether enhancer deletions result in observable effects on neuronal differentiation or maturation. Clarifying whether these manipulations induce phenotypic consequences would strengthen the interpretation of enhancer function.

Response to Point #6:

We thank the reviewer for suggesting this analysis. To address this, we further determine whether the deletions of CREST-bound enhancers affect neuronal differentiation. To test this, we examined neurite outgrowth in two enhancer-deletion cell lines (*Chrm2* and *Kcna5* enhancer deletions) during motor neuron differentiation (Supplementary Fig. 15b). We observed that the *Chrm2* enhancer deletion impaired axon outgrowth, as visualized by staining for the neuronal marker TUBB3. We also observed that the *Kcna5* enhancer deletion impaired the localization of MAP2, a marker for mature

neurons, to the dendrites. These results confirm that the deletion of these CREST-bound enhancers results in phenotypic changes during neuronal maturation.

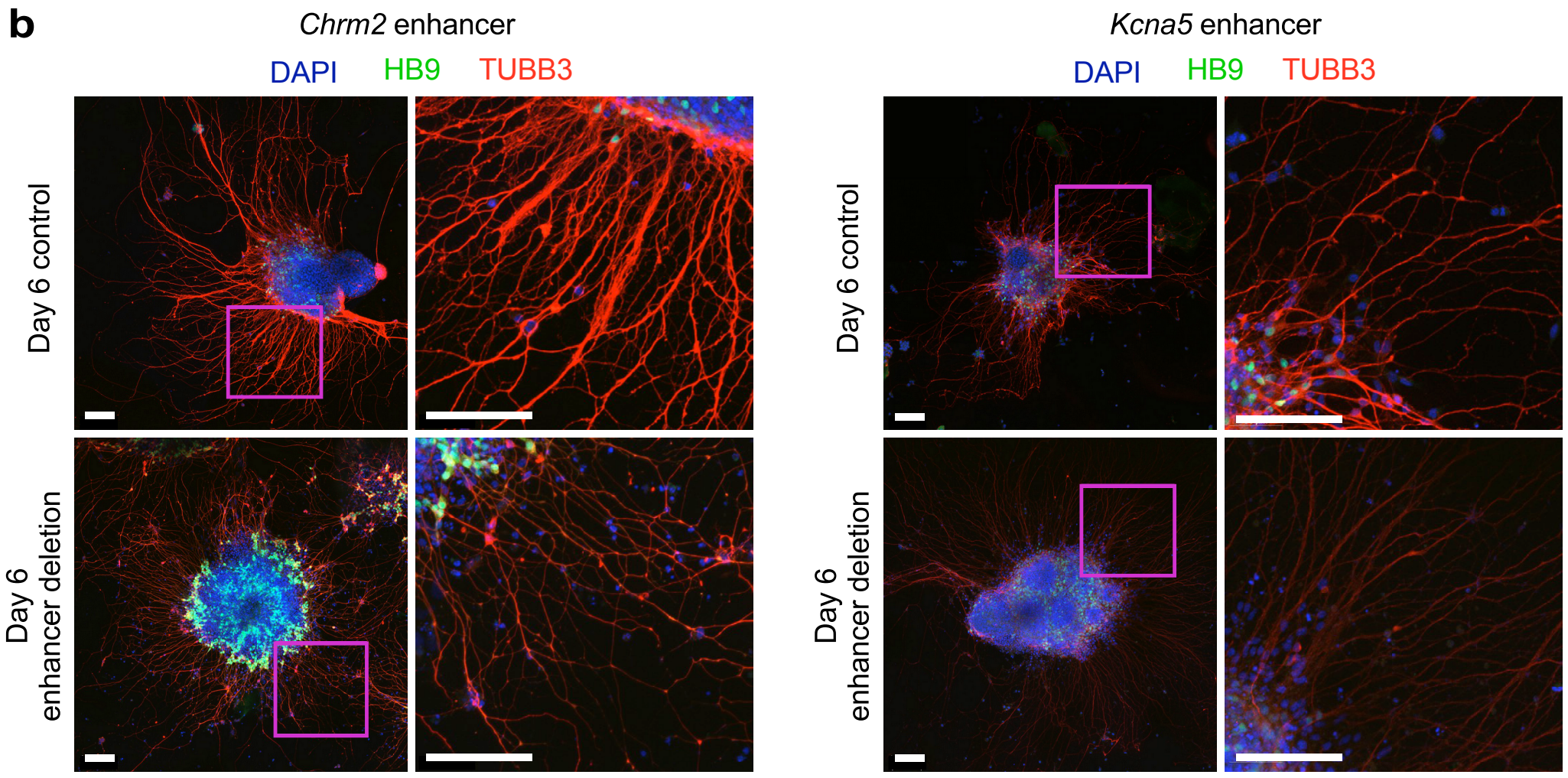

Supplementary Fig. 15b | Immunostaining of differentiated embryoid bodies on laminin-coated surfaces showing the nuclear-specific dye DAPI, the motor neuron-specific marker HB9, the neuronal marker TUBB3 (class III b-tubulin), and the mature neuronal marker MAP2 (microtubule-associated protein 2) in day 6 WT control and the two day 6 enhancer deletion cell lines (Chrm2 and Kcna5 enhancer deletions), as shown in Fig. 6a. Magenta boxes in the left panels indicate the regions at higher magnification on the right. Scale bars represent 100 μm.

Minor comments

7. In Figure 2c, the authors used a two-sample t-test based on averaged gene expression values per gene set. However, the aim is to test differences in expression at the individual gene level. A paired t-test would be more appropriate, as it accounts for the matched structure of the data within each gene set.

Response to Point #7:
We appreciate the reviewer's careful evaluation of our statistical methodology. We agree that because we are evaluating the expression of the exact same gene set across different conditions, a test that accounts for this matched structure is required to assess differences at the individual gene level. Accordingly, we have re-analyzed the data in **Figure 2c**. For pairwise comparisons between two specific states (e.g., Day 6 WT vs. Day 6 KO), we have applied a paired Student's t-test. We have also updated the figure legend, and the Methods section to reflect these more rigorous statistical tests, which have further strengthened our original biological conclusions.

8. In Figure 5c, comparisons across four time points require a formal statistical framework, such as one-way ANOVA followed by appropriate post-hoc tests. Please update the figure legend and Methods section to specify the statistical analysis, including F-values and p-values, and clearly annotate significance in the figure.

Response to Point #8:
We thank the reviewer for suggesting these statistical tests. We conducted one-way analysis of variance (ANOVA; single-factor test) followed by Tukey's post hoc tests for three gene groups (*a1, a2*, and *b1*) in **Fig. 5c**. We also updated the figure legends and Methods section to specify the statistical analysis, including F-values and P-values. Please note that we indicated the exact *P*-values in the

figure instead of using asterisks to show significance or non-significance. All statistical values were reported in Supplementary Data 8.

9. In the legend of Figure 4e, correct the statement “a $\log_2$ fold-change < 1” to “a $\log_2$ fold-change < −1” for accuracy.

Response to Point #9:
We thank the reviewer for this comment. We have now corrected this statement.

Reviewer #2 (Remarks to the Author):

This manuscript aims to investigate how CREST, a non-catalytic subunit of the BAF chromatin remodeling complex, regulates motor neuron differentiation. The authors demonstrate the interplay between CREST, the CBP histone acetyltransferase, the transcription factors Onecut1 and Ngn2, and the Brm ATP-dependent chromatin remodeling enzyme. They creatively propose a strategy to jointly analyze ATAC and ChIP signals by grouping DNA regions according to their binding and accessibility status. The complementarity of the various techniques used provides strong experimental support for their study. However, the quality of the manuscript could be further improved in the following aspects.

1- Line 102: The marker Map2 was used to identify mature motor neurons at day 6, which was the primary time point used throughout the study. It would be important to clarify the onset of Map2 expression during the differentiation process, specifically whether it is detectable as early as day 4 or day 5.

Response to Point #1:
We thank the reviewer for suggesting this experiment. To examine the onset of MAP2 expression during motor neuron differentiation, we performed western blotting for MAP2 across four distinct stages: day 2 (primitive ectoderm), day 4 (neural progenitor), day 5 (nascent motor neuron), and day 6 (mature motor neuron) of the differentiation process. We confirmed that MAP2 protein expression was not detected in day 4 neural progenitors and day 5 nascent motor neurons, but was expressed specifically in day 6 mature motor neurons (**Fig. 1c**).

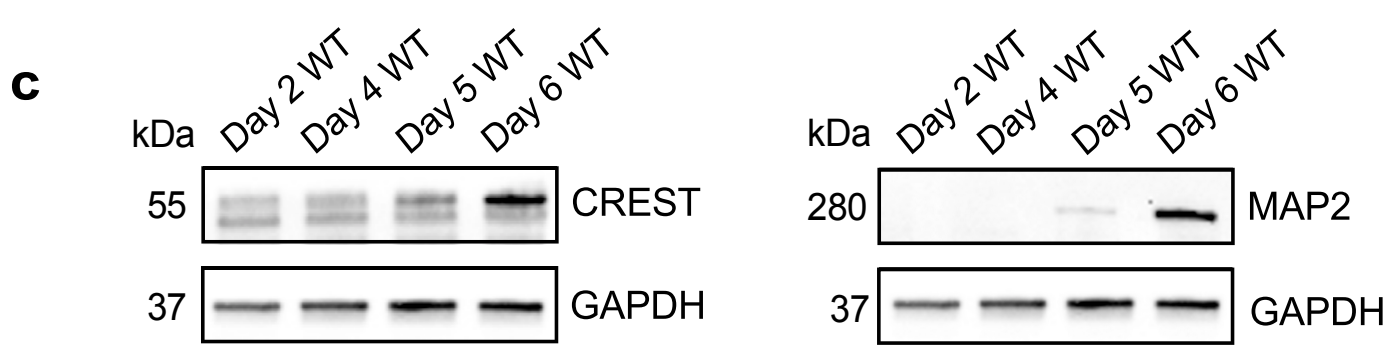

Figure 1c | Western blot analysis confirms that CREST (SS18L1) and the mature neuronal marker MAP2 are detected at ~55 kDa (kilodaltons) and ~280 kDa, respectively, only in day 6 WT motor neurons during differentiation. The housekeeping protein GAPDH (~37 kDa) serves as a loading control.

2- Line 111: CRISPR-Cas9–mediated genome editing was used to generate the CREST knockout cell line. However, it is unclear whether the control cell line was subjected to the same CRISPR process (without CREST mutation).

Response to Point #2:
We thank the reviewer for this valuable comment. Although we missed a mock-edited control mouse embryonic stem (ES) cell line when we initially designed our CRISPR/Cas9 experiments, we assessed whether the CRISPR/Cas9 genome editing process influenced the ES cells' behavior independently of the intended *CREST* knockout. To address this, we confirmed that WT and *CREST* KO ES cell colonies stained at similar levels for the ES cell pluripotency marker alkaline phosphatase (Supplementary Fig. 2b). This demonstrates that the pluripotency of our *CREST* KO ES cells was not compromised by the CRISPR/Cas9-mediated genome editing process.

Furthermore, in this revision, we showed new results that while the expression levels of mature neuronal genes were downregulated in day 6 KO cells, they fully recovered in day 9 KO cells, which exhibit the similar gene expression profile as day 9 WT mature motor neurons (Supplementary Fig. 16). This suggests that *CREST* knockout delays the neuronal maturation process rather than disrupting the overall motor neuron differentiation process. Together, our new results strongly

support that the CRISPR/Cas9 editing process itself did not affect the ES cells' pluripotency or their capacity for motor neuron differentiation.

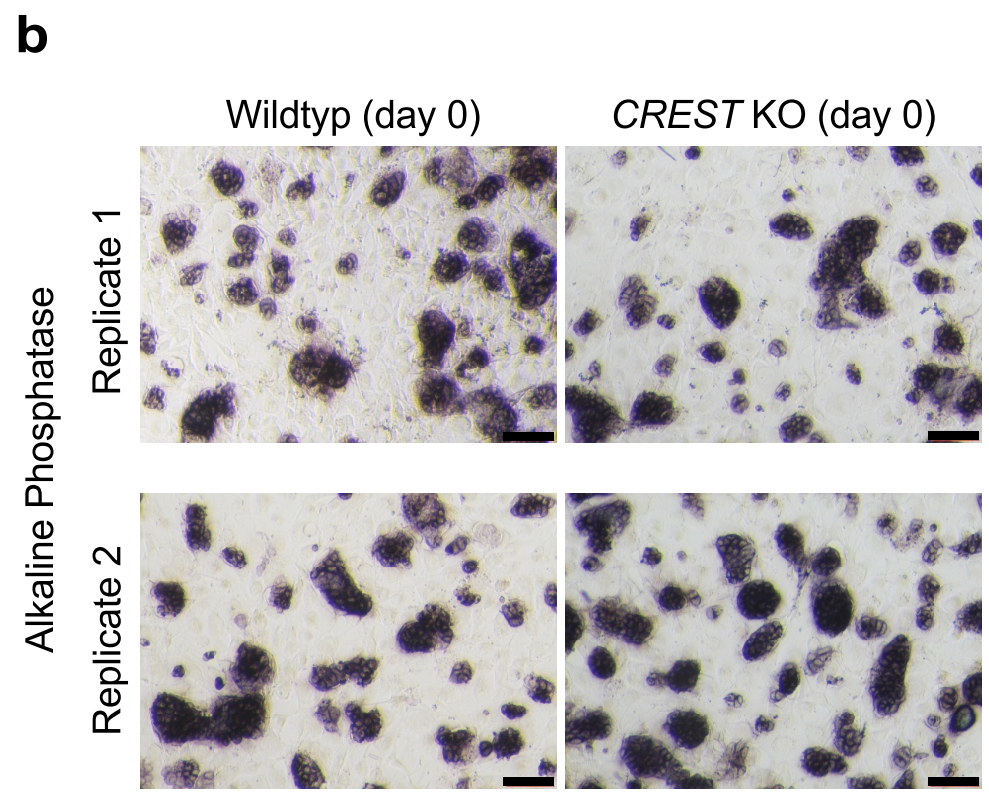

Supplementary Fig. 2b | Day 0 ES cells were stained for the pluripotency marker alkaline phosphatase. Both WT and *CREST* KO ES cells exhibited similar levels of purple staining, indicating comparable ALP activity. Scale bars represent 200 μm.

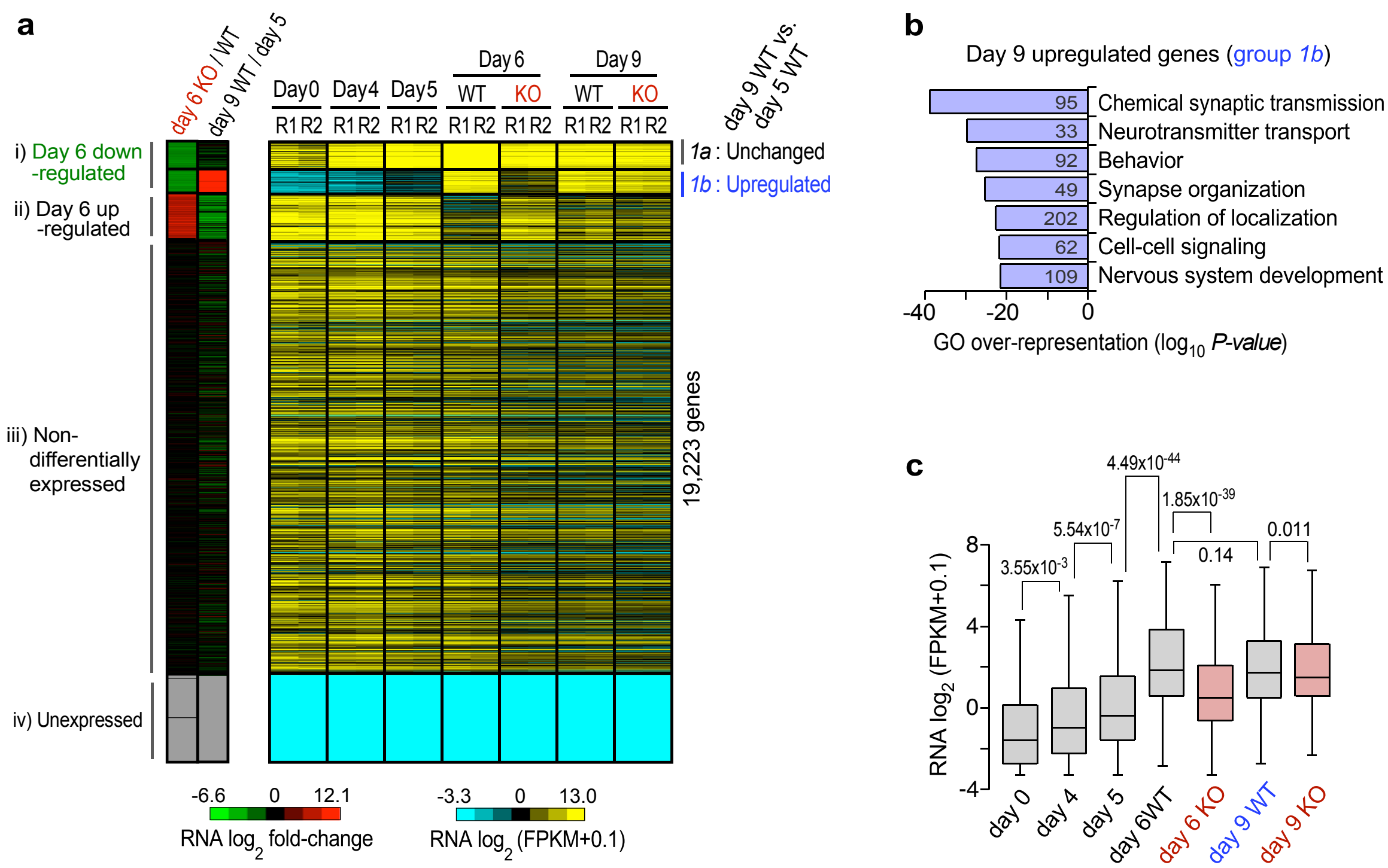

Supplementary Fig. 16 | CREST knockout delays the neuronal maturation, extending the differentiation process from 6 days to 9 days. **a,** Left: Heatmap of RNA expression (log2 fold-changes) of all 19,223 mRNA genes in day 6 KO cells compared with day 6 WT cells. Genes are ordered as displayed in Fig. 2a, except for the Day 6 downregulated genes (Group i), which were further subdivided based on RNA expression changes in day 9 WT cells compared with day 5 WT cells (Group 1a: 879 unchanged or downregulated genes; Group 1b: 735 upregulated genes). Right: Heatmap of RNA expression (log2 FPKM+0.1) of all mRNA genes in days 0, 4, 5, and 6 WT and day 6 KO cells, as well as day 9 WT and day 9 KO cells. FPKM values were adjusted by adding 0.1, median-normalized, log2-transformed, and ordered as displayed on the left. The heatmap includes data from two biological replicates (R1 and R2) derived from independent cell differentiations. **b,** Gene ontology (GO) analysis of 735 upregulated genes (Group 1b) identified in panel (a). The seven most over-represented and non-redundant GO terms are displayed. Numbers within bars represent the count of genes associated with each GO term. **c,** Box plots showing RNA expression (log2 FPKM+0.1) for upregulated 95 chemical synaptic transmission genes (Group 1b, the top GO term genes), identified in panel (b). FPKM values represent averages from two biological replicates, shown in panel

(a). The plots display the median (line within boxes), first-to-third quartiles (bottom and top of boxes, respectively), and 1.5x the interquartile range (whiskers). A two-sided paired t-test.

3- Line 113: In Fig. 1c, the western blot was performed on cells from day 2 and day 6. It would be interesting to examine CREST expression at day 4, either by western blot or immunostaining.

Response to Point #3:
We thank the reviewer for suggesting this experiment. To examine the onset of CREST expression during motor neuron differentiation, we performed western blotting for CREST across four distinct stages: day 2 (primitive ectoderm), day 4 (neural progenitor), day 5 (nascent motor neuron), and day 6 (mature motor neuron) of the differentiation process. We observed that CREST protein expression was not detected in day 2, day 4, or day 5 cells, but was expressed specifically in day 6 mature motor neurons (presented by the upper band at 55 kDa, **Fig. 1c**). The lower protein band near 55 kDa corresponds to SS18, a CREST paralog expressed in non-neuronal cells (**Fig. 1d**). Please see more details regarding the specificity and background of the anti-CREST antibody in Response to **Reviewer 4's Point 1c**.

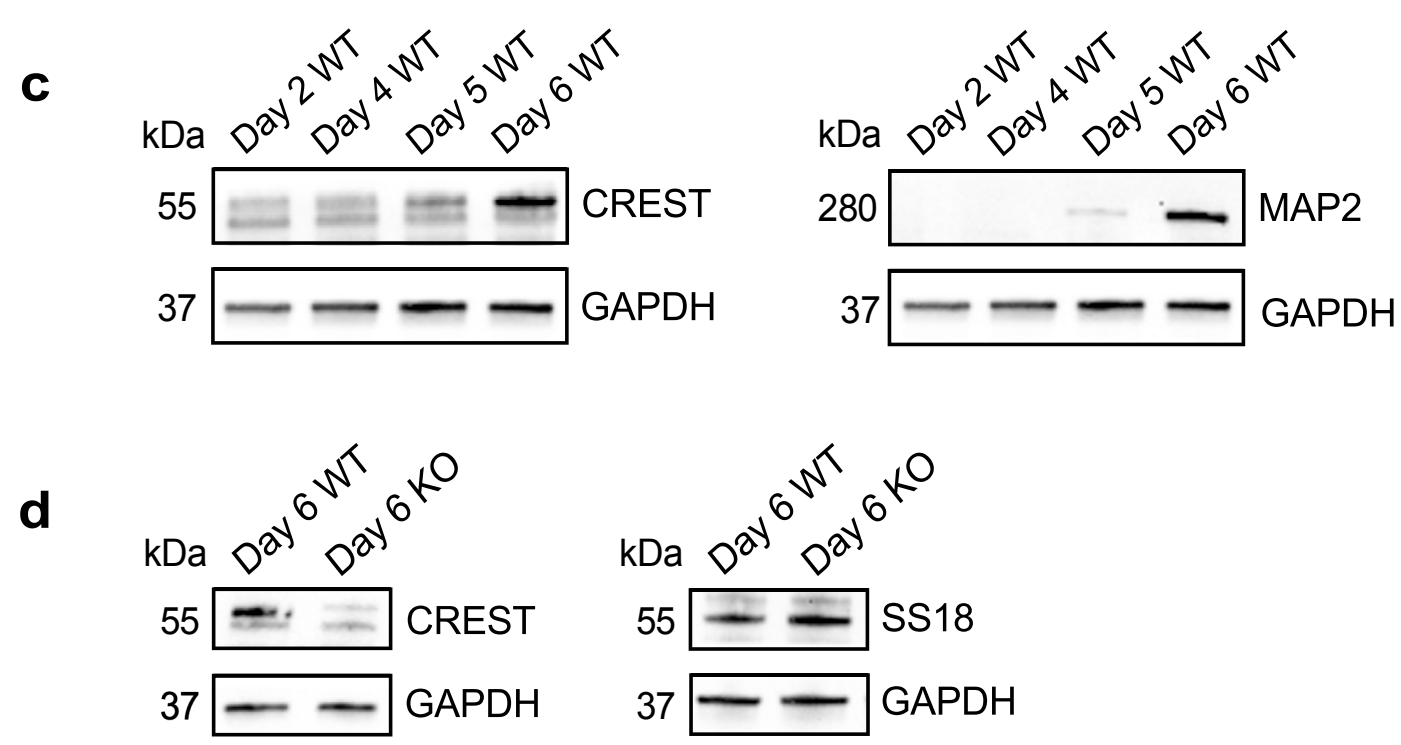

Figure 1 | **c,** Western blot analysis confirms that CREST (SS18L1) and the mature neuronal marker MAP2 are detected at ~55 kDa (kilodaltons) and ~280 kDa, respectively, only in day 6 WT motor neurons during differentiation. The housekeeping protein GAPDH (~37 kDa) serves as a loading control. **d,** Western blot analysis confirms that the CREST knockout (KO), generated via CRISPR-Cas9, eliminates CREST protein expression in day 6 differentiated cells. CREST is detected at ~55 kDa (upper band) in day 6 WT cells but is absent in day 6 KO cells. The CREST paralog, SS18, is detected at ~55 kDa (lower band) in both day 6 WT and day 6 KO cells. Biological replicates are shown in Supplementary Fig. 2c.

4- Line 115: In Fig. 1d, immunostaining shows the expression of Hb9 and Isl1 under WT and KO conditions. A higher magnification would allow for a better assessment of the signal reduction. Moreover, quantification of the proportion of positive cells or of signal intensity would more robustly support the claim that "These results showed that CREST knockout inhibited the differentiation of ES cells into motor neurons."

Response to Point #4:
We thank the reviewer for this comment. We have now included high-magnification immunostaining images of Hb9 and Isl1 expression in day 6 WT and KO cells, which provide better visualization of the reduction in their expression upon CREST knockout (Supplementary Fig. 2e). Additionally, we quantified the proportion of cells positive for the motor neuron markers Hb9 and Isl1 in day 6 WT and KO cells. Our results show that the percentage of Isl1- and Hb9-positive cells decreased by 56.2% and 89.8% upon CREST knockout, respectively (Supplementary Fig. 2d). These findings support our conclusion that CREST knockout inhibits the differentiation of ES cells into motor

neurons.

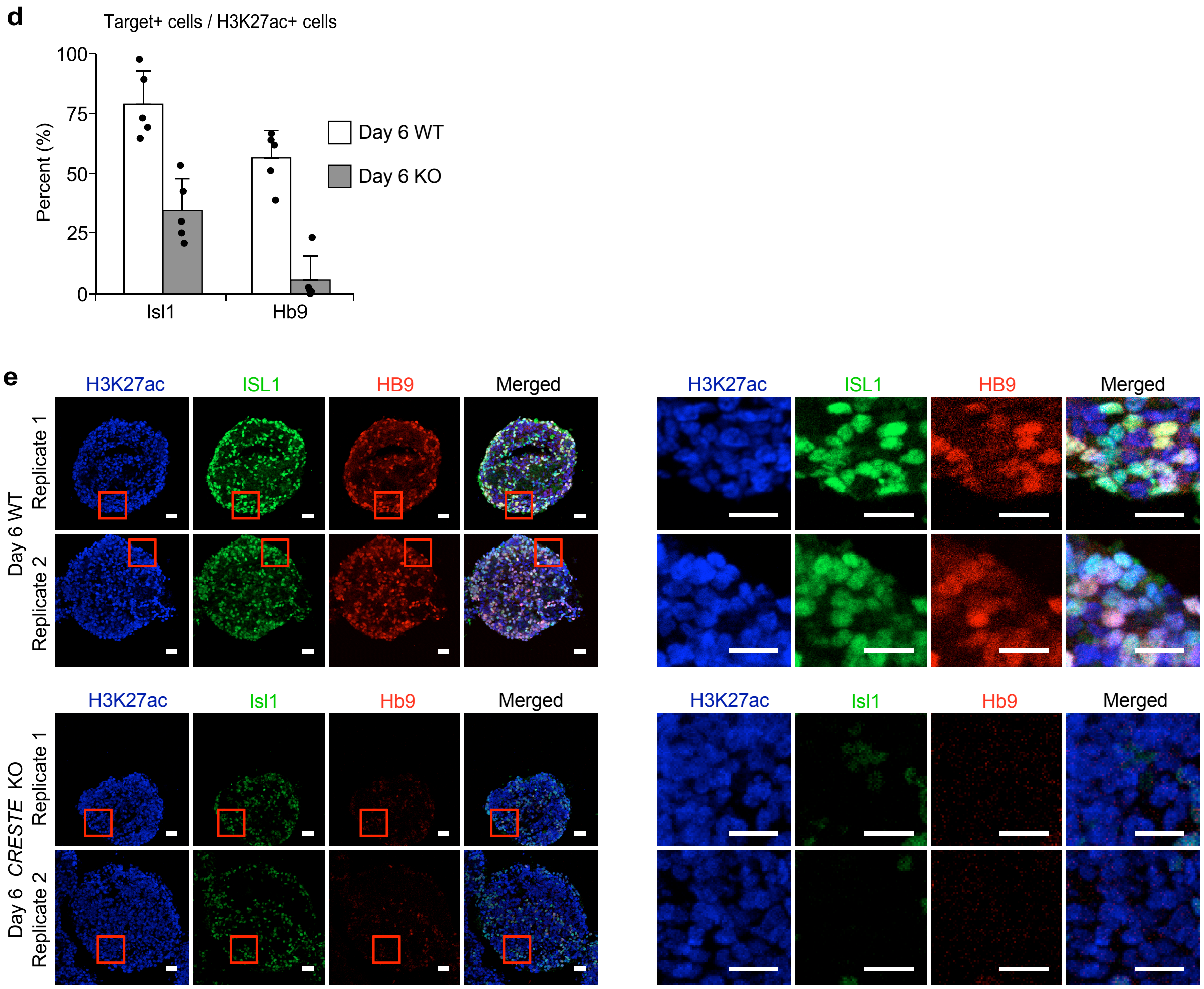

Supplementary Fig. 2 | **d,** Quantification of transcriptionally active (H3K27ac-positive) cells expressing the motor neuron-specific markers ISL1 or HB9 in day 6 WT and day 6 KO cells. Embryoid bodies were immunostained for HB9, ISL1, and H3K27ac, followed by the quantification of immuno-positive cells. Data are presented as mean ± SEM (n = 5 embryoid bodies per target marker). **e,** Immunostaining of differentiated embryoid bodies showing H3K27ac, ISL1, and HB9 in day 6 WT motor neurons and day 6 KO cells. Red boxes in the left panels indicate the regions at higher magnification on the right. Scale bars represent 20 μm.

5- Line 127: The experimental setup does not allow for the evaluation of CREST's effect on the maintenance of cell identity. The conclusion should instead emphasize the impact of CREST on cell specification and differentiation.

Response to Point #5:

We thank the reviewer for suggesting this. We now rephrased this statement. For example, we changed the text:

"... the specification and maintenance of motor neuron identity ... (Revised Line 58)" to "... during motor neuron differentiation ...", and

"... the specification and maintenance of motor neuron identity ... (Revised Line 117)" to "... ES cell differentiation into mature motor neurons ..."

6- Sequencing Data: For ChIP-seq and ATAC-seq experiments, technical replicates were performed, which strengthens the reliability of the data. This should be more clearly stated in the Methods section. Additionally, a comparison between replicates would be informative (e.g., the number of overlapping regions). Using only the common regions between replicates could have helped eliminate potential non-specific peaks, rather than analyzing each replicate separately.

Response to Point #6:
We thank the reviewer for this comment. We have now included the suggested reproducibility analyses for our ChIP-seq and ATATA-seq replicates and have updated the Results and Methods section accordingly. For day 6 ATAC-seq analysis, we used two independent biological replicates, derived from separate motor neuron differentiations, to identify accessible DNA regions in day 6 motor neurons. We identified 17,080 and 23,165 ATAC-seq peaks from replicates 1 and 2, respectively. Among them, the majority of the peaks in one replicate (14,373 out of 17,080 in replicate 1, and 14,373 out of 23,165 in replicate 2) overlapped with the peaks in another replicate (left in Supplementary Fig. 8b).

For the day 6 CREST ChIP-seq analysis, we also used two independent biological replicates to identify CREST-bound regions. We identified 9719 and 8620 CREST-bound regions from replicates 1 and 2, respectively. Among them, the majority of the peaks in one replicate (6357 out of 9719 in replicate 1, and 6357 out of 8620 in replicate 2) overlapped with the peaks in another replicate (right in Supplementary Fig. 8b). Combined with the tag mapping heatmap plots from two independent biological replicates (Supplementary Fig. 8a), these results demonstrate that the ATAC-seq and the CREST ChIP-seq datasets are highly reproducible and reliable for further analysis.

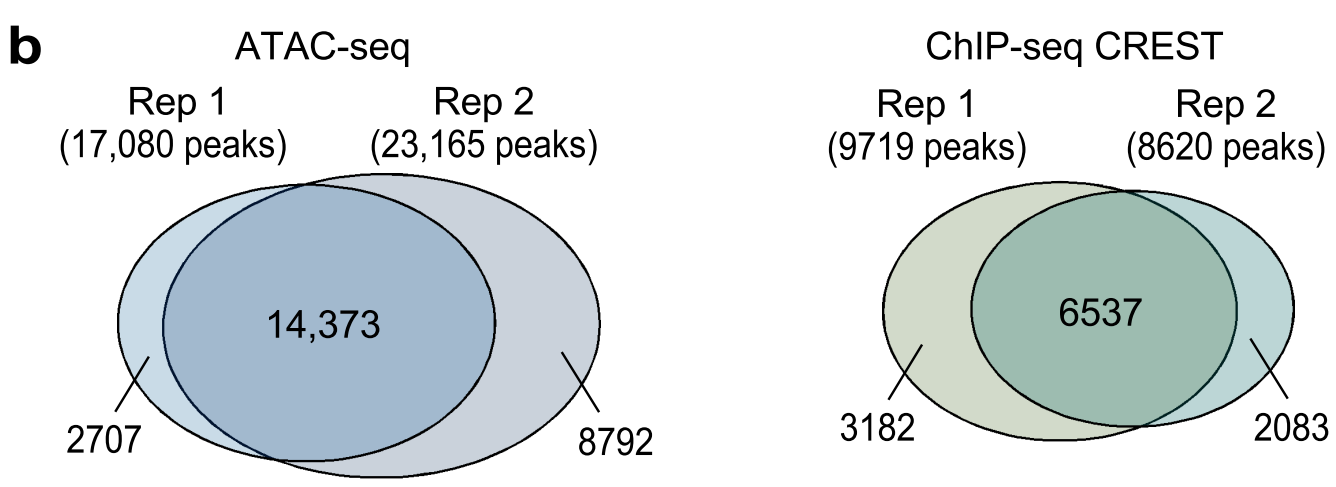

Supplementary Fig. 8b | Day 6 ATAC-seq and ChIP-seq for CREST results in **Fig. 3** are reproducible with independent motor neuron differentiations. b, Venn diagram showing the overlap between two biological replicates of day 6 ATAC-seq and day 6 CREST ChIP-seq peak calls. These overlapped peaks were used for the subsequent analyses.

7- CHIP-seq Data: It is unclear whether a non-ChIP sample (input) was used as a control for the MACS2 analysis.

Response to Point #7:
We thank the reviewer for this comment. We used non-ChIP samples (input) as a control for the MACS2 analysis. We have now updated the Methods section to clarify that ChIP-seq controls (with IgG antibody or no antibody) were used as the negative control for the MACS2 peak-calling analysis.

8- ATAC-seq: Genomic regions were classified as promoters if they were located within 3 kb of the TSS. For greater precision, it would be beneficial to compare the identified peak regions with annotated known promoter regions.

Response to Point #8:
We thank the reviewer for this comment. To address the reviewer's suggestion, we evaluated the accessible promoter regions by comparing them with 15-state chromHMM annotations (chromatin-state signatures using a multivariate hidden Markov model) from mouse E11.5 hindbrain tissue

(ENCODE dataset; ENCSR998GUD) - a developmental stage and brain region where motor neuron production and neurogenesis are actively occurring (Ernst and Kellis, Nature Protocol, 2017; PMID: 29120462). This allows an orthogonal validation of our ATAC-seq-defined promoters, as the chromHMM annotations are based on histone modifications characteristic of promoter activity. Aligning the accessible promoters to these annotations revealed that ~93.4% overlapped with either active or bivalent TSSs (Supplementary Fig. 6e), suggesting that they are likely functioning as an active promoter in this cellular context.

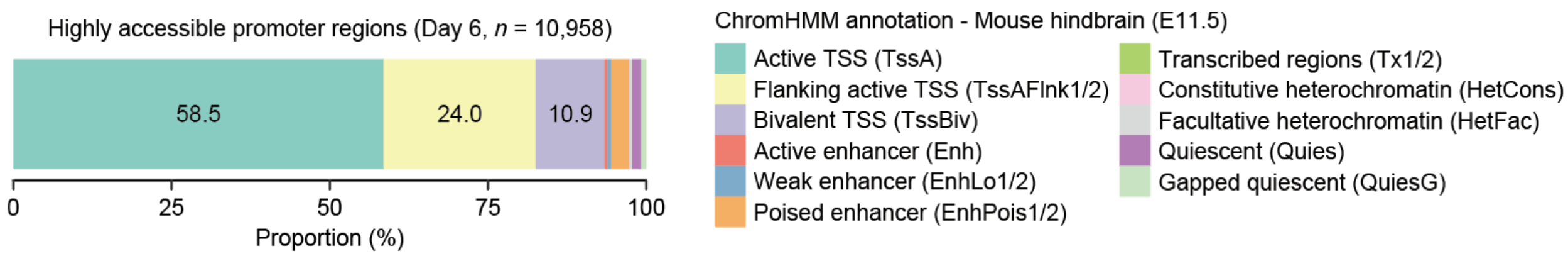

Supplementary Fig. 6e | Stacked bar plot showing the proportion of accessible promoter regions at day 6 with respect to 15-state chromHMM annotations derived from the E11.5 mouse hindbrain.

9- Line 207: It was indicated that 29.4% (4221 out of 14,373) of highly accessible distal cis-regulatory regions are occupied by CREST, meaning that 73% (4221 out of 5726) of CREST-bound regions are highly accessible. The analysis of the 27% of regions that are bound but not accessible could provide additional insights. It would be useful to explore whether these regions are associated with genes that are not expressed.

Response to Point #9a:

→ We thank the reviewer for this constructive input. Of the 6215 CREST-bound distal regulatory regions in the revised manuscript, 1994 regions were detected as inaccessible (or weakly accessible), while the remaining 4221 regions were highly accessible. Notably, the inaccessible regions were weakly bound by CREST, whereas the highly accessible regions showed strong CREST binding (Supplementary Fig. 6b). Additionally, GO analysis revealed that genes associated with the in accessible CREST-bound regions were enriched for non-neuronal genes and consistently expressed during motor neuron differentiation (Supplementary Fig. 6c, d). In contrast, GO analysis showed that genes associated with the highly accessible CREST-bound regions were enriched for neuronal genes and specifically expressed in day 6 motor neurons (**Fig. 3c**). These results suggest that the inaccessible regions weakly bound by CREST are not associated with neuron-specific functions or differentiation. Given the observed antibody cross-reactivity between CREST (SS18l1) and its paralog protein SS18 (**Fig. 1d**, Supplementary Fig. 2c), we suspect that these weak ChIP-seq signals might represent background arising from Ss18 cross-reactivity.

b

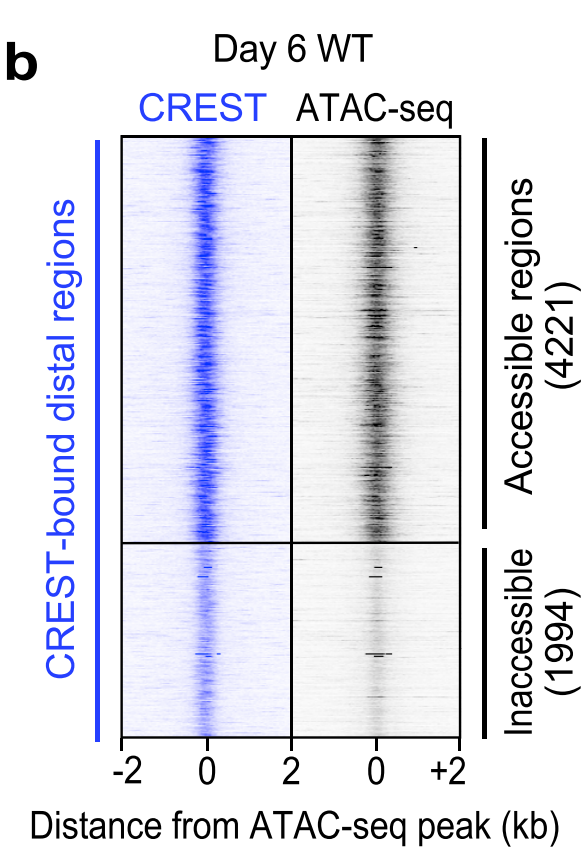

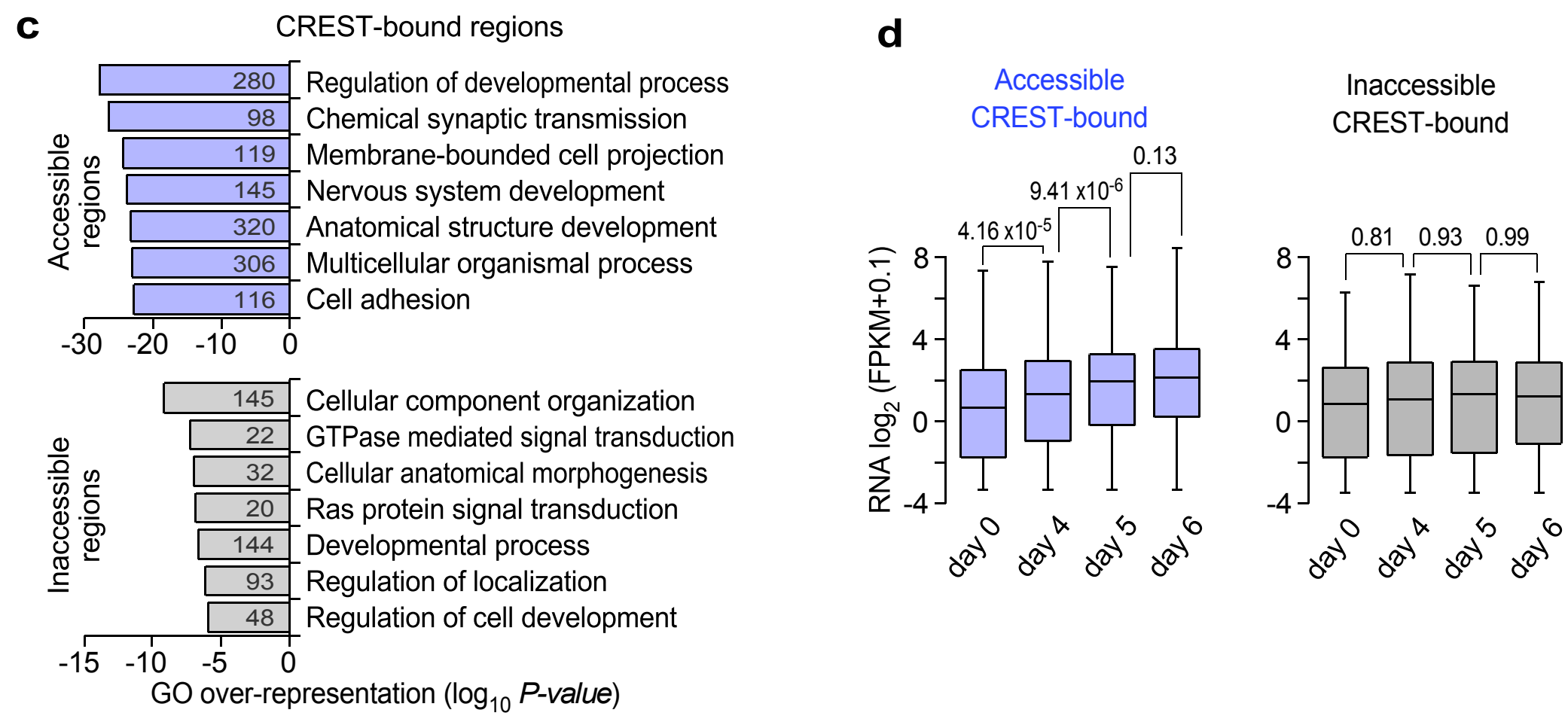

Supplementary Fig. 6 | **b,** Genomic mapping of ChIP-seq CREST and ATAC-seq in day 6 WT motor neurons. 6215 CREST-bound distal cis-regulatory regions, defined in Fig. 3a, were grouped by accessible regions (n = 4221), overlapped with ATAC-seq peaks, and inaccessible regions (n = 1994) in day 6 WT motor neurons. All data were plotted relative to the ATAC-seq peak midpoints. **c,** GO analysis of the 1082 genes associated with CREST-bound accessible distal regions (same as shown in Fig. 3c) and the 392 genes associated with CREST-bound inaccessible distal regions shown in panel (b). The seven most over-represented and non-redundant GO terms are displayed. Numbers within bars represent the count of genes associated with each GO term. **d,** Box plots showing RNA expression (log2 FPKM+0.1) for 1082 genes linked to accessible CREST-bound regions and 386 genes linked to inaccessible CREST-bound regions, identified in panel (b). FPKM values represent the average of three biological replicates (detailed in Fig. 2a). Boxes display the first and third quartiles with the median indicated by the center line; whiskers extend to 1.5x the interquartile range. P-values shown above the whiskers were determined using a one-way analysis of variance (ANOVA) followed by Tukey's post hoc tests. Specific ANOVA results are as follows: Accessible group, F(3,8) = 52.6, P = 2.28 x 10^{-33}; and inaccessible group, F (3,8) = 1.29, P = 0.78. Complete statistical values are provided in Supplementary Data 8.

10- Line 240: Using ChIP-seq, the authors show that CBP binding is decreased in CREST-KO cells. Since this decrease appears to be genome-wide, it would be advisable to check CBP expression levels to ensure that the absence of CREST does not affect CBP expression.

Response to Point #10a:

We thank the reviewer for this comment. To address this, we assessed CBP mRNA levels of CBP and observed an approximately 31.5% reduction following *CREST* knockout (Supplementary Fig. 7c). These results suggest that the absence of CREST may partially downregulate CBP expression and diminish its genomic binding at specific enhancers.

c

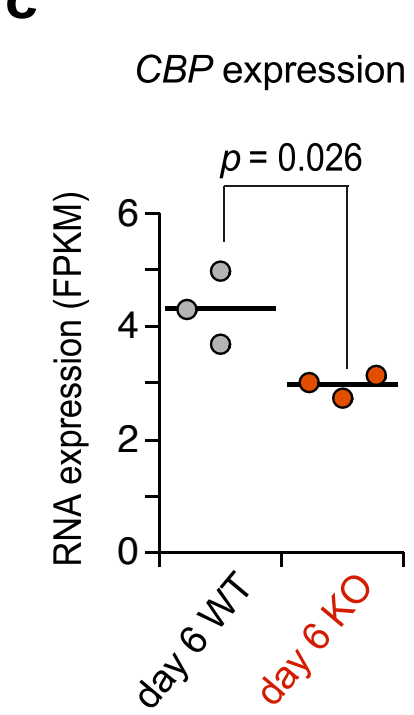

Supplementary Fig. 7c | RNA expression (FPKM) of the CBP gene was measured by RNA-seq in day 6 WT mature motor neurons and day 6 KO cells. FPKM values represent averages from three biological replicates on day 6 in WT and KO conditions (Supplementary Data 3). Lines in the dot plots show the average. P-values were calculated by a two-sided, two-sample Student's t-test.

9- Line 268: The Hi-C method, followed by ABC analysis, enabled the authors to assign distal regions to their cognate promoters, thereby directly identifying the target genes of CREST. Including the number of target genes would further strengthen the findings. Additionally, earlier in the study, a list of bound genes was established by associating peaks with the closest gene. A comparison between these two lists would provide a clearer perspective on the depth of the analysis.

Response to Point #9b:
We appreciate this valuable comment. The number of target genes of CREST-bound regions based on activity-by-contact was 5,320. A direct comparison between these distal target genes and the closest genes showed that the majority of the closest genes (826 of 1,082; 76.3%) were included among the distal targets (Supplementary Fig. 10a). This highlights that the activity-by-contact model, which integrates Hi-C and ATAC-seq data, effectively reveals the genome-wide distal regulatory roles of CREST-bound accessible sites.

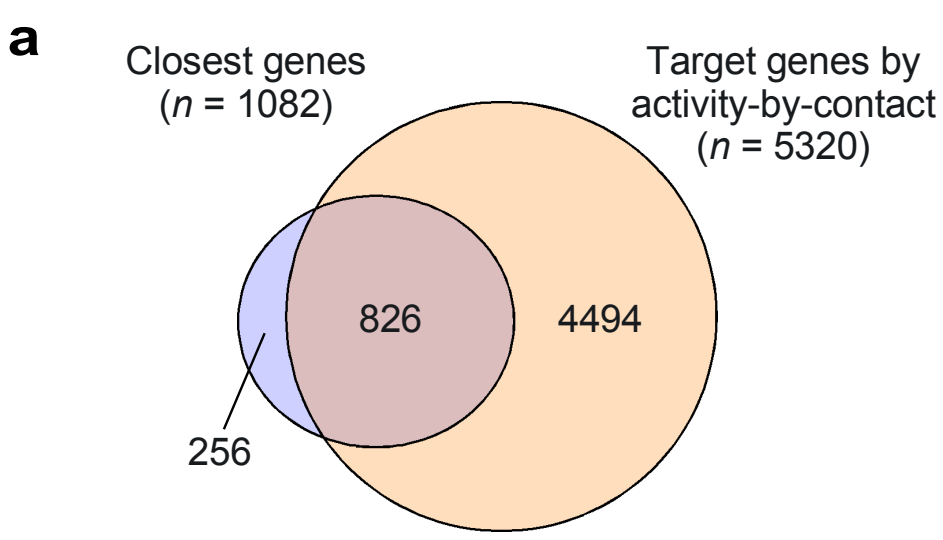

Supplementary Fig. 10a | Venn diagram showing the overlap between the closest proximity genes, defined in Fig. 3c (n = 1082; Supplementary Data 4), and the distal target genes (n = 5320) of CREST-bound accessible regions, as identified by Hi-C Activity-by-Contact (ABC) analysis.

10- Line 271: A comparison between CREST-bound genes and DEGs is presented, but no numbers are provided. Including the numbers and proportions of direct CREST targets would further strengthen the analysis.

Response to Point #10b:
We thank the reviewer for this helpful comment. A direct comparison of the gene sets showed that, of the 3,043 DEGs, 362 (11.9%) were the closest genes to CREST-bound regions, whereas 1,098 (36.1%) were identified as target genes of CREST-bound regions based on activity-by-contacts (Supplementary Figure 10b). This indicates that Hi-C-based analysis of distal gene regulation enables more robust mapping of CREST-bound regions and their targets, in comparison with proximity-based mapping. Additionally, we found that 61.3% (1065 out of 3043) were not identified by either the ABC model or proximity or proximity assignment, suggesting that their gene expression changes are indirectly associated with CREST genomic binding.

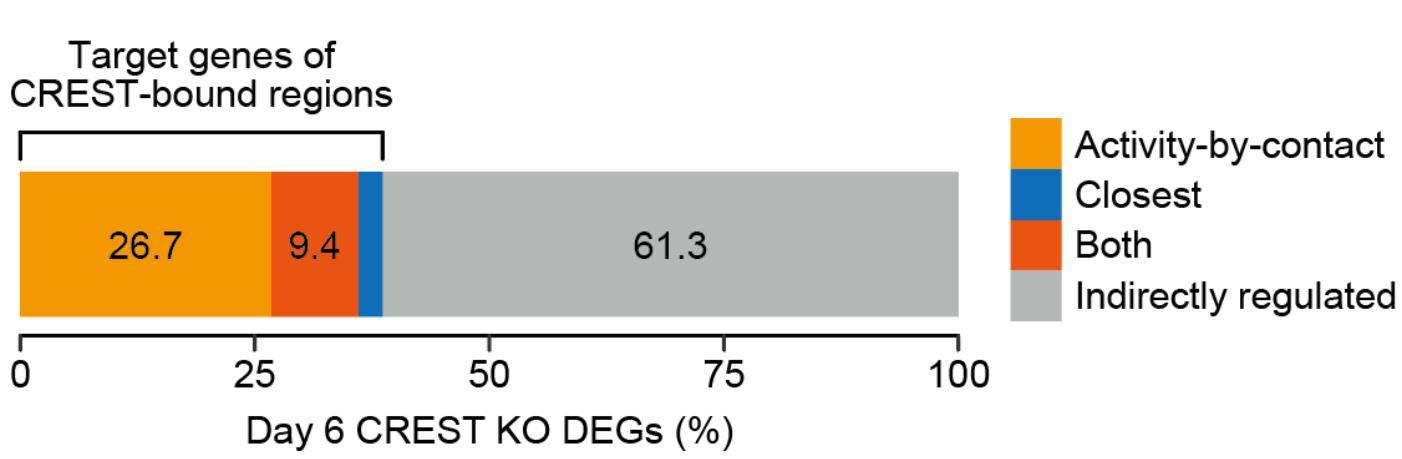

Supplementary Fig. 10b | Stacked bar plot showing the proportion of day 6 CREST knockout differentially expressed genes (DEGs) associated with CREST-bound accessible regions. These target genes are identified either by proximal localization (the closest genes) or Hi-C ABC analysis. The “Both” category (9.4%) indicates target genes identified by both methods.

11- Hi-C: ChIP-seq data show that CREST primarily binds to distal regions (enhancers). It would be useful to examine whether the few bound promoters correlate with their cognate enhancers. Specifically, it would be informative to explore whether the presence of CREST at promoters reflects a loop between enhancer and promoter. Additionally, investigating whether genes with CREST binding at promoters are particularly accessible or expressed could provide further insights.

Response to Point #11:
We thank the reviewer for this valuable feedback. Consistent with the low incidence of CREST binding at promoters (4.92%; **Fig. 3a**), the proportion of chromatin loops (promoter–enhancer interactions with an ABC score > 0.1) involving CREST-bound promoters was also small (2.71%; Response Fig. 1A). When we examined how CREST-bound promoters interact with CREST-bound distal elements, we found that the proportion of CREST-bound promoters interacting with CREST-bound distal accessible regions was higher than that of CREST-unbound promoters (Response Fig. 1B). This suggests that, although only a small fraction of active promoters show CREST occupancy, those that do may form more frequent chromatin contacts with CREST-bound distal enhancers. In line with this observation, CREST-bound promoters displayed somewhat elevated expression, although the difference compared to CREST-unbound promoters did not reach statistical significance, due to the small number of CREST-bound promoters. Overall, while CREST-bound promoters may contribute to gene regulation through interactions with CREST-bound distal enhancers, their overall fraction is very small, and the vast majority of transcriptional regulation driving motor neuron–specific gene expression is likely mediated by CREST binding at distal regulatory regions. Because these results do not alter our primary conclusions, we think that including these analyses may potentially complicate our main messages within the word limit of the manuscript (5000 words). While we greatly appreciate the reviewer's valuable feedback, we have opted not to incorporate this analysis into the revised manuscript.

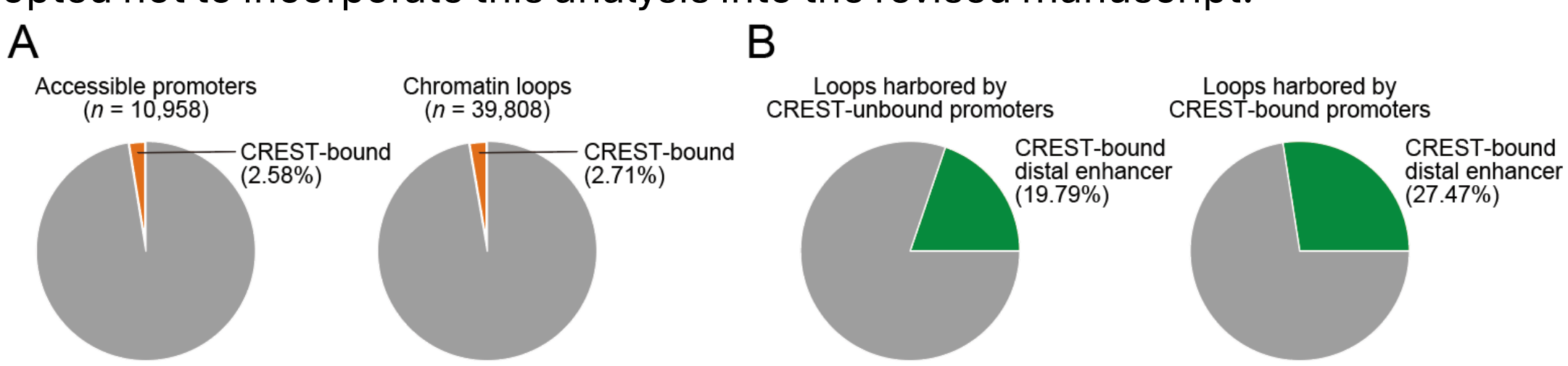

Response Fig. 1 | Analysis of CREST-bound promoters. A. Pie charts showing the fraction of CREST-bound promoters in accessible promoters (A) and chromatin loops anchored by CREST-bound promoters (B). B. Pie charts showing the fraction of chromatin loops involving CREST-bound distal enhancers in CREST-unbound promoters (A) and CREST-bound promoters (B).

12- Line 491: H3K4me1-ChIP data are mentioned but not presented. Including the H3K4me1 data would provide valuable additional context.

Response to Point #12:
We thank the reviewer for suggesting this experiment. We included ChIP-seq H3K4me1 data (**Fig. 5a,d,e**, Supplementary Fig. 14) to support our statement that the primed enhancer mark H3K4me1 is present at CREST-bound enhancers as well as CREST-unbound enhancers. This primed enhancer mark may be involved in the initial recruitment of BAF complexes to enhancers.

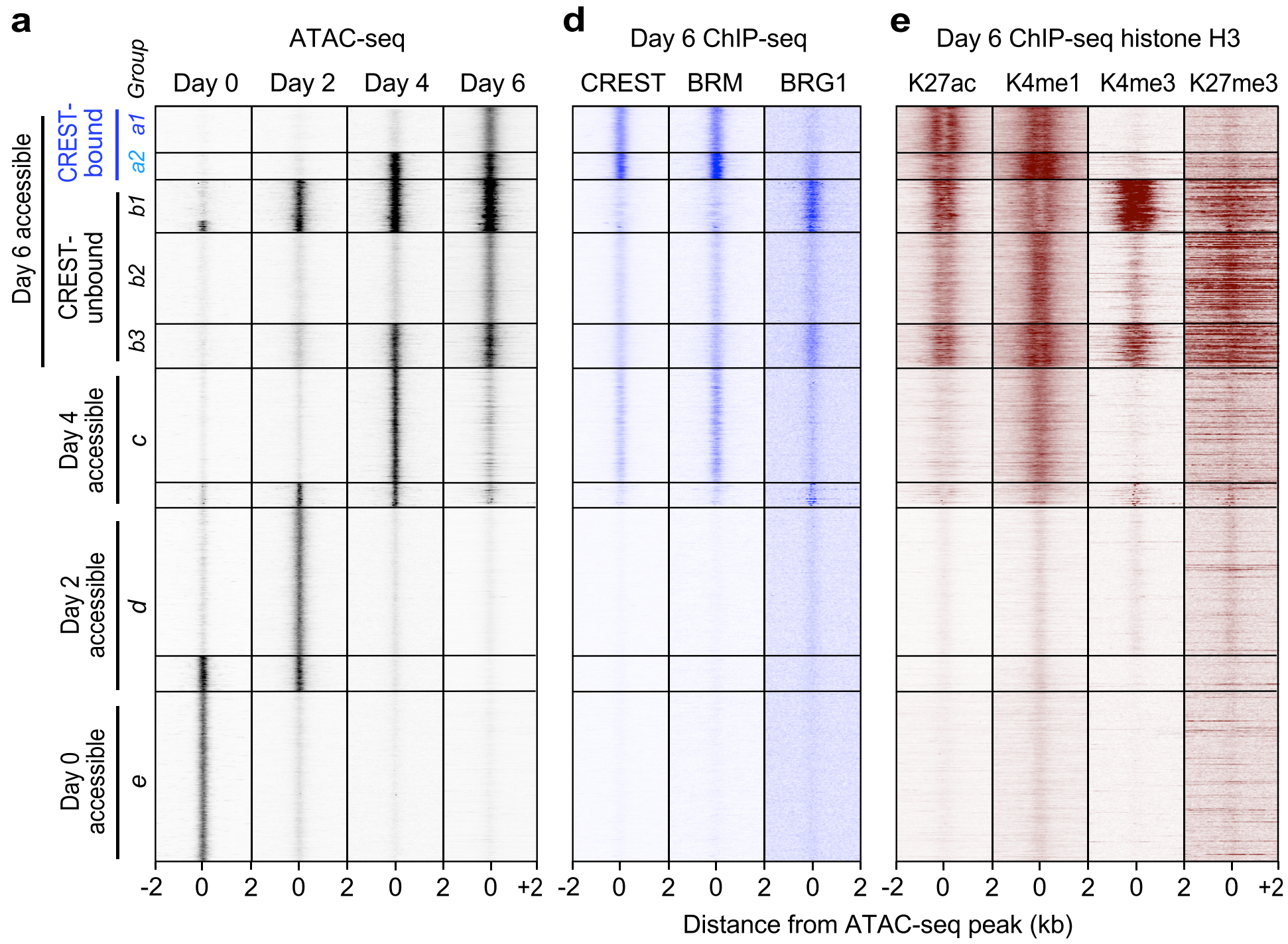

Figure 5 | **a,** ATAC-seq mapping of 38,091 accessible distal cis-regulatory regions identified in day 0, 2, 4, and 6 differentiated cells. ATAC-seq peaks were grouped by CREST-bound day 6 accessible regions (group a1), CREST-bound day 4 and day 6 accessible regions (group a2), and CREST-unbound regions mainly accessible from day 2 to day 6 (group b1), accessible specifically on day 6, day 4, day 2, and day 0 cells (groups b2, c, d, and e, respectively). All data were plotted relative to the ATAC-seq peak midpoints, reported in Supplementary Data 7. **d-e,** ChIP-seq mapping of CREST, BRM, BRG1, H3K27ac, H3K4me1, H3K4me3, and H3K27me3 in day 6 motor neurons at the accessible distal cis-regulatory regions identified in panel (a). All data were plotted relative to 38,091 ATAC-seq peak midpoints in the same order as shown in panel (a).

Reviewer #3 (Remarks to the Author):

I co-reviewed this manuscript with one of the reviewers who provided the listed reports. This is part of the Nature Communications initiative to facilitate training in peer review and to provide appropriate recognition for Early Career Researchers who co-review manuscripts.

Reviewer #4 (Remarks to the Author):

Summary
Montanera et. al., investigate the role of the neuron-specific BAF complex subunit CREST in motor neuron (MN) differentiation using a mESC-derived MN model and a suite of chromatin profiling tools including RNA-seq, ChIP-seq, ATAC-seq, and Hi-C. Their findings show that CREST does not mediate long-range chromatin interactions but instead facilitates CBP-mediated enhancer activation through acetylation at H3K27. These CREST-bound enhancers are enriched for MN transcription factor motifs, specifically Onecut1 and Ngn2, and show selective co-occupancy with Brm but not Brg1. This suggests a possible mechanism for cell type-specific BAF subunit recruitment.
Overall, the manuscript is conceptually compelling and provides valuable insight into how neuron-specific chromatin regulators establish subtype-specific gene programs. The data supports a model in which transcriptional and chromatin remodeling machinery recruits CREST-CBP to motor neuron enhancers for deposition of activating histone modifications. Addressing key experimental gaps, particularly around model validation, knockout specificity, and the disease relevance of a CREST KO model, would help solidify the conclusions and broaden the impact of the study.

Major Comments

1. Validation and characterization of cell models
a. The authors should provide more robust evidence that their day 6 ES-derived cultures contain fully mature MNs. While the chosen RNA-seq markers and MAP2 staining are supportive, Map2 is not specific to motor neurons. Consider including additional markers such as ChAT, SMI32, or even electrophysiological profiles, or provide further rational for the current choices.

Response to Point #1a:
Thank the reviewer for suggesting this analysis. To examine the efficiency of differentiation of our ES cells into day 6 mature motor neurons, we evaluated the expression of the cholinergic motor neuron marker ChAT throughout the differentiation process. We confirmed that the protein level of ChAT was not detectable on days 2, 4, or 5, but they were specifically expressed in day 6 cells (Supplementary Fig. 1a). Additionally, we observed that other mature motor neuron markers (VAChT, ChT1, and Smim32) were exclusively expressed in day 6 wildtype motor neurons (Supplementary Fig. 1c).

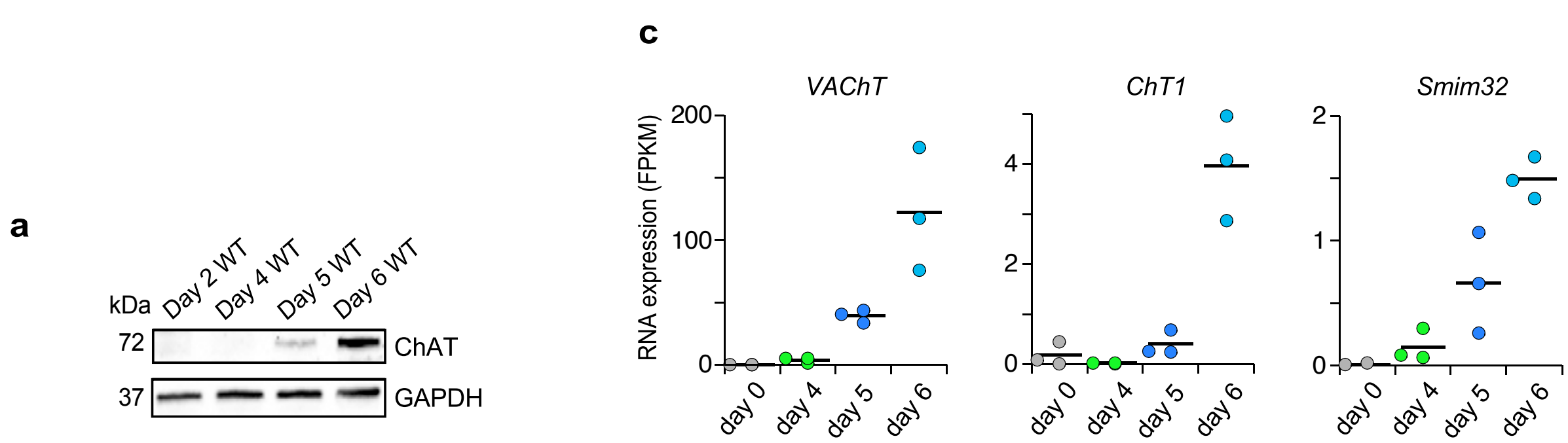

Supplementary Fig. 1 | **a,** Western blot confirmed that the cholinergic neuronal marker ChAT is expressed at ~72 kDa (kilodaltons) only in day 6 wild-type (WT) motor neurons during the differentiation. A housekeeping protein GAPDH (~37 kDa) was used as a loading control. **c,** RNA expression (FPKM) of three genes encoding motor neuron markers, VAChT, ChT1, and Smim32, measured by RNA-seq during motor neuron differentiation (day 0 ES cells, day 4 neural progenitors, day 5 nascent motor neurons, and day 6 WT mature motor neurons) from three biological replicates (n = 3) of independent cell differentiations. Lines in the dot plots show the average.

b. The manuscript does not address the purity of MN cultures, which is critical given the potential for signal dilution or contamination in bulk omics assays. Were any FACS-based purifications or single-cell validations performed?

Response to Point #1b:
We thank the reviewer for this comment. To examine the purity of our motor neuron culture, we quantified the percentage of cells positive for the motor neuron markers Isl1 and Hb9 on day 6. Our analysis revealed that 78.7% and 56.5% of all transcriptionally active day 6 cells were positive with Isl1 and Hb9, respectively (Supplementary Fig. 1d). Together, these results demonstrate that our differentiation system yields fully mature motor neurons at a high purity and that CREST is specifically expressed in these day 6 mature motor neurons.

Additionally, to computationally infer the purity of motor neurons in day 6 wild-type mRNA-seq data, we examined the cellular heterogeneity of the motor neuron differentiation culture using CIBERSORTx (Nature Biotechnol., 2019, PMID: 31061481). We obtained scRNA-seq data generated from adult mouse cord tissue (Signal Transduction and Targeted Therapy, 2022, PMID: 35232960) and used it as a reference to estimate the cellular proportions across 12 cell types present in adult mouse spinal cord. The motor neuron purity for wild-type mRNA-seq samples ranged from 71% to 83% (replicate 1: 71.5%, replicate 2: 73.6%, and replicate 3: 83.6%) (Response Fig. 2). Together, these results suggest that the majority of day 6 differentiated cells from our motor neuron culture system are motor neurons with substantial purity. Because these results do not alter our primary conclusions, we think that including these CIBERSORTx analyses may potentially complicate our main messages within the word limit of the manuscript (5000 words). While we greatly appreciate the reviewer's valuable feedback, we have opted not to incorporate this analysis into the revised manuscript.

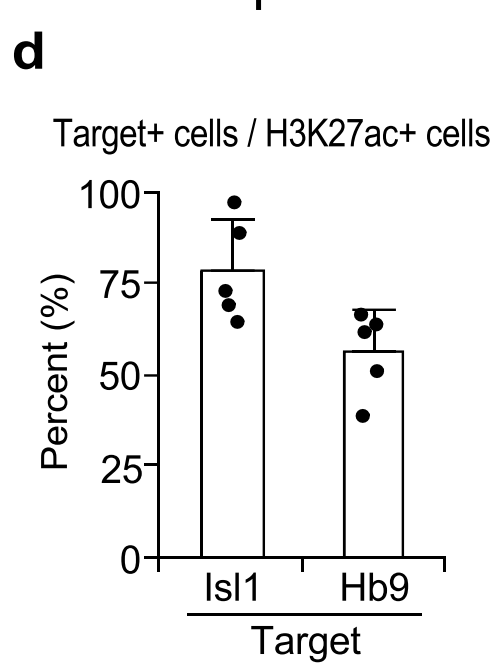

Supplementary Fig. 1d | Quantification of transcriptionally active (H3K27ac-positive) day 6 WT cells expressing the motor neuron-specific markers ISL1 or HB9. Embryoid bodies were immunostained for HB9, ISL1, and H3K27ac, followed by the quantification of immuno-positive cells. Data are presented as mean ± SEM (n = 5 embryoid bodies per target marker).

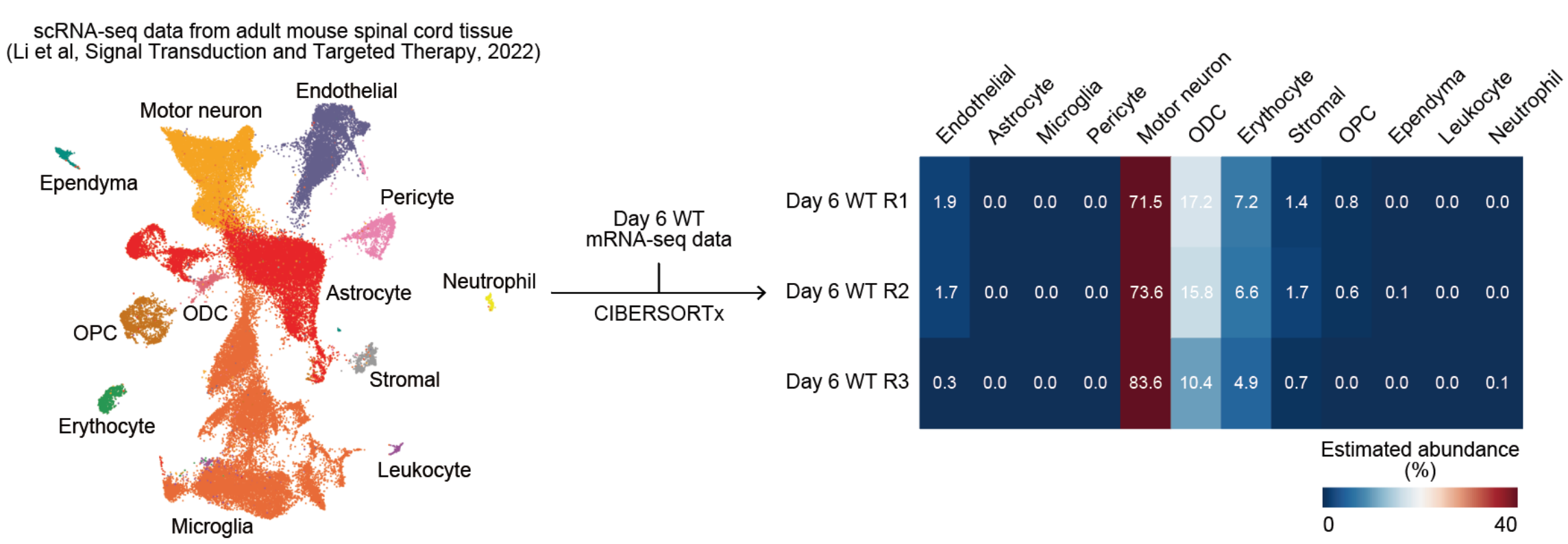

Response Fig. 2 | Estimation of motor neuron purity in day 6 WT mRNA-seq data via CIBERSORTx, using scRNA-seq data from adult mouse spinal cord tissue as the reference.

c. The IHC in Figure 1 shows persistent CREST signal in the KO line. This contradicts the western blot and raises concerns about knockout efficiency. Please clarify whether this is due to mosaic KO, antibody background, or something else.

Response to Point #1c:

We thank the reviewer for suggesting this clarification. To address this concern, we performed multiple western blotting experiments with antibodies against CREST (SS18L1) and its paralog SS18, which share high sequence homology (63 - 69% amino acid identity depending on their isoforms). We tested two cell conditions: (i) day 2 WT and day 6 KO cells (expressing only SS18), and (ii) day 6 WT motor neurons (expressing both CREST and SS18). We detected the western blotting signal by the CREST antibody in the day 6 KO cells (**Fig. 1d**, Supplementary Fig. 2c). Since this residual signal was also observed in day 2 WT cells (which do not express CREST; **Fig. 1c**), we conclude that the remaining CREST signal in day 6 KO cells is due to antibody cross-reactivity with the paralog SS18, likely recognizing a conserved epitope. The CREST antibody used (ProteinTech, Cat# 12439-1-AP) was raised against a 350-amino acid immunogen from the recombinant human CREST protein. Sequence alignment using the NCBI Protein BLAST reveals that 208 of these 350 amino acids are identical to the mouse SS18 protein.

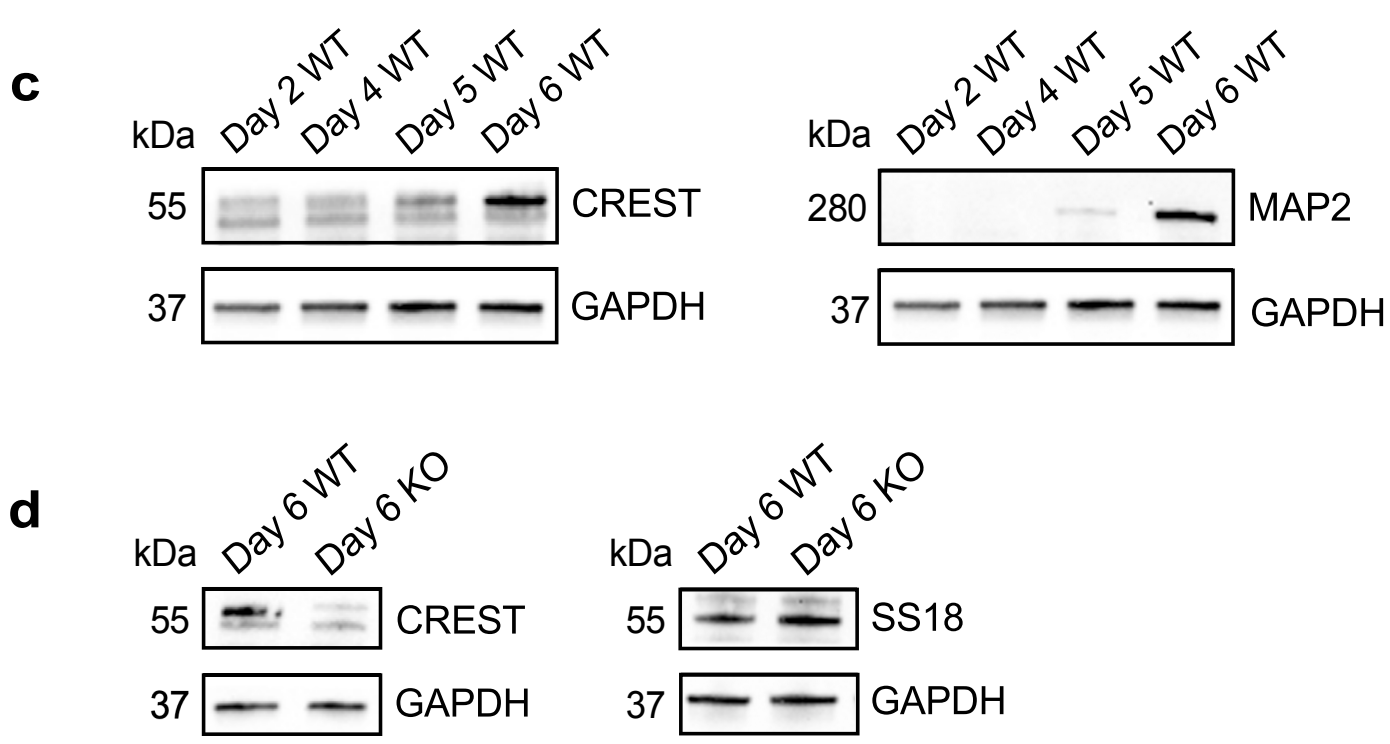

Figure 1 | **c,** Western blot analysis confirms that CREST (SS18L1) and the mature neuronal marker MAP2 are detected at ~55 kDa (kilodaltons) and ~280 kDa, respectively, only in day 6 WT motor neurons during differentiation. The housekeeping protein GAPDH (~37 kDa) serves as a loading control. **d,** Western blot analysis confirms that the CREST knockout (KO), generated via CRISPR-Cas9, eliminates CREST protein expression in day 6 differentiated cells. CREST is detected at ~55 kDa (upper band) in day 6 WT cells but is absent in day 6 KO cells. The CREST paralog, SS18, is detected at ~55 kDa (lower band) in both day 6 WT and day 6 KO cells. Biological replicates are shown in Supplementary Fig. 2c.

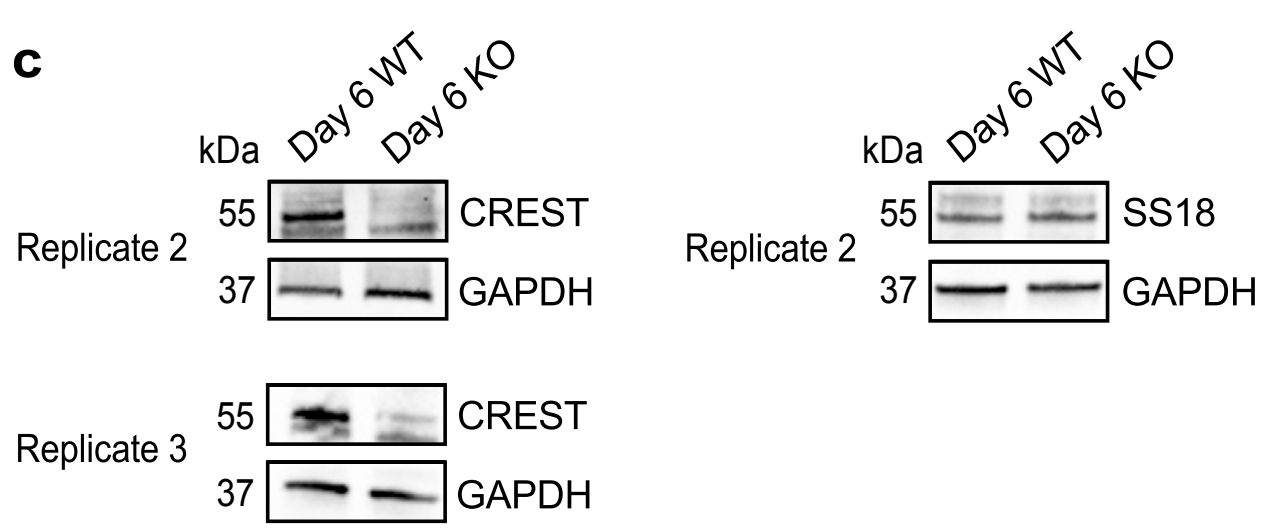

Supplementary Fig. 2c | Western blot confirmed that the CREST knockout, generated via CRISPR/Cas9, causes a knockout in CREST protein level in day 6 differentiated cells. CREST is expressed at ~55 kDa (upper band) in day 6 WT cells, but not in day 6 KO cells. The CREST paralog, SS18, is expressed at ~55 kDa (lower band) in both day 6 WT and KO cells. The second and third biological replicates are shown here. The first replicates were shown in Fig. 1d.

d. The claim that CREST KO results in reduced Hb9 and Isl1 expression (lines 115-117) should be supported by quantification, as the images are not conclusive.

Response to Point #1d:
We thank the reviewer for this comment. Please see our previous response to Reviewer 2's comment - Point #4.

e. The analysis stops at day 6. Later-stage timepoints would clarify whether CREST KO fully prevents MN differentiation preserving a naive state, or just delays MN differentiation.

Response to Point #1e:
We thank the reviewer for suggesting this experiment. To test whether CREST knockout prevents motor neuron differentiation or delays it to a later developmental stage than day 6 motor neurons, we extended the differentiation period by 3 days (termed day 9 motor neurons), followed by RNA-seq on day 9 WT and day 9 KO cells. To identify genes associated with neuronal maturation in day 9 wildtype cells compared to day 5 nascent motor neurons, we used the same mRNA gene classification described in **Fig. 2a.** For this, we divided 1614 genes downregulated in day 6 wildtype compared to day 6 KO (Group 1 in **Fig. 2a**) into two sub-groups: *1a)* 879 genes downregulated or unchanged on day 9 compared to day 5, and *1b)* 735 upregulated on day 9 compared to day 5 (Supplementary Fig. 16a). GO analysis showed that Group *1b* genes (upregulated on day 9) were significantly enriched with mature neuronal functions, including chemical synaptic genes (Supplementary Fig. 16b). The expression levels of these mature neuronal genes (Group *1b*) were significantly downregulated in day 6 KO cells compared to day 6 wildtype motor neurons, but were fully recovered in day 9 KO cells compared to day 6 wildtype and day 9 wildtype cells (Supplementary Fig. 16c). Moreover, the overall expression levels of these mature neuronal genes were similar between day 6 wildtype, day 9 wildtype, day 9 KO cells. These results suggest that CRST knockout delays motor neuron differentiation rather than fully preventing it.

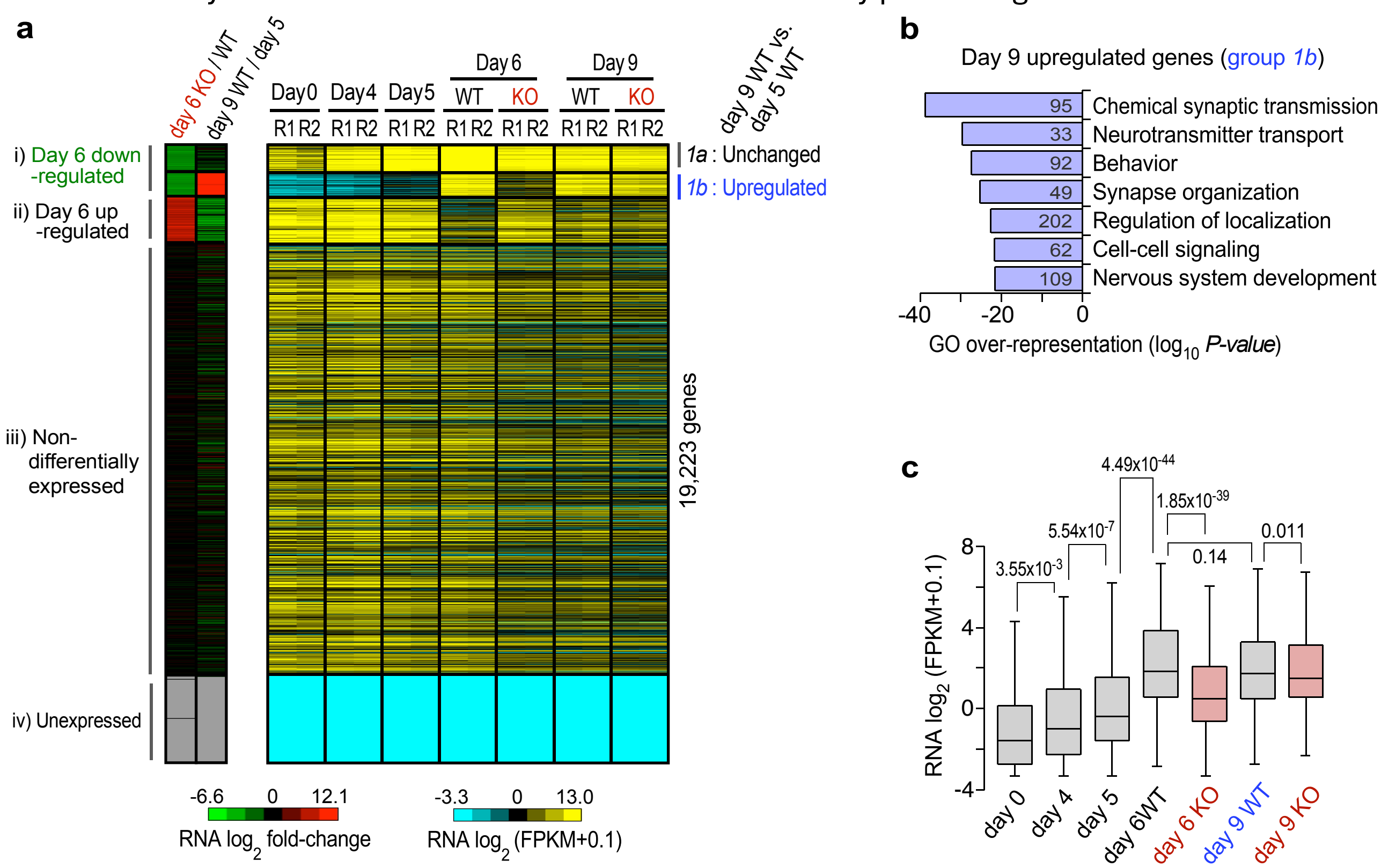

Supplementary Fig. 16 | CREST knockout delays the neuronal maturation, extending the differentiation process from 6 days to 9 days. **a,** Left: Heatmap of RNA expression (log2 fold-changes) of all 19,223 mRNA genes in day 6 KO cells compared with day 6 WT cells. Genes are ordered as displayed in Fig. 2a, except for the Day 6 downregulated genes (Group i), which were further subdivided based on RNA expression changes in day 9 WT cells compared with day 5 WT cells (Group 1a: 879 unchanged or downregulated genes; Group 1b: 735 upregulated genes). Right: Heatmap of RNA expression (log2 FPKM+0.1) of all mRNA genes in days 0, 4, 5, and 6 WT and day 6 KO cells, as well as day 9 WT and day 9 KO cells. FPKM values were adjusted by adding 0.1, median-normalized, log2-transformed, and ordered as displayed on the left. The heatmap includes data from two biological replicates (R1 and R2) derived from independent cell differentiations. **b,** GO analysis of 735 upregulated genes (Group 1b) identified in panel (a). The seven most over-represented and non-redundant GO terms are displayed. Numbers within bars represent the count of genes associated with each GO term. **c,** Box plots showing RNA expression (log2 FPKM+0.1) for upregulated 95 chemical synaptic transmission genes (Group 1b, the top GO term genes), identified in panel (b). FPKM values represent averages from two biological replicates, shown in panel (a). The plots display the median (line within boxes), first-to-third quartiles (bottom and top of boxes, respectively), and 1.5x the interquartile range (whiskers). A two-sided paired t-test.

2. Relevance to ALS and CREST mutations

a. The current study employs a full knockout, yet ALS-associated CREST mutations are heterozygous with one allele fully intact and the other containing a Q388X truncation of the C-terminal region required for CBP interaction. Comparing a CREST +/- or Q388X mutant line as in Cheng et al, 2019, would greatly enhance disease relevance.

Response to Point #2a:

We are thankful for this constructive feedback. Although we primarily used homozygous mutant cells (*CREST* $^{-/-}$; also referred to as day 6 *CREST* ^{KO} cells) for our analyses, we also investigated whether day 6 homozygous mutant cells (*CREST* $^{+/-}$), highly relevant to ALS-associated mutations, exhibit similar phenotypes to day 6 *CREST* $^{-/-}$ cells. To address this, we generated heterozygous ES cells (*CREST* $^{+/-}$; Supplementary Fig. 4a) and differentiated them into day 6 motor neurons. As observed in day 6 *CREST* $^{-/-}$ cells (**Fig. 1d**), the expression levels of the motor neuron markers Isl1 and Hb9 were decreased in day 6 *CREST* $^{+/-}$ cells compared to day 6 WT motor neurons. RNA-seq analysis further revealed that global gene expression patterns in day 6 *CREST* $^{+/-}$ cells were highly similar to those in day 6 *CREST* $^{-/-}$ cells (Supplementary Fig. 4b). Specifically, genes enriched for neuronal processes, which were downregulated in the day 6 homozygous mutant cells compared to day 6 WT cells (group *i*; **Fig. 2a**), were also significantly downregulated in day 6 *CREST* $^{+/-}$ cells (Supplementary Fig. 4c). Specifically, mature neuronal genes (Group i) were downregulated, and cell cycle-related genes (Group ii) were upregulated in both lines (Supplementary Fig. 4d), demonstrating that heterozygous *CREST* mutations drive similar, albeit milder, transcriptomic changes. These results demonstrate that while the homozygous *CREST* mutation exhibits more severe gene expression changes, the heterozygous *CREST* mutation drives a similar, albeit milder, alteration in the expression of key genes regulated during motor neuron differentiation. Therefore, we utilized a homozygous *CREST* mutation to elucidate the mechanistic role of CREST in our subsequent analyses.

As shown previously (Reviewer 1, Point #2), the study by Chen et al. (2019, Translational Neurodegeneration, PMID: 30976389) examined the effects of CREST mutations in mice. However, the microarray dataset was not made publicly available, and we were unable to obtain the data despite our efforts to contact the corresponding author. Therefore, as an alternative, we analyzed an mRNA-seq dataset from Catanese et al. (2021, EMBO Molecular Medicine, PMID: 34125498) derived from human iPSC-derived motor neurons of ALS patients carrying C9orf72 (two patients) and FUS (two patients) mutations, along with controls including one healthy individual and a CRISPR-corrected line derived from a C9orf72-mutant individual (Supplementary 4e). Gene set enrichment

analysis (GSEA) revealed that CREST KO-associated DEGs at day 6 were significantly correlated with transcriptional alterations observed in both C9orf72- and FUS-mutated motor neurons. These results demonstrate that CREST KO-induced transcriptional changes are highly relevant to human ALS-associated mutations.

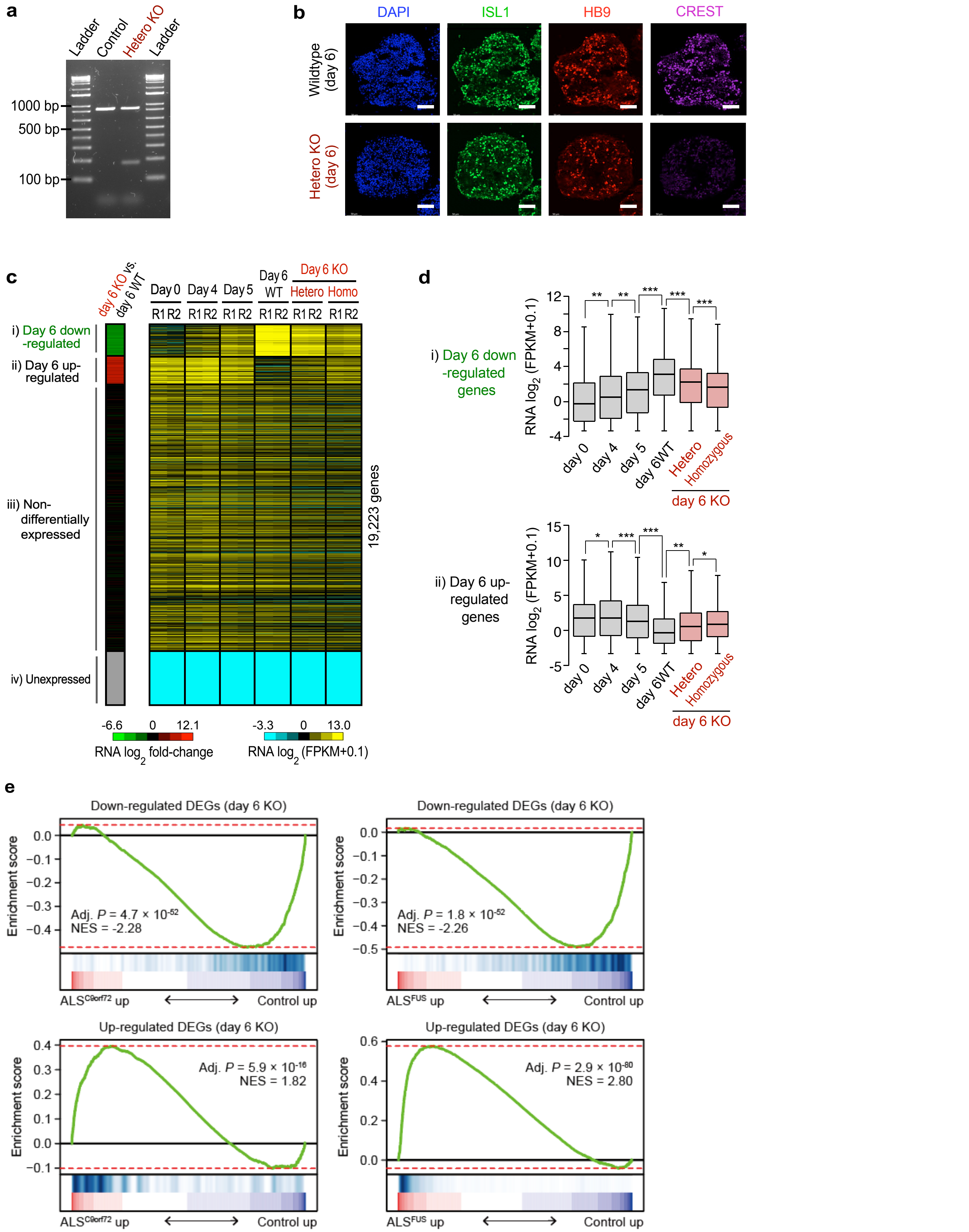

Supplementary Fig. 4 | The heterozygous CREST knockout mutation drives gene expression changes similar to those of the homozygous CREST knockout mutation. **a,** Genotyping of a heterozygous CREST KO ES cell colony (CREST +/-), generated via CRISPR/Cas9, analyzed on a 2% agarose gel. A 710 bp deletion (67 + 643 bp) was confirmed only in one allele, while the other allele remained intact. The control lane represents the genotype of a WT colony. **b,** Immunostaining of differentiated embryoid bodies showing the nuclear-specific dye DAPI, the motor neuron-specific markers ISL1 and HB9, and CREST in day 6 WT motor neurons and day 6 heterozygous KO cells (CREST +/-). Scale bars represent 50 µm. **c,** Left: Heatmap of RNA expression (log2 fold-changes) of all 19,223 mRNA genes, ordered as displayed in Fig. 2a, in day 6 KO cells (CREST -/-) compared with day 6 WT motor neurons. The four gene groups were defined in Fig. 2a. Right: Heatmap of RNA expression (log2 FPKM+0.1) of all mRNA genes in days 0, 4, 5, and 6 WT cells, day 6 heterozygous mutant cells (CREST +/-), and day 6 homozygous mutant cells (CREST -/-). FPKM values were adjusted by adding 0.1, median-normalized, log2-transformed, and ordered as displayed on the left. The heatmap includes data from two biological replicates (R1 and R2) derived from independent cell differentiations. **d,** Box plots showing RNA expression (log2 FPKM+0.1) for 1614 downregulated genes (Group i) and 1429 upregulated genes (Group ii) shown in panel (c). FPKM values represent averages from two biological replicates, shown in panel (c). The plots display the median (line within boxes), first-to-third quartiles (bottom and top of boxes, respectively), and 1.5x the interquartile range (whiskers). ***$P < 2.51 \times 10^{-96}$; **$P < 5.83 \times 10^{-37}$; *$P < 9.23 \times 10^{-9}$; a two-sided paired t-test. **e,** Analysis of day 6 CREST knockout differentially expressed genes (DEGs) in mRNA-seq data from human iPSC-derived motor neurons with ALS mutations.55 Gene set enrichment analysis (GSEA) plots showing down- and up-regulated genes in day 6 KO cells in the context of ALSC9orf72 mutations (left) and ALSFUS mutations (right) compared with control motor neurons.

b. Only two of the three citations in line 65 identify CREST mutations in ALS patients. Moreover, the known mutations have been identified in just a few patients. Please revise the language around the disease relevance to avoid overstating the findings.

Response to Point #2b:
We thank the reviewer for this comment. We have now revised the language accordingly. We also removed the reference 27 below, which is not based on an ALS patients' study.
(Ref 27: Tibshirani, M. et al. Dysregulation of chromatin remodelling complexes in amyotrophic lateral sclerosis. Hum Mol Genet 26, 4142-4152 (2017). https://doi.org:10.1093/hmg/ddx301)

3. Acetyltransferase specificity
a. The manuscript only investigates CBP/p300 involvement. Are these the only acetyltransferases expressed in these neurons? If others are present, e.g. Tip60, please justify why they are not examined or discuss their potential roles.

Response to Point #3:
We thank the reviewer for suggesting this experiment. We confirmed that the Tip60 (KAT5) acetyltransferase is also expressed in day 6 motor neurons. To examine potential roles of Tip60 in motor neurons, we performed ChIP-seq for Tip60 in day 6 WT and KO cells. We revealed that Tip60 binds at neither CREST-bound nor CREST-unbound distal regions; instead, it localizes to CREST-unbound promoter regions (Supplementary Fig. 7b). These findings indicated that CBP, rather than p300 or Tip60, exhibits specificity for CREST-bound distal regions in day 6 motor neurons. Interestingly, CBP binding was notably decreased at CREST-bound distal regions in day 6 KO cells (**Fig. 3d**). In contrast, p300 enrichment at these distal regions and Tip60 enrichment at promoters remained unchanged in day 6 KO cells compared to WT cells (Supplementary Fig. 7b), indicating no functional association between CREST binding and Tip60 recruitment.

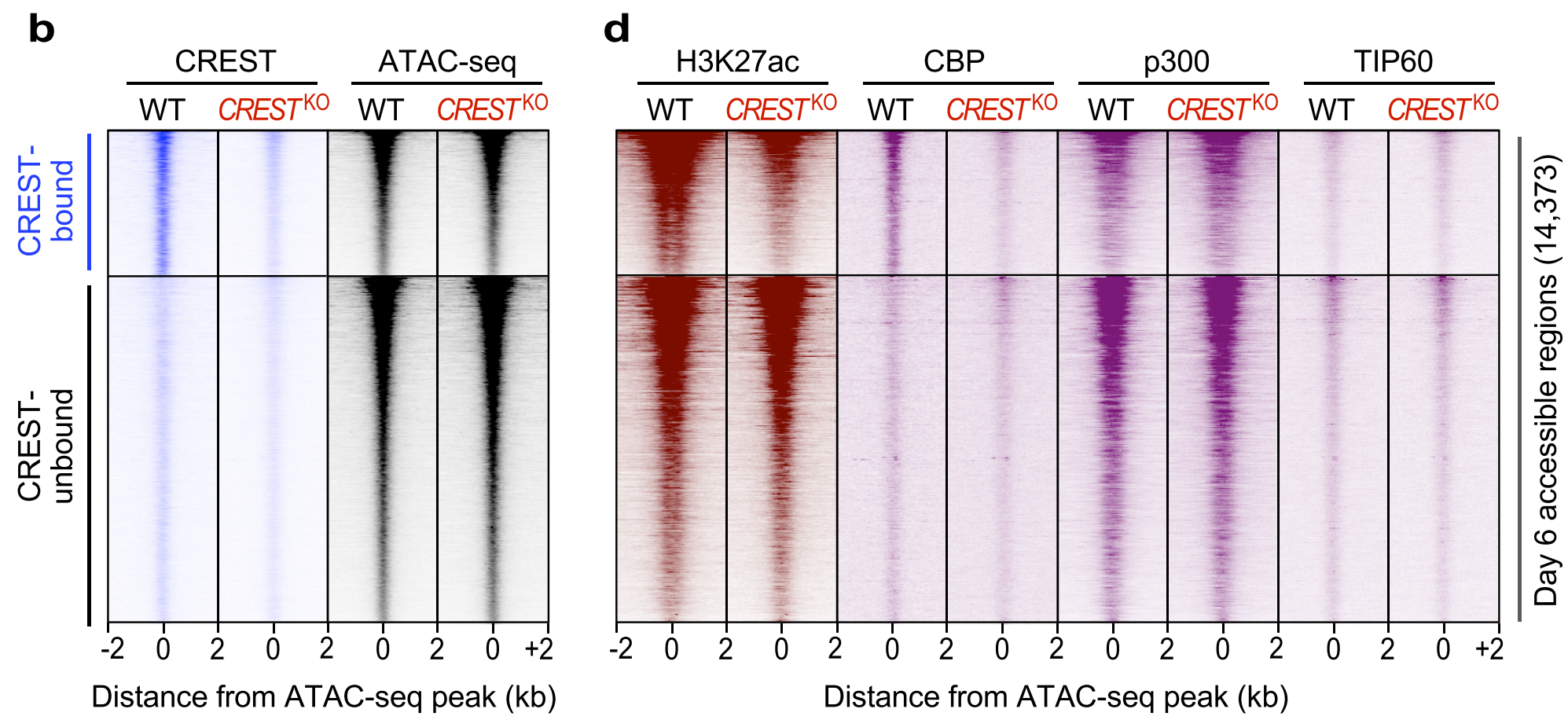

Figure 3 | **b,** Genomic mapping of ChIP-seq CREST and ATAC-seq in day 6 WT motor neurons and day 6 KO cells. 14,373 highly accessible distal cis-regulatory regions were identified from ATAC-seq in day 6 WT motor neurons, grouped by CREST-bound (n = 4221) and -unbound distal regions (n = 10,152). All data were plotted relative to the ATAC-seq peak midpoints, sorted, and ordered by ATAC-seq intensity in day 6 WT motor neurons (Supplementary Data 6). **d,** ChIP-seq mapping of H3K27ac and histone acetyltransferases (CBP, p300, and TIP60) in day 6 WT motor neurons and day 6 KO cells at the accessible distal cis-regulatory regions identified in panel (b). All data were plotted relative to 14,373 ATAC-seq peak midpoints in the same order as shown in panel (b).

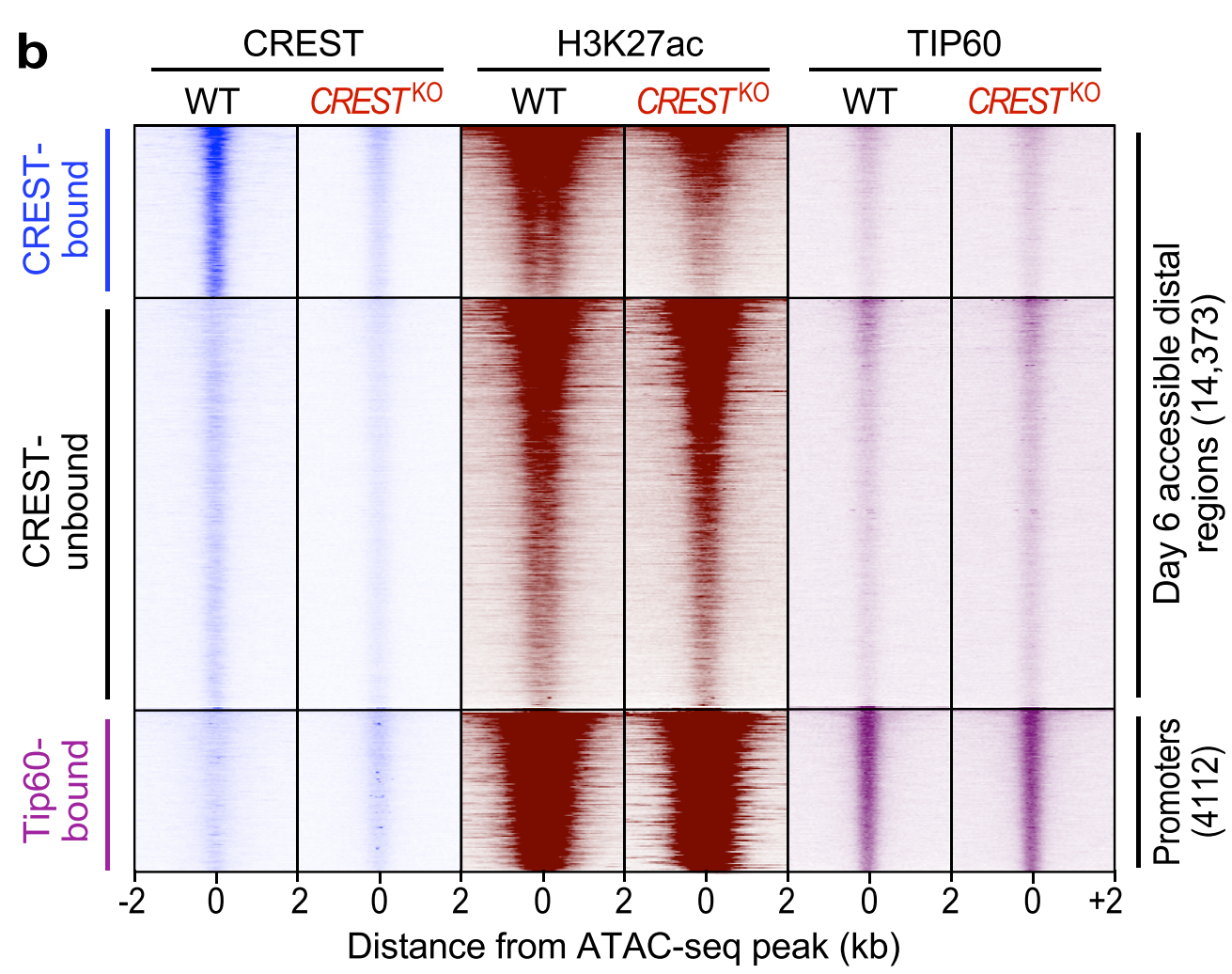

Supplementary Fig. 7b | ChIP-seq intensity of CREST, H3K27ac, and Tip60 in day 6 WT motor neurons at 14,373 accessible distal regions and 4112 promoter regions, bound by Tip60. All data were plotted relative to the ATAC-seq midpoints, sorted, and ordered by ATAC-seq intensity, as shown in Fig. 3b,d, except the 4112 regions.

4.CREST ChIP signal in KO cells

a. The authors should explain the presence of CREST ChIP signal in KO cells. Is this background noise, non-specific antibody binding, or incomplete knockout?

Response to Point #4:

We thank the reviewer for this comment. Please see our previous response to Reviewer 4's comment - Point #1c.

5. Evidence for direct protein interactions

a. Many mechanistic claims rely on co-occupancy in ChIP-seq. Without biochemical validation (co-IP, PLA, mass spec, etc.) the proposed CREST-BRM-TF complexes remain speculative. Consider

changing the language or incorporating direct interaction data if possible.

Response to Point #5:
We thank the reviewer for suggesting this experiment. We also utilized co-immunoprecipitation (co-IP) to determine whether CREST-containing BAF complexes interact with ONECUT1 and NGN2 via protein-protein interactions. We confirmed the physical interactions between the BAF complexes (BRM) and these TFs in day 6 motor neurons (**Fig. 5h**). These results support the conclusion that CREST is recruited to developmentally regulated neuronal enhancers via interactions with stage-specific neuronal TFs. Additionally, we used co-IP to determine whether the CREST knockout disrupted protein-protein interactions within the BAF complex. We found that the interaction between BAF170 and BRM was unaltered by CREST knockout, and BAF53b did not directly interact with BRM in motor neurons (Supplementary Fig. 18). Thus, we confirmed that while *CREST* KO downregulates specific neuronal BAF subunits, *CREST* KO does not disrupt their structural assembly within motor neuron BAF complexes.

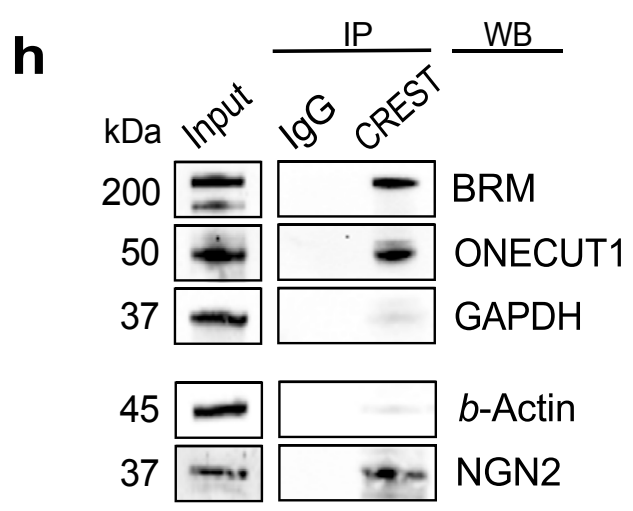

Figure 5h | Biochemical demonstration of the interactions of CREST with BRM, ONECUT1, and NGN2 in day 6 WT motor neurons. Immunoprecipitation (IP) of day 6 cell lysates was performed using an anti-CREST antibody, followed by western blot (WB) analysis for BRM, ONECUT1, NGN2, and negative controls (GAPDH and β-Actin). The detected bands correspond to ~200 kDa for BRM, ~50 kDa for ONECUT1, ~37 kDa for NGN2 and GAPDH, and ~45 kDa for β-Actin. The input consists of 25 μg of nuclear extract; normal IgG serves as a negative control for the IP.

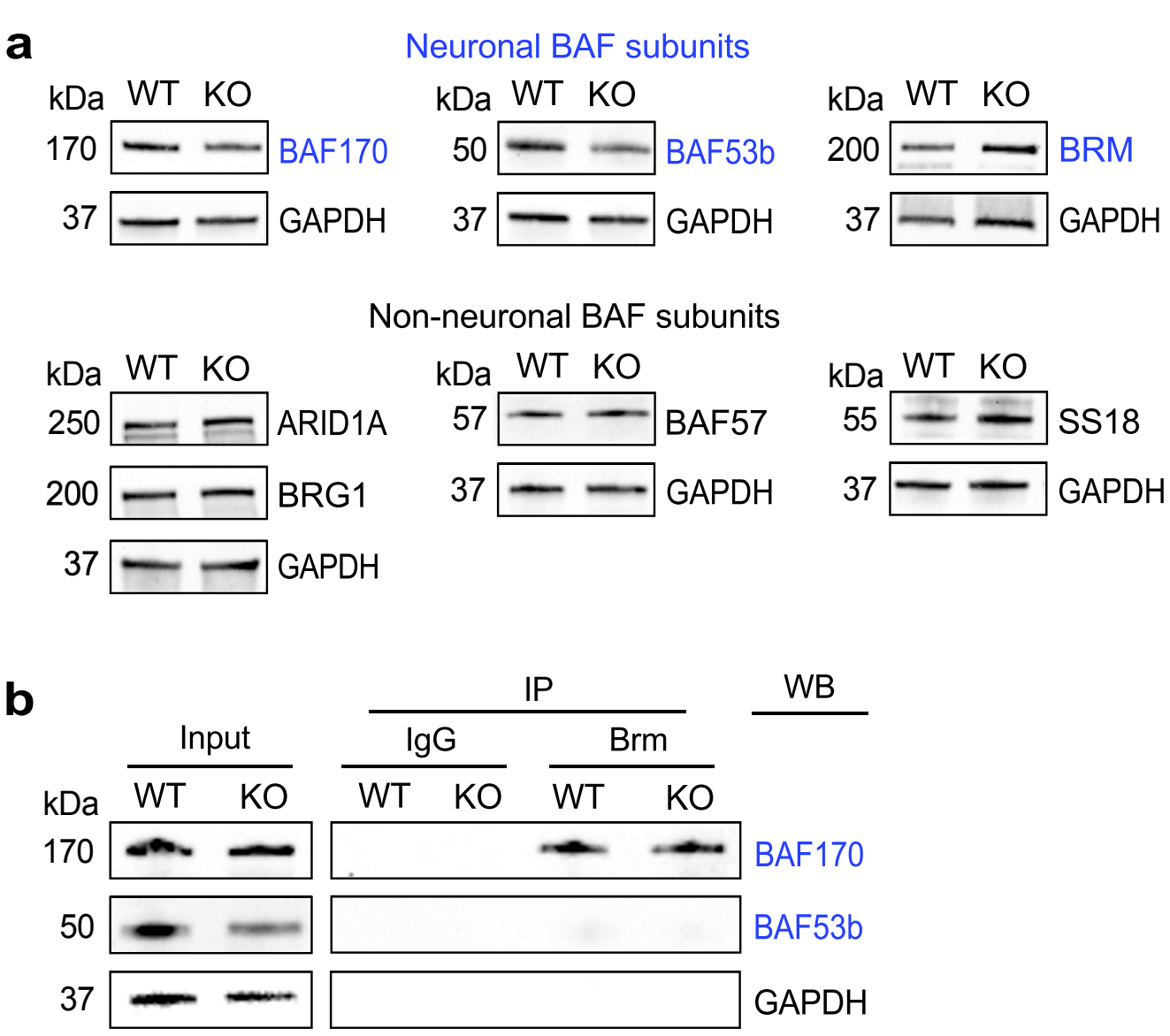

Supplementary Fig. 18 | CREST knockout downregulates the protein levels of specific neuronal BAF subunits in day 6 differentiated cells. **a,** Western blot analysis confirms that the CREST knockout reduces the protein levels of two neuronal BAF subunits, BAF170 and BAF53b, in day 6 differentiated cells. However, it does not affect the protein levels of another neuronal BAF subunit, BRM, or the non-neuronal BAF subunits ARID1A, BRG1, BAF57, and SS18. The detected bands correspond to ~170 kDa for BAF170, ~50 kDa for BAF53b, ~200 kDa for BRM and BRG1, ~250 kDa for Arid1a, ~57 kDa for BAF57, ~55 kDa for SS18, and ~37 kDa for GAPDH (used as a loading control). **b,** Biochemical demonstration of the interactions of BRM with BAF170 and BAF53b in day 6 WT and day 6 KO cells. Immunoprecipitation (IP) of day 6 cell lysates was performed using an anti-BRM antibody, followed by western blot

(WB) analysis for BAF170, BAF53b, and GAPDH (used as a negative control). The detected bands correspond to ~170 kDa for BAF170, ~50 kDa for BAF53b, and ~37 kDa for GAPDH. The input consists of 25 µg of nuclear extract; normal IgG served as a negative control for the IP.

Minor Comments

• Line 330: "CREST-bound regions become accessible" implies causality. Rephrase to clarify that CREST correlates with accessibility but is not directly responsible for it.
Response to Comment:
We thank the reviewer for this suggestion. We have now rephrased the text to clarify our previous statement. Specifically, we have revised the original sentence:
"CREST-bound regions become accessible in a neuron-stage specific manner" to "CREST binding correlates with chromatin accessibility, although chromatin accessibility is not dependent on CREST binding". (Revised Line 312)

• Lines 351 & 498: The implication that Brg1 is not involved in neuronal BAF complexes is misleading. Clarify that Brg1 may play roles in other neuronal contexts or subtypes but not in these.
Response to Comment:
We thank the reviewer for this comment. We have now revised this statement accordingly. (Revised Lines 328)

• Lines 396-399: This section reads like a laundry list. Please rewrite to aid in flow and clarity.
Response to Comment:
We thank the reviewer for this comment. We have now revised this statement accordingly. (Revised Lines 357-359)

• Lines 489-490: Claims about Brm-CREST recruitment by Onecut1 and Ngn2 are based on motif enrichment, not functional or binding evidence. Please adjust the language to reflect this.
Response to Comment:
We thank the reviewer for this comment. We have now revised this language accordingly. (Revised Lines 458-459)

• Line 499: Claims CTCF and p300 are non-neuronal transcription factors. P300 is a transcription coactivator, not a transcription factor and CTCF is certainly present in neurons. Please revise.
Response to Comment:
We thank the reviewer for this comment. We have now revised this statement accordingly. (Revised Lines 465-466)

• Figure 7: Shows multiple BAF complex cartoons in the same protein complex.
Response to Comment:
We thank the reviewer for this comment. We have now revised the cartoons accordingly.

• Contributions: Remove placeholder text “will be added after reviews from co-authors”

Response to Comment:

We thank the reviewer for this comment. We have now removed this text.

REVIEWER COMMENTS

Reviewer #1 (Remarks to the Author):

The authors have substantially improved the manuscript and addressed many of my previous concerns. The revised analyses support the conclusion that CREST functions at neuronal enhancers that are already engaged in long-range chromatin interactions, rather than establishing chromatin accessibility or chromatin looping de novo. However, I remain concerned about the interpretation of the newly added western blot data for the BAF complex subunits. The reported reductions in BAF170 and BAF53b protein levels are not readily apparent from the presented blots. In particular, the decrease in BAF170 appears quite modest. Quantitative densitometric analyses from multiple biological replicates, together with statistical evaluation, are necessary to support the claim that these proteins are reduced.

Response to Reviewer #1:

We thank the reviewer for this comment. To address the concern about potential protein level changes and quantification in neuronal BAF subunits, we performed additional western blotting for BAF53b, BAF170, and several other BAF subunits. Quantitative analysis showed a significant reduction in BAF53b protein levels upon *CREST* knockout ($P = 3.92 \times 10^{-3}$), whereas BAF170 and non-neuronal BAF subunits showed no significant change (Supplementary Fig. 18a). These results suggest that while *CREST* knockout may downregulate the protein levels of specific neuronal BAF subunits, *CREST* knockout does not disrupt their overall structural assembly within motor neuron BAF complexes.

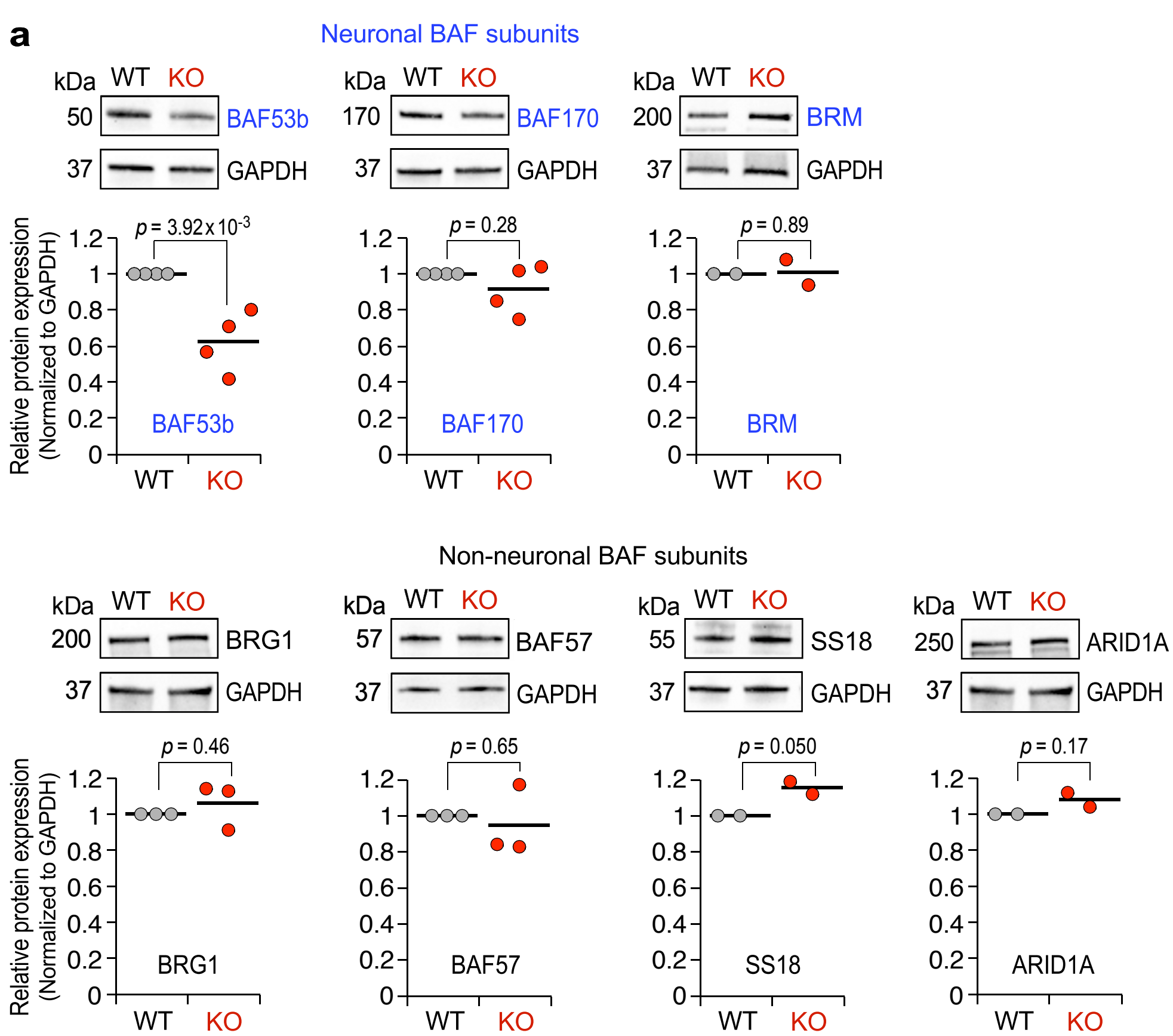

Supplementary Fig. 18a | Western blot analysis of three neuronal BAF subunits (BAF53b, BAF170, and BRM) and four non-neuronal BAF subunits (BRG1, BAF57, SS18, and ARID1A) in day 6 WT and CREST KO cells. Detected bands correspond to ~50 kDa (BAF53b), ~170 kDa (BAF170), ~200 kDa (BRM and BRG1), ~57 kDa (BAF57), ~55 kDa (SS18), ~250 kDa (ARID1A), and ~37 kDa (GAPDH, loading control). ARID1A and BRG1 were resolved on the same gel, sharing the same GAPDH loading control. Horizontal lines in the dot plots represent the average. P-values were calculated using a two-sided, two-sample Student's *t*-test. A representative immunoblot from biological replicate 1 is shown here. All densitometric intensity were calculated using ImageJ and are reported in Supplementary Data 9. Target protein band intensities were normalized to their corresponding GAPDH loading control intensities within each biological replicate.

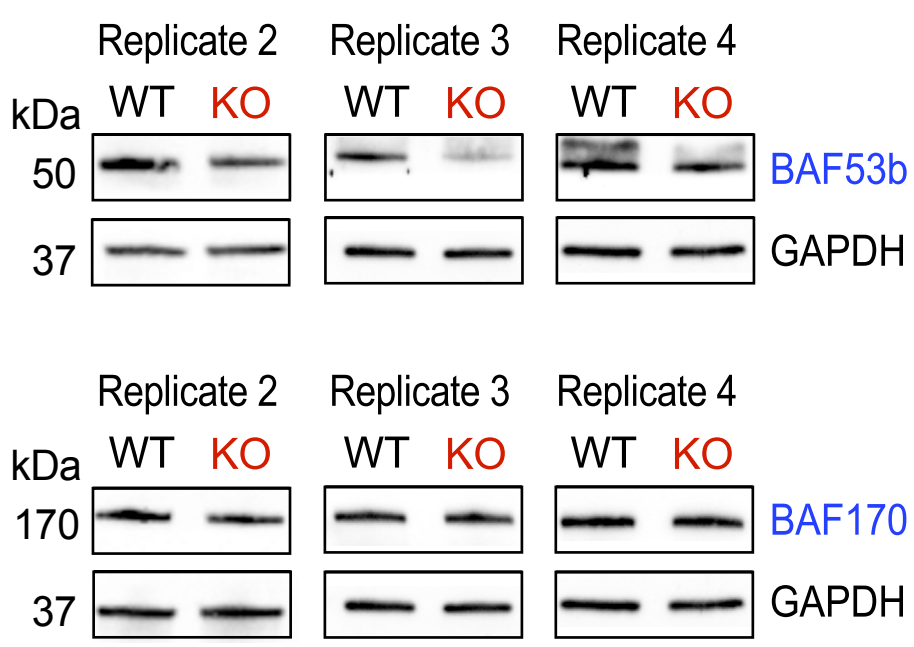

Supplementary Data 9 | Additional biological replicates for Western blot analysis of BAF53b and BAF170. Replicate 1 was shown in Supplementary Fig. 18a.

Reviewer #2 (Remarks to the Author):

I don't have any further comments. I am satisfied by the authors' responses.

Reviewer #3 (Remarks to the Author):

I co-reviewed this manuscript with one of the reviewers who provided the listed reports. This is part of the Nature Communications initiative to facilitate training in peer review and to provide appropriate recognition for Early Career Researchers who co-review manuscripts.

Reviewer #4 (Remarks to the Author):

The revised manuscript has addressed all the concerns I had.

REVIEWERS' COMMENTS

Reviewer #1 (Remarks to the Author):

The authors have addressed my remaining concern by providing additional quantitative analyses and appropriately revising their interpretation based on the new data. The updated results do not materially affect the overall conclusions of the study. I have no further concerns and support acceptance of the manuscript in its current form.

Response to Reviewer #1:
We thank the reviewer for all the comments to improve our manuscript.
