# Supplementary material for: CREST functions as a molecular bridge between neuron-specific enhancers and epigenetic regulators in mature motor neurons
Source: Nat Commun. 2026 Oct 10;17:11520. doi: 10.1038/s41467-026-78396-x (PMC13745572; doi:10.1038/s41467-026-78396-x)
Supplement: Supplementary file 3 [file 41467_2026_78396_MOESM3_ESM.pdf]

nature portfolio

Corresponding author(s): Ho Sung Rhee
Last updated by author(s): Aug 23, 2026

Reporting Summary

Nature Portfolio wishes to improve the reproducibility of the work that we publish. This form provides structure for consistency and transparency in reporting. For further information on Nature Portfolio policies, see our Editorial Policies and the Editorial Policy Checklist.

Statistics

For all statistical analyses, confirm that the following items are present in the figure legend, table legend, main text, or Methods section.

n/a	Confirmed	
☐	☒	The exact sample size (n) for each experimental group/condition, given as a discrete number and unit of measurement
☐	☒	A statement on whether measurements were taken from distinct samples or whether the same sample was measured repeatedly
☐	☒	The statistical test(s) used AND whether they are one- or two-sided<br>*Only common tests should be described solely by name; describe more complex techniques in the Methods section.*
☒	☐	A description of all covariates tested
☐	☒	A description of any assumptions or corrections, such as tests of normality and adjustment for multiple comparisons
☐	☒	A full description of the statistical parameters including central tendency (e.g. means) or other basic estimates (e.g. regression coefficient) AND variation (e.g. standard deviation) or associated estimates of uncertainty (e.g. confidence intervals)
☐	☒	For null hypothesis testing, the test statistic (e.g. F, t, r) with confidence intervals, effect sizes, degrees of freedom and P value noted<br>*Give P values as exact values whenever suitable.*
☒	☐	For Bayesian analysis, information on the choice of priors and Markov chain Monte Carlo settings
☒	☐	For hierarchical and complex designs, identification of the appropriate level for tests and full reporting of outcomes
☐	☒	Estimates of effect sizes (e.g. Cohen's d, Pearson's r), indicating how they were calculated

Our web collection on statistics for biologists contains articles on many of the points above.

Software and code

Policy information about availability of computer code

Data collection	N/A
Data analysis	RNA STAR (v2.7.0), DESeq2 software (v1.32.0), Salmon (v1.10.0), Cufflinks (v2.2.1), Trim Galore (v0.6.7), Bowtie2 (v2.4.5), bedtools (v2.30.0), MACS2 (v2.2.9.1), DiffBind (v3.12.0), IGV (igv.org; v2.8.12), Java Treeview (v1.1.6), MEME (meme-suite.org; v5.5.8), BWA-mem (v0.7.17), Picard (v2.6.0), Juicer (v), Domain Caller (v1.0), HiCRep (v1.6.0), LIMMA (v3.50.3)

For manuscripts utilizing custom algorithms or software that are central to the research but not yet described in published literature, software must be made available to editors and reviewers. We strongly encourage code deposition in a community repository (e.g. GitHub). See the Nature Portfolio guidelines for submitting code & software for further information.

Data

Policy information about availability of data

All manuscripts must include a data availability statement. This statement should provide the following information, where applicable:

- Accession codes, unique identifiers, or web links for publicly available datasets
- A description of any restrictions on data availability
- For clinical datasets or third party data, please ensure that the statement adheres to our policy

Raw and processed sequencing datasets have been deposited in the National Center for Biotechnology Information (NCBI) Gene Expression Omnibus (GEO) under the accession numbers GSE234503, GSE291506, and GSE326223 for RNA-seq, GSE291240, GSE291396, and GSE326333 for ChIP-seq, GSE291510 for ATAC-seq, and GSE281024 for Hi-C.

Research involving human participants, their data, or biological material

Policy information about studies with human participants or human data. See also policy information about sex, gender (identity/presentation), and sexual orientation and race, ethnicity and racism.

Reporting on sex and gender	No human participants are involved in this study.
Reporting on race, ethnicity, or other socially relevant groupings	N/A
Population characteristics	N/A
Recruitment	N/A
Ethics oversight	N/A

Note that full information on the approval of the study protocol must also be provided in the manuscript.

Field-specific reporting

Please select the one below that is the best fit for your research. If you are not sure, read the appropriate sections before making your selection.

☒ Life sciences ☐ Behavioural & social sciences ☐ Ecological, evolutionary & environmental sciences

For a reference copy of the document with all sections, see nature.com/documents/nr-reporting-summary-flat.pdf

Life sciences study design

All studies must disclose on these points even when the disclosure is negative.

Sample size	Sample sizes were chosen as standard in the field or depend on the availability of datasets. A minimum of n=2 biological replicates were used.
Data exclusions	No data were excluded.
Replication	All experimental and genomic datasets were consistently reproduced in independent biological replicates.
Randomization	No randomization was required for motor neuron differentiation. This was not feasible given mouse embryonic stem cell-derived differentiation and genomic data production here.
Blinding	We are not blinded to samples during experiments. This was not feasible given mouse embryonic stem cell-derived differentiation and genomic data production here.

Reporting for specific materials, systems and methods

We require information from authors about some types of materials, experimental systems and methods used in many studies. Here, indicate whether each material, system or method listed is relevant to your study. If you are not sure if a list item applies to your research, read the appropriate section before selecting a response.

Materials & experimental systems

n/a	Involved in the study	
☐	☒	Antibodies
☐	☒	Eukaryotic cell lines
☒	☐	Palaeontology and archaeology
☒	☐	Animals and other organisms
☒	☐	Clinical data
☒	☐	Dual use research of concern
☒	☐	Plants

Methods

n/a	Involved in the study	
☐	☒	ChIP-seq
☒	☐	Flow cytometry
☒	☐	MRI-based neuroimaging

Antibodies

Antibodies used	For western blot (WB): anti-CREST (Proteintech, 12439-1-AP, 1:1000 used), anti-GAPDH (Cell Signaling, 2118, 1:1000 used), anti-MAP2 (Cell Signaling, 4542S, 1:1000 used), anti-SS18 (Cell Signaling, 21792, 1:1000 used), anti-ChAT (Millipore Sigma, AB144P, 1:1000 used), anti-BAF170 (Cell Signaling, 12760, 1:1000 used), anti-BAF53b (Antibodies Incor., 75-311, 1:1000 used), anti-BRM (Bethyl Lab, A301-015A, 1:1000 used), anti-ARID1A (Cell Signaling, 12354S, 1:1000 used), anti-BRG1 (Abcam, ab110641, 1:1000 used), anti-BAF57 (Bethyl Lab, A300810AT, 1:1000 used), anti-mouse secondary antibody (Proteintech, SA00001-1, lot# NA, 1:10000 used), anti-rabbit secondary antibody (Santa Cruz, sc-2357; lot# E0819, 1:10000 used).

For immunefluorescence (IF) staining: anti-CREST (ProteinTech, 12439-1-AP, 1 ug/mL used), anti-HB9 (DSHB, 81.5C10, 1:1000 used), anti-ISL1 (Neuromics, GT15051, 1 ug/mL used), anti-H3K27ac (Abcam, ab4729, 1 ug/mL used), anti-MAP2 (Cell Signaling, 4542S, 1 ug/mL used), anti-TUBB3 (Sigma, T2200-200ul, 1 ug/mL used), anti-goat IgG Alexa Fluor 488 (Thermo, A11055; lot# 1942238,1:1000 used), anti-mouse IgG Alexa Fluor 555 (Thermo, A31570; lot# 1905844, 1:1000 used), and anti-rabbit IgG Alexa Fluor 647 (Thermo, A31573; lot# 1964354, 1:1000 used), anti-rabbit secondary antibody (Thermo, A31573, 1:1000 used).

For ChIP-seq: anti-CREST (ProteinTech, 12439-1-AP, 4 ug used), anti-CBP (Cell Signaling, 7389S, 3 ug used), anti-p300 (Abcam, ab275378, 3 ug used), anti-TIP60 (ProteinTech, 10827-1-AP, 3 ug used), anti-IgG (Cell Signaling, 2729, 3 ug used), anti-BRM (Bethyl, A301-015A, 5 ug used), anti-BRG1 (Abcam, ab110641, 3 ug used), anti-H3K27ac (Abcam, ab4729, 3 ug used), anti-H3K4me1 (Abcam, ab8895, 3 ug used), anti-H3K4me3 (Abcam, ab8580, 3 ug used), anti-H3K27me3 (Abcam, ab6002, 3 ug used), anti-CTCF (Millipore, 07-729, 3 ug used), anti-RPB1 (Cell Signaling, 2629, 3 ug used).

For co-immunoprecipiation (co-IP): anti-CREST (ProteinTech, 12439-1-AP, 4 ug used), anti-BRM (Bethyl Lab, A301-015A, 1:1000 used), anti-ONECUT1 (Santa Cruz, sc-376167, 1:1000 used), anti-NGN2 (Cell Signaling, 13144, 1:1000 used), anti-β-Actin (Santa Cruz, sc-47778, 1:1000 used), anti-BAF170 (Cell Signaling, 12760, 1:1000 used), anti-BAF53b (Antibodies Incor., 75-311, 1:1000 used), anti-GAPDH (Cell Signaling, 2118, 1:1000 used), anti-mouse secondary antibody (Proteintech, SA00001-1, lot# NA, 1:10000 used), anti-rabbit secondary antibody (Santa Cruz, sc-2357; lot# E0819, 1:10000 used).

Validation

anti-CREST (Proteintech, 12439-1-AP; lot#, 00042079); species reactivity: human, mouse, rat; application: WB, IHC, IF/ICC, IF-P, IP, ELISA; application in this study: WB; validation statement: 12439-1-AP targets CREST in WB, IHC, IF/ICC, IF-P, IP, CoIP, ChIP, ELISA applications and shows reactivity with human, mouse, rat samples.

anti-GAPDH (Cell Signaling, 2118; lot# 082020-14); species reactivity: human, mouse, rat, monkey, bovine, pig; recommended applications: WB, IHC, IF, Flow Cyt; application in this study: WB; validation statement: GAPDH (14C10) Rabbit Monoclonal Antibody detects endogenous levels of total GAPDH protein.

anti-MAP2 (Cell Signaling, 4542S; lot# 032019-4); species reactivity: human, mouse, rat, monkey; recommended applications: WB, IHC, IF; application in this study: WB; validation statement: MAP2 Antibody detects endogenous levels of all isoforms of MAP2 total protein. This antibody does not cross-react with tau.

anti-SS18 (Cell Signaling, 21792; lot# 092029-1); species reactivity: human, mouse, rat, monkey; recommended applications: WB, IHC, IF, ChIP, CUT&RUN; application in this study: WB; validation statement: For optimal ChIP and ChIP-seq results, use 10 µl of antibody and 10 µg of chromatin (approximately 4 x 106 cells) per IP.

anti-ChAT (Millipore Sigma, AB144P; lot# 4254299); species reactivity: uman, opossum, guinea pig, rat, mouse, zebrafish, avian, monkey, chicken; recommended applications: ICC, IHC, WB; application in this study: WB; validation statement: THE JOURNAL OF COMPARATIVE NEUROLOGY 474:75–107 (2004).

anti-BAF170 (Cell Signaling, 12760; lot# 052030-2); species reactivity: human, mouse, rat, monkey; recommended applications: WB, IP, ChIP, CUT&RUN; application in this study: WB; validation statement: SMARCC2/BAF170 (D8O9V) Rabbit Monoclonal Antibody recognizes endogenous levels of total SMARCC2/BAF170 protein.

anti-BAF53b (Antibodies Inc., 75-311; lot# 449-4AK-23); species reactivity: human, mouse, rat; recommended applications: ELISA, ICC, IHC, WB; application in this study: WB; validation statement: Each new lot of antibody is quality control tested by IHC on either rat or mouse brain and confirmed to give the expected staining pattern.

anti-BRM (Bethyl Lab, A301-015A; lot# 2); species reactivity: human; recommended applications: IP, WB; application in this study: WB; validation statement: Western blots of immunoprecipitates are performed using an anti-Rabbit IgG Light Chain HRP-conjugated antibody with 5% Normal Pig Serum (Cat. No. S100-020) added to the blocking buffer.

anti-ARID1A (Cell Signaling, 12354S; lot# 022025-4); species reactivity: human, mouse, rat, monkey; recommended applications: WB, IHC, ChIP; application in this study: WB; validation statement: ARID1A/BAF250A (D2A8U) Rabbit Monoclonal Antibody recognizes endogenous levels of total ARID1A/BAF250A protein. This antibody also cross-reacts with proteins of unknown origin at 65 kDa.

anti-BRG1 (Abcam, ab110641; lot# 1141327-3); species reactivity: human, mouse, rat; recommended applications: WB, Flow Cyt, IP, IHC, ICC/IF; application in this study: WB; validation statement: The specificity of Anti-BRG1 antibody [EPNCIR111A] (ab110641) has been confirmed by Western Blot testing in BRG1 knockout HEK-293T cells.

anti-BAF57 (Bethyl Lab, A300810AT; lot#, 2); species reactivity: mouse, human; recommended applications: IHC, IP, WB; application in this study: WB; validation statement: Western blot of immunoprecipitates performed using Normal Pig Serum (Cat. No. S100-020), Goat anti-Rabbit Light Chain HRP Conjugate (Cat. No. A120-113P) and 4-20% SDS-PAGE.

anti-HB9 (DSHB, 81.5C10; lot# NA); species reactivity: fish, human, mouse, xenopus, zebrafish; recommended applications: IHC, IF, ICC, WB; application in this study: IF; validation statement: 81.5C10 was deposited to the DSHB by Jessell, T.M. / Brenner-Morton, S. (DSHB Hybridoma Product 81.5C10)

anti-ISL1 (Neuromics, GT15051; lot# NA); species reactivity: mouse, human; recommended applications: IHC, WB, ELISA; application in this study: IF; validation statement: This antibody can be used at 0.1 - 0.2 µg/mL with the appropriate secondary reagents to detect human Islet-1 in western blotting.

anti-H3K27ac (Abcam, ab4729; lot# 1059037-6); species reactivity: cow, human, mouse, rat; recommended applications: WB, IP, IHC-P, ICC/IF, ChIP, PepArr; application in this study: IF; validation statement: From Jan 2024, QC testing of replenishment batches of this

polyclonal changed. All tested and expected application and reactive species combinations are still covered by our Abcam product promise.

anti-TUBB3 (Sigma, T2200-200ul; lot# NA); species reactivity: rat, human, mouse; recommended applications: IF, IP, WB; application in this study: IF; validation statement: for immunofluorescence: 10-20 µg/mL using rat PC12 cells, for western blot: 0.2-0.4 µg/mL using whole extracts of mouse brain or cultured human neuroblastoma SH-SY5Y cells

anti-CBP (Cell Signaling, 7389S; log# NA); species reactivity: human, mouse, rat, monkey; recommended applications: WB, IP, IHC, IF, ChIP; application in this study: ChIP; validation statement: Western blot analysis of extracts from control HEK293T cells (Lane 1) or HEK293T cells with a targeted mutation in the gene encoding CBP using CBP (D6C5) Rabbit mAb or GAPDH.

anti-p300 (Abcam, ab275378; lot# 1074860-31); species reactivity: human, mouse, rat; recommended applications: WB, Flow Cyt, IP, ICC/IF, ChIP; application in this study: ChIP; validation statement: Immunofluorescent analysis of 4% Paraformaldehyde-fixed, 0.1% Triton X-100 permeabilized HeLa cells labelling KAT3B/p300 with ab275378 at 1/100 (4.77 ug/ml) dilution.

anti-TIP60 (ProteinTech, 10827-1-AP; lot # 00138365); species reactivity: human, mouse; recommended applications: WB, IHC, ELISA; application in this study: ChIP; validation statement: WB result of KAT5 antibody (10827-1-AP; 1:1000; incubated at room temperature for 1.5 hours) with sh-Control and sh-KAT5 transfected HepG2 cells.

anti-IgG (Cell Signaling, 2729; lot# 11); species reactivity: mouse, human; recommended applications: IP, ChIP, CUT&Tag; application in this study: ChIP; validation statement: Immunoprecipitation of 4E-BP2 from C2C12 cell extracts using Normal Rabbit IgG #2729 or 4E-BP2 Antibody #2845.

anti-H3K4me1 (Abcam, ab8895; lot# GR3442591-2); species reactivity: cow, human, mouse, rat; recommended applications: WB, IP, IHC, ICC/IF, ChIP; application in this study: ChIP; validation statement: ab8895 staining Histone H3 (mono methyl K4) in HeLa cells. The cells were fixed with 4% paraformaldehyde (10 min), permeabilized with 0.1% PBS-Triton X-100 for 5 minutes.

anti-H3K4me3 (Abcam, ab8580; lot# GR3362386-3); species reactivity: cow, human; recommended applications: WB, IP, IHC-P, ICC/IF, ChIP, PepArr; application in this study: ChIP; validation statement: Chromatin was prepared from U-2 OS (Human bone osteosarcoma epithelial cell line) cells according to the Abcam X-ChIP protocol.

anti-H3K27me3 (Abcam, ab6002; lot# NA); species reactivity: cow, human, mouse; recommended applications: IP, IHC- Wmt, ICC/IF, ELISA, ChIP; application in this study: ChIP; validation statement: Chromatin was prepared from Hela cells according to the Abcam X-ChIP protocol. Cells were fixed with formaldehyde for 10min.

anti-CTCF (Millipore, 07-729; lot# 3515588); species reactivity: mouse, human, canine, rat, primate; recommended applications: ChIP, WB; application in this study: ChIP; validation statement: An independent laboratory has confirmed that this antibody immunoprecipitates CTCF-bound chromatin from WEHI B-cells.

anti-RPB1 (Cell Signaling, 2629; lot# 062025-4); species reactivity: human, mouse, rat, monkey; recommended applications: WB, IP, ChIP, CUT&RUN, CUT&Tag; application in this study: ChIP; validation statement: Western blot analysis of extracts from various cell lines using Rpb1 CTD (4H8) Mouse mAb.

anti-ONECUT1 (Santa Cruz, sc-376167; lot# J2417); species reactivity: mouse, human; recommended applications: IP, WB, IHC, ELISA; application in this study: co-IP; validation statement: HNF-6 Antibody (G-10): sc-376167. Western blot analysis of HNF-6 expression in non-transfected: sc-117752 (A) and mouse HNF-6 transfected: sc-120850 (B) 293T whole cell lysates.

anti-NGN2 (Cell Signaling, 13144; lot# 052030-3); species reactivity: human, mouse, rat; recommended applications: WB; application in this study: co-IP; validation statement: Western blot analysis of mouse brain and fetal rat brain using Neurogenin 2 (D2R3D) Rabbit mAb.

anti-β-Actin (Santa Cruz, sc-47778; lot# H1420); species reactivity: mouse, human; recommended applications: IP, WB, IHC, ELISA; application in this study: co-IP; validation statement: β-Actin Antibody (C4) Alexa Fluor 790: sc-47778 AF790. Direct near-infrared western blot analysis of β-Actin expression in HeLa (A), NIH/3T3 (B) and KNRK (C) whole cell lysates.

Eukaryotic cell lines

Policy information about cell lines and Sex and Gender in Research

Cell line source(s)	The mouse embryonic stem (ES) cell line was derived from the inbred mouse strain 129/Ola ES-E14 male embryo.
Authentication	During routine cell culture, parental lines were authenticated by monitoring key phenotypic features, including embryonic stem (ES) cell morphology, embryoid body formation, and media color transitions. The baseline characteristics of this ES cell line and its differentiated motor neuron derivatives have been described previously. To confirm stage-specific expression of molecular markers throughout motor neuron differentiation, we performed systematic immunostaining and Western blotting experiments. Sex was not considered as a biological variable in this study, as the mouse embryonic stem cell lines utilized were evaluated strictly for baseline, non-sex-specific mechanisms of gene expression and protein-DNA interactions.
Mycoplasma contamination	All cell lines were tested negative for mycoplasma contamination.
Commonly misidentified lines (See ICLAC register)	No commonly misidentified cell lines were used in this study.

Plants

Seed stocks | N/A

Novel plant genotypes | N/A

Authentication | N/A

ChIP-seq

Data deposition

☒ Confirm that both raw and final processed data have been deposited in a public database such as GEO.

☒ Confirm that you have deposited or provided access to graph files (e.g. BED files) for the called peaks.

Data access links
May remain private before publication.

The raw and processed sequencing data generated in this study have been deposited in the National Center for Biotechnology Information (NCBI) Gene Expression Omnibus (GEO) under the accession codes GSE234503 [https://www.ncbi.nlm.nih.gov/geo/query/acc.cgi?acc=GSE234503], GSE291506 [https://www.ncbi.nlm.nih.gov/geo/query/acc.cgi?acc=GSE291506], and GSE326223 [https://www.ncbi.nlm.nih.gov/geo/query/acc.cgi?acc=GSE326223] for RNA-seq, GSE291240 [https://www.ncbi.nlm.nih.gov/geo/query/acc.cgi?acc= GSE291240], GSE291396 [https://www.ncbi.nlm.nih.gov/geo/query/acc.cgi?acc= GSE291396], and GSE326333 [https://www.ncbi.nlm.nih.gov/geo/query/acc.cgi?acc= GSE326333]for ChIP-seq, GSE291510 [https://www.ncbi.nlm.nih.gov/geo/query/acc.cgi?acc= GSE291510] for ATAC-seq, and GSE281024 [https://www.ncbi.nlm.nih.gov/geo/query/acc.cgi?acc= GSE281024] for Hi-C. We also used publicly available data in the NCBI GEO under the accession codes GSE79561 [https://www.ncbi.nlm.nih.gov/geo/query/acc.cgi?acc= GSE79561] for RNA-seq and ChIP-seq, and ATAC-seq in this study.

Files in database submission

The ChIP-seq raw and processed files (fastq, bigwig) generated from this study were submitted to NCIB GEO (GSE291240, GSE291396, and GSE326333).

Genome browser session
(e.g. UCSC)

No genome browser sessions were generated for this study.

Methodology

Replicates

For ChIP-seq datasets, at least two independent biological replicates were used for spinal motor neurons.

Sequencing depth

CREST, wt, rep1; total reads: 32,168,118; uniquely mapped reads: 21,301,398; paired 150 bp
CREST, wt, rep2; total reads: 28,871,548; uniquely mapped reads: 22,570,615; single 100 bp
CREST, KO, rep1; total reads: 15,784,670; uniquely mapped reads: 9,687,950; paired 100 bp
CREST, KO, rep2; total reads: 14,970,876; uniquely mapped reads: 9,602,643; paired 100 bp

Control, wt, rep1; total reads: 19,983,585; uniquely mapped reads: 6,643,322; paired 100 bp
Control, wt, rep2; total reads: 32,684,624; uniquely mapped reads: 15,144,198; paired 150 bp

H3K27ac, wt, rep1; total reads: 36,226,734; uniquely mapped reads: 31,471,443; single 75 bp
H3K27ac, wt, rep2; total reads: 34,982,937; uniquely mapped reads: 28,980,657; paired 150 bp
H3K27ac, KO, rep1; total reads: 12,810,922; uniquely mapped reads: 11,113,450; single 100 bp
H3K27ac, KO, rep2; total reads: 61,266,949; uniquely mapped reads: 53,531,447; single 100 bp

CBP, wt, rep1; total reads: 18,511,209; uniquely mapped reads: 14,486,161; single 100 bp
CBP, wt, rep2; total reads: 49,216,768; uniquely mapped reads: 27,202,388; paired 150 bp
CBP, KO, rep1; total reads: 14,617,228; uniquely mapped reads: 8,712,388; paired 100 bp
CBP, KO, rep2; total reads: 47,304,130; uniquely mapped reads: 31,955,128; paired 150 bp

p300, wt, rep1; total reads: 26,420,266; uniquely mapped reads: 21,889,265; paired 100 bp
p300, wt, rep2; total reads: 25,028,940; uniquely mapped reads: 21,123,443; paired 100 bp
p300, KO, rep1; total reads: 34,204,155; uniquely mapped reads: 27,318,481; paired 100 bp
p300, KO, rep2; total reads: 26,396,853; uniquely mapped reads: 22,170,052; paired 100 bp

TIP60, wt, rep1; total reads: 32,746,139; uniquely mapped reads: 22,440,829; paired 100 bp
TIP60, KO, rep1; total reads: 22,466,927; uniquely mapped reads: 15,942,983; paired 100 bp
TIP60, KO, rep2; total reads: 7,755,053; uniquely mapped reads: 5,495,737; paired 100 bp

BRM, wt, rep1; total reads: 12,766,558; uniquely mapped reads: 9,020,897; paired 150 bp
BRM, wt, rep2; total reads: 40,870,066; uniquely mapped reads: 11,878,368; paired 150 bp
BRM, KO, rep1; total reads: 30,381,065; uniquely mapped reads: 20,428,477; paired 150 bp
BRM, KO, rep2; total reads: 18,535,463; uniquely mapped reads: 12,149,011; paired 100 bp
BRG1, wt, rep1; total reads: 30,274,681; uniquely mapped reads: 21,863,148; single 100 bp
BRG1, wt, rep2; total reads: 21,750,507; uniquely mapped reads: 17,720,475; single 100 bp

H3K4me1, wt, rep1; total reads: 66,403,032; uniquely mapped reads: 30,990,256; paired 150 bp
H3K4me1, wt, rep2; total reads: 33,783,140; uniquely mapped reads: 27,484,034; paired 150 bp
H3K4me3, wt, rep1; total reads: 59,139,406; uniquely mapped reads: 43,598,634; single 100 bp
H3K4me3, wt, rep2; total reads: 26,186,298; uniquely mapped reads: 14,547,070; paired 150 bp
H3K27me3, wt, rep1; total reads: 40,763,482; uniquely mapped reads: 20,125,878; paired 150 bp
H3K27me3, wt, rep2; total reads: 34,417,180; uniquely mapped reads: 18,317,249; paired 150 bp

CTCF, wt, rep1; total reads: 20,180,276; uniquely mapped reads: 6,446,720; paired 100 bp
CTCF, wt, rep2; total reads: 43,968,488; uniquely mapped reads: 29,171,270; paired 150 bp

RNAPII, wt, rep1; total reads: 46,853,163; uniquely mapped reads: 30,864,453; paired 150 bp
RNAPII, wt, rep2; total reads: 50,918,941; uniquely mapped reads: 38,199,665; paired 150 bp
RNAPII, KO, rep1; total reads: 50,918,941; uniquely mapped reads: 38,199,665; paired 150 bp
RNAPII, KO, rep2; total reads: 18,181,091; uniquely mapped reads: 12,137,827; paired 150 bp

Antibodies

For ChIP-seq: anti-CREST (ProteinTech, 12439-1-AP), anti-CBP (Cell Signaling, 7389S), anti-p300 (Abcam, ab275378), anti-TIP60 (ProteinTech, 10827-1-AP), anti-IgG (Cell Signaling, 2729), anti-BRM (Bethyl, A301-015A), anti-BRG1 (Abcam, ab110641), anti-H3K27ac (Abcam, ab4729), anti-H3K4me1 (Abcam, ab8895), anti-H3K4me3 (Abcam, ab8580), anti-H3K27me3 (Abcam, ab6002), anti-CTCF (Millipore, 07-729), anti-RPB1 (Cell Signaling, 2629).

Peak calling parameters

Binding locations enriched by ChIP-seq were identified using the GeneTrack peak predictor (version 1.0), which was conducted using Galaxy ChIP-seq tools (https://usegalaxy.org/). The following parameter setting was used for peak calling - sigma for smoothing reads (-s): 50, peak exclusion zone (-e): 1000, exclusion zone for upstream: 500, and exclusion zone for downstream: 500.

Data quality

FASTQC (version 0.11.9) is run to check the sequencing quality. Sequencing reads distribution was also checked using RSeQC package (version 3.6).

Software

FASTQC (version 0.11.9); Bowtie2 aligner (version 2.3, Galaxy Mapping tool version from https://usegalaxy.org/); edgeR package (version 3.30, downloaded from Bioconductor); GeneTrack peak predictor (version 1.0, Galaxy ChIP-seq tool version); MACS2 peak caller (version 2.1, Galaxy ChIP-seq tool version).
