# Supplementary material for: CREST functions as a molecular bridge between neuron-specific enhancers and epigenetic regulators in mature motor neurons
Source: Nat Commun. 2026 Oct 10;17:11520. doi: 10.1038/s41467-026-78396-x (PMC13745572; doi:10.1038/s41467-026-78396-x)
Supplement: Supplementary file 4 [file 41467_2026_78396_MOESM4_ESM.pdf]

Description of Supplementary Data Files

Supplementary Data 1 (xlsx file)
List of next-generation sequencing datasets for RNA-seq, ChIP-seq, ChIP-exo, ATAC-seq, DNase-seq, and Hi-C used in this study.

Supplementary Data 2 (xlsx file)
RNA log_2 (FPKM+0.1) values shown in **Fig. 1b**.

Supplementary Data 3 (xlsx file)
Uncropped and unprocessed raw images of all Western blots and DNA agarose gels generated in this study.

Supplementary Data 4 (xlsx file)
List of normalized RNA expression of all mRNA genes, differentially expressed genes, and FPKM values during motor neuron differentiation, used in this study.

Supplementary Data 5 (xlsx file)
List of all gene ontology (GO) target genes and GO background genes used in this study.

Supplementary Data 6 (xlsx file)
List of GO terms and P-values generated in this study.

Supplementary Data 7 (xlsx file)
Individual data points (numerical values) used to generate box plots in this study.

Supplementary Data 8 (xlsx file)
List of CREST-bound and -unbound regions, related to **Fig. 3b**.

Supplementary Data 9 (xlsx file)
List of CREST-bound and stage-specific accessible regions, related to **Fig. 5**.

Supplementary Data 10 (xlsx file)
Summary statistics, including sample means, F statistics, P-values, and associated values used for ANOVA analyses in this study.

Supplementary Data 11 (xlsx file)
Individual numerical data points and Western blot images of biological replicates used to generate box plots in Supplementary Fig. 18.
